# An intelligent feedback loop for sustaining self-lubrication and wear resistance

**DOI:** 10.1038/s41467-026-73957-6

**Published:** 2026-06-02

**Authors:** Fuyan Kang, Shilin Deng, Panpan Li, Rui Zhao, Xiaohong Liu, Hongxuan Li, Huidi Zhou, Jianmin Chen, Wengen Ouyang, Li Ji

**Affiliations:** 1 State Key Laboratory of Solid Lubrication, Lanzhou Institute of Chemical Physics, Chinese Academy of Sciences, Lanzhou, PR China; 2 Center of Materials Science and Optoelectronics Engineering, University of Chinese Academy of Sciences, Beijing, PR China; 3 Department of Engineering Mechanics, School of Civil Engineering, Wuhan University, Wuhan, PR China; 4 School of Mechanical and Electrical Engineering, Xinyu University, Xinyu, PR China; 5 State Key Laboratory of Water Resources Engineering and Management, Wuhan University, Wuhan, PR China

**Keywords:** Mechanical engineering, Nanoscale materials

## Abstract

Intelligent materials that self-sense and self-adjust are an emerging frontier in sustainable technology. Here we introduce a Cu/C nanocomposite film that acts as a self-adjusting intelligent lubricant. In this film, frictional heating triggers melting and migration of Cu nanoparticles along nanopores to the friction interface, where the Cu catalyzes the in-situ formation of ordered carbon nanostructures. Real-time monitoring of friction coefficient (*μ*), electrical resistance (*R*), and metal release confirms a feedback loop: high friction generates enough heat, melting the metal nanoparticles; the migrating metal then lowers friction by creating low-friction nanostructures, which reduces heat and arrests further migration until friction rises again. This self-limiting feedback enables stable ultra-low friction (*μ* ~ 0.04) and an exceptional wear life (>40 km) even in high vacuum. By utilizing friction-derived heat as an intrinsic activation signal, our system establishes a general paradigm for intelligent, self-adjusting materials with applications extending beyond tribology.

## Abstract (web summary)

Intelligent materials are an emerging frontier in sustainable technology. The authors introduce Cu/C films as a class of intelligent lubricants that demonstrate capabilities including self-sensing, self-adjustment, and self-repairing.

## Introduction

Intelligent materials represent a frontier in sustainable technology, with the global market projected at 72.36 billion dollars in 2023, and it is expected to reach 133.1 billion dollars by 2030^1^. These materials offer exceptional energy-saving potential and performance advantages across fields, such as advanced manufacturing^2–5^, biomedical engineering^6–8^, and aerospace^9,10^. In tribology, intelligent lubricants are particularly critical, as they adaptively reduce friction and wear under varying conditions–addressing a key limitation of conventional lubricants^11–15^. For example, carbon materials—a major class of lubricating materials—feature carbon atoms held together by covalent bonds and possess abundant defects and dangling bonds, which become more pronounced during friction. They undergo strong chemical interactions with environmental molecules and the counterpart, rendering their lubrication performance highly dependent on experimental conditions.

Current design strategies–such as biomimetic structures^16–20^, microencapsulation^21–23^, stimulus-responsive molecules^24–26^, and porous structures^27,28^–have led to significant advances. Yet, most systems still operate through passive, mechanical responses without self-sensing and self-adjusting capabilities. For instance, some materials aim to use the stability of interface structures to achieve adaptability^29^, or some self-healing lubricants require external force or light to release healing agents, often resulting in over-release^22^ and inefficient performance^27,30^. This results in the lubricating material being able to only achieve one-time use in the aforementioned situation, without feedback loop. Recent studies have revealed that elements, such as Cu and Au exhibit unique catalytic properties towards carbon, enabling the formation of ordered carbon lubricating structures at the friction interface^31^, and that, when doped into carbon matrices, these elements can migrate to the surface during dynamic friction^32,33^, where they modulate the lubricating interface, thereby offering new possibilities for the design of intelligent lubricating materials. Nevertheless, realizing lubricants with self-sensing and self-adjusting capabilities remains an unmet challenge^34^–one whose solution could extend material lifetimes and enable operation in extreme environments, such as vacuum.

This study reveals an intelligent lubrication mechanism in a Cu/C nanocomposite film driven by a self-adjusting feedback loop of friction-induced metal migration and catalytic carbon restructuring. Combining real-time measurements of friction coefficient, electrical resistance, and metal release, and simulation analysis, we demonstrate that frictional heating triggers solid-to-liquid transitions of Cu nanoparticles (NPs) followed by directed migration through nanopores to the friction interface. Once there, the Cu NPs catalyzes the formation of low-shear carbon nanostructures that rapidly lower interfacial friction and suppress further heating and migration, producing a self-limiting cycle. This intelligent system achieves unprecedented wear life (>40 km) and ultralow friction (*μ* ~ 0.04) in vacuum, resolving the long-standing failure problem of carbon materials under such conditions. Our work offers a general design strategy for intelligent materials with broad interdisciplinary impact.

## Results

### Real-time monitoring intelligent lubrication in the Cu/C film

**Fig. 1:**
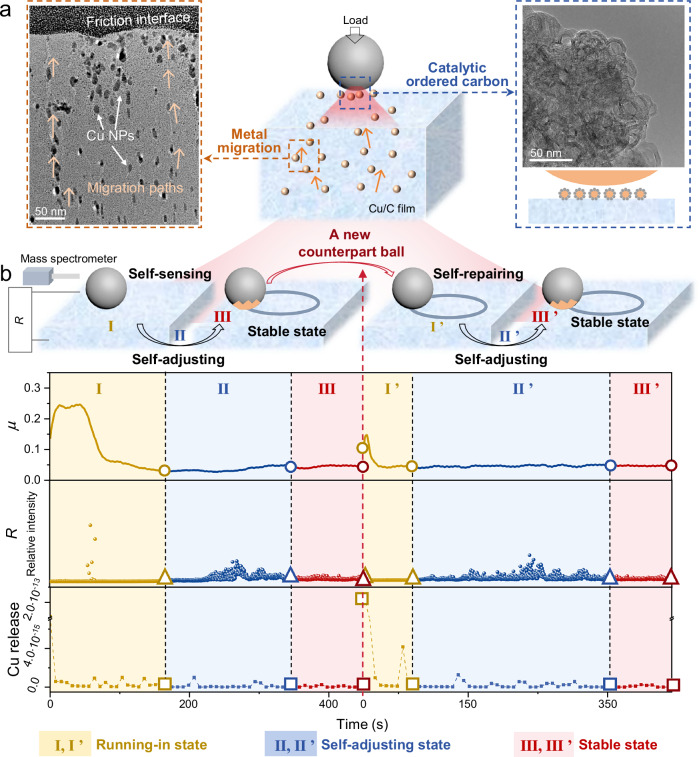
Intelligent lubrication behavior of the Cu/C film and real-time observation. **a** High-resolution transmission electron microscopy (HRTEM) observation of the internal and interface microstructures of the Cu/C film at the wear track; **b** Real-time monitoring results of the friction coefficient (*μ*), electrical resistance (*R*) and Cu release during two consecutive friction processes of the Cu/C film (The values of Cu release refer to the amount of metal released per unit time, expressed in the unit of Torr·s^-1^).

We observe a distinctive phenomenon during the friction of the Cu/C nanocomposite film: Cu NPs migrate within the material. As shown in Fig. 1a, the Cu NPs adopt droplet-like shapes and travel along nanoscale pores toward the friction interface. Concurrently, the friction interface itself becomes decorated with numerous ordered carbon nanostructures, which are known to facilitate low friction^32,35,36^. This dynamic restructuring internally and at the friction interface gives rise to an intelligent lubrication behavior, captured in real time through synchronous measurements of friction coefficient, electrical resistance, and Cu release (Fig. 1b and Supplementary Figs. 1 and 2).

The process unfolds in three distinct stages. In the initial running-in stage (I), before a stable lubricating interface forms, the friction is high. This state stimulates substantial Cu NP migration toward the surface, reflected by sharp fluctuations in electrical resistance and continuous Cu release. Once a primary lubricating interface of ordered carbon is established, the system enters a self-adjusting stage (II). Here, minor increases in the friction coefficient trigger slight, corrective replenishment via Cu migration, evidenced by small, correlated fluctuations in electrical resistance and Cu release. Ultimately, a robust, ordered carbon interface is maintained in a stable state (III), where friction remains low and stable, accompanied by steady electrical resistance and a near-constant, low level of Cu release over extended periods.

Furthermore, the system demonstrates a self-repair capacity. Introducing a new counterpart ball disrupts the friction interface, causing the system to swiftly repeat the cycle from stage I to III, thereby repairing the lubricating interface. Throughout this process, the qualitative coupling between friction coefficient, electrical resistance, and metal release is clear and repeatable, demonstrating the film’s integrated self-sensing, self-adjusting, and self-repairing functions. We note that the quantitative correlations between these signals are complex (Supplementary Fig. 3), likely due to the multifaceted nature of friction and inherent detection lags, a point we explore in subsequent analysis.

### Frictional heat-induced solid-liquid phase transition and migration mechanism of Cu NPs

**Fig. 2:**
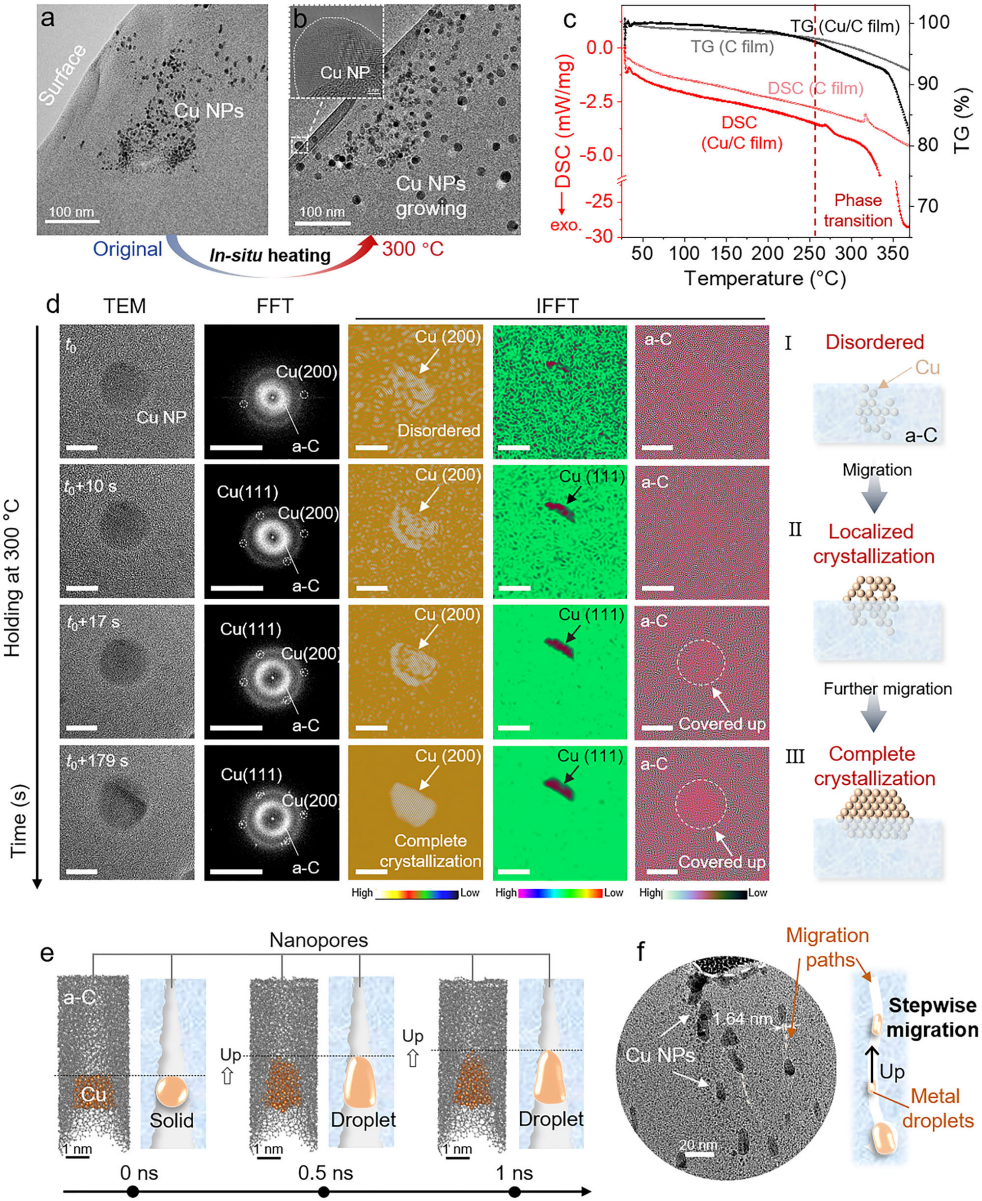
Migration mechanism of heat-induced solid-liquid phase transition of Cu NPs. **a**,**b** In-situ HRTEM images of the Cu/C film during heating from room temperature to 300 °C, **c** DSC and TG curves of C and Cu/C films, **d** HRTEM images, fast Fourier transform (FFT) and IFFT patterns of Cu (200), Cu (100), and a-C, as well as schematic diagram of the microstructural evolution of the Cu/C film during holding at 300 °C (Scale bars in the TEM and IFFT images are 10 nm, while scale bars in FFT images is 10 1/nm), **e** MD simulations and schematic diagram of a Cu NP migrating along the nanopores, and **f** Cross-sectional TEM image of the wear track and schematic diagram of stepwise migration of Cu droplets.

We investigated the mechanism driving Cu NPs migration during friction, a process where mechanical force and thermal effects coexist. To isolate these factors, a static pressure test first confirmed that mechanical stress alone is insufficient to initiate migration (Supplementary Fig. 4). In contrast, heat treatment readily induced this process (Supplementary Fig. 5). We therefore focused on the thermally driven mechanism using in-situ transmission electron microscopy (TEM). Our observations revealed that upon heating, initially dispersed, tiny Cu NPs (2–5 nm in size) within the amorphous carbon (a-C) matrix continuously aggregated and grew into larger NPs (15–20 nm) by 300 °C (Fig. 2a, b and Supplementary Figs. 6, 7). This growth indirectly demonstrates Cu migration, as atoms move along the shortest path—in this thin sample geometry, parallel to the electron beam. Direct observation of lateral migration was only possible for Cu NPs near the sample edge (Fig. 2b). Notably, all Cu NPs adopted a spherical morphology at 300 °C, may suggesting a liquid state governed by surface tension. At 400 °C, some NPs appeared to gradually disappear, suggesting possible evaporation (Supplementary Fig. 8).

To directly analyze this thermal phase transition, we performed a differential scanning calorimetry-thermogravimetry (DSC-TG) test. Compared to the C film, the Cu/C film exhibited a distinct endothermic peak beginning around 270 °C, accompanied by greater mass loss (Fig. 2c), which is consistent with Cu NP melting. Subsequent X-ray photoelectron spectroscopy (XPS) analysis of the post-DSC-TG-test sample showed no detectable Cu signal (Supplementary Fig. 9), confirming NP volatilization and explaining the in-situ TEM observations at 400 °C. Together, these results verify a solid-liquid phase transition for Cu NPs during heating. This transition is size-dependent; as the melting point decreases for smaller particles^37^, modest frictional heat can melt nanoscale Cu, while subsequent growth at a specific temperature causes re-solidification. This reversible phase transition, directly visualized here, offers a promising method for precisely correlating nanoparticle size with melting point.

The liquid-solid phase transition process of the Cu NPs was captured by in-situ TEM during holding at 300 °C (Fig. 2d). According to the inverse fast Fourier transform (IFFT) patterns, at the time of *t*_0_, most zone is disordered corresponding to liquid phase. Over time, the crystallinity of both Cu (200) and Cu (111) crystal planes increases progressively. By *t*_0+179 s_, the Cu (200) and Cu (111) crystal planes are completely crystallized, demonstrating that the molten Cu NP solidifies due to reaching the critical growth size under isothermal conditions. The contrast variations of a-C indicate that the liquid Cu NP migrates toward the surface while continuously solidifying, as illustrated schematically, exhibiting a solid-liquid-solid phase transition loop. To further elucidate the underlying migration physics, molecular dynamics (MD) simulations show that a heated Cu NP undergoes a solid-liquid phase transition and gradually moves toward the surface along the nanopore, driven by a Laplace pressure gradient arising from the nanopore geometry^38^ (Fig. 2e and Supplementary Fig. 10 and Supplementary Movie 1). This migration mechanism is corroborated by cross-sectional TEM imaging of the wear track, where elliptical Cu droplets are clearly observed (Fig. 2f).

Overall, the process of Cu migration during friction can be understood as follows. During the friction process, a gradient temperature distribution^39^ that is formed across the friction interface further modulates this process, giving rise to stepwise migration. Initially dispersed small Cu NPs melt and migrate toward the surface while concurrently coalescing into larger aggregates, leading to a characteristic size gradient due to the solid-liquid-solid phase transition loop. Although sufficient frictional heat can melt large aggregates, the narrow nanopores allow only smaller droplets to pass through, which can intelligently regulate Cu supplementation. Consequently, larger NPs solidify and remain embedded as metal reservoirs, preserving the film’s mechanical stability (Supplementary Fig. 11). These findings establish the phase transition kinetics and transport mechanisms governing metal migration in solid matrices, providing fundamental insights for designing self-regulating metal-carbon composites with tailored surface architectures under thermal and tribological stimulation.

### Cu NPs catalyze the formation of low-friction ordered carbon nanostructures

**Fig. 3:**
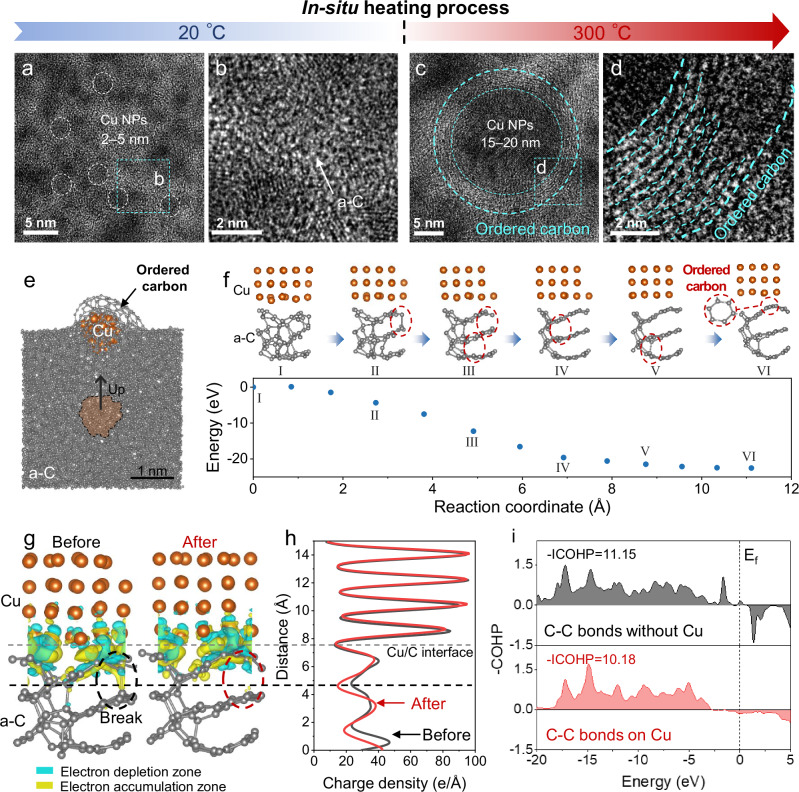
Cu catalysis forms ordered carbon nanostructures and catalytic mechanisms. **a–d** In-situ HRTEM images of the Cu/C film during heating, **e** MD simulations of the formation of ordered carbon nanostructures during the process of the Cu NP migrating to the surface, **f** Energy changes in the Cu-catalyzed transformation from a-C to ordered carbon (Ⅰ. a-C; Ⅱ-Ⅴ*. sp*^3^C breakage and *sp*^2^C recombination; Ⅵ. Ordered carbon), **g** DCD maps of the Cu-C configuration before and after the C-C bond breaking during Cu catalysis, **h** Charge density distribution before and after C-C bond breakage, and **i** Averaged -COHP of C–C bonds with and without Cu NPs (the lower the absolute value of -ICOHP, the weaker the C–C bond strength).

Building on the observation that numerous ordered carbon nanostructures form at the friction interface (Fig. 1a), a phenomenon noted in previous researches^35,40–43^, we investigated their formation mechanism through in-situ high-resolution TEM and theoretical simulations. Our in-situ heating experiments reveal a dynamic process where initially dispersed, small Cu NPs (2–5 nm) progressively agglomerate into larger clusters (15–20 nm) with rising temperature (Fig. 3a–d and Supplementary Movie 2). Crucially, at 300 °C, the carbon matrix at the boundary of these Cu NPs transforms from an amorphous to an ordered state, forming multiple concentric layers that encapsulate the metal core (Fig. 3c, d and Supplementary Fig. 12). This catalytic encapsulation is corroborated by MD simulations, which show the formation of ordered carbon shells around a Cu NP as it migrates toward the surface (Fig. 3e). The resulting spherical, closed carbon nanostructures^44^ are known to facilitate low friction via rolling lubrication^45–47^ and passivating reactive carbon dangling bond^31,48,49^.

To elucidate the atomic-scale catalytic mechanism, we turned to ab initio molecular dynamics (AIMD) simulations and density functional theory (DFT) calculations. AIMD simulations of the Cu-C interface show C atoms on the Cu surface progressively transitioning from tetrahedral *sp*³ to graphitic-like *sp*² coordination (Supplementary Fig. 13 and Supplementary Movie 3), which is prone to form ordered carbon nanostructures. The climbing-image nudged elastic band (CI-NEB) method maps the transition pathway, confirming the *sp*³-to-*sp*² conversion proceeds via C–C bonds breaking and reformation along a monotonically decreasing energy profile (Fig. 3f). Differential charge density analysis further reveals pronounced electronic redistribution during this process, with the C–C bonds prior to cleavage located in regions of charge accumulation, followed by substantial charge reorganization after bond breaking (Fig. 3g, h and Supplementary Fig. 14). This highlights an electronic origin for the Cu-catalyzed structural transformation. To quantify the Cu-induced electronic effect, we computed the crystal orbital Hamilton population (COHP) of interfacial C–C bonds with and without Cu NPs. The presence of Cu gives rise to pronounced antibonding contributions near the Fermi level in the C–C COHP spectrum (Supplementary Figs. 15 and 16), indicating strong electronic interactions between Cu and neighboring carbon atoms. This effect stems from the lower work function of Cu, which promotes electron donation at the Cu–C interface (Supplementary Fig. 17). The corresponding reduction in the average -ICOHP value of C–C bonds indicates a weakening of the C–C bonding strength in the presence of Cu (Fig. 3i), leading to a loosening of the carbon network^50^. This weakening facilitates C-C bond cleavage during thermal vibration, ultimately leading to the formation of thermodynamically more stable *sp*^2^-hybridized carbon structures on the surface of Cu NPs.

### Feedback mechanisms for intelligent lubrication of the Cu/C film

**Fig. 4:**
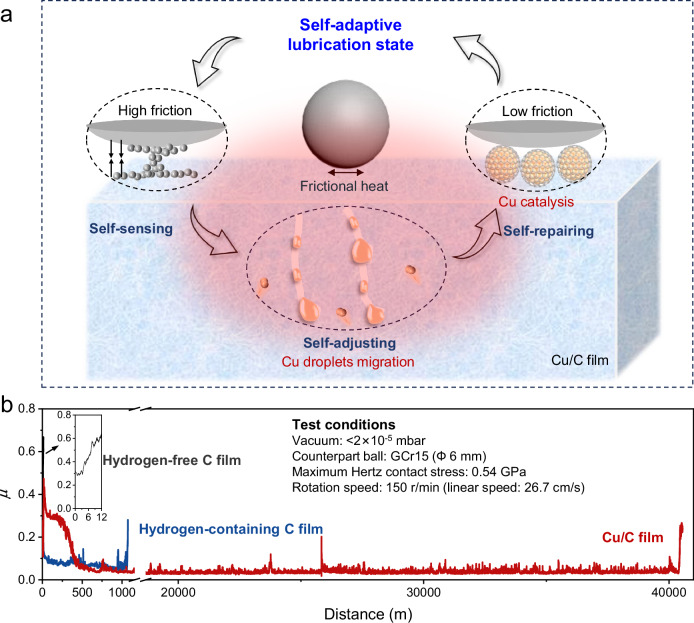
Feedback mechanism of intelligent lubrication of the Cu/C film and the lubricating performance in vacuum. **a** Schematic diagram of the feedback mechanism of the intelligent lubrication system powered by frictional heat and **b** Wear life of the hydrogen-free C film, hydrogen-containing C film and Cu/C film.

Based on the above findings, we propose a complete feedback mechanism that enables the intelligent lubrication of the Cu/C film (Fig. 4a). The cycle is initiated by high interfacial friction, stemming from strong interactions among carbon dangling bonds^51^, generating substantial frictional heat^52^. Cu migration can be effectively regulated in response to frictional heat owing to the size-dependent melting point of Cu NPs^37^. Sufficient frictional heat melts Cu NPs within the Cu/C film, triggering to rapid Cu migration toward the friction interface, while insufficient frictional heat prevents this phase transition and migration. The migrating Cu NPs can further form low-friction nano-structures under catalysis thereat, thereby reducing friction and frictional heat. When low-friction nano-structures is worn away or damaged by external factors, the friction interface reverts to a high-friction and high-friction-heat state, initiating a new cycle of friction to exhibit a self-adaptive lubrication state. This allows for achieving more advanced intelligent lubrication behavior, including self-sensing, self-adjusting, and self-repairing. It is noteworthy that this design utilizes frictional heat (friction byproducts) to provide energy without overconsumption, with significant implications for energy savings.

The intelligent feedback lubrication mechanism underpins the exceptional tribological performance of Cu/C film, sustaining an ultralow friction coefficient (*μ* ~ 0.04) and an ultra-long wear life of over 40 km in vacuum, as depicted in Fig. 4b. In contrast, the hydrogen-free C film and the hydrogen-containing C film^33^ fail rapidly after only about 6 m and 1053 m, respectively. Moreover, the Cu/C film exhibits an extremely low wear rate (7.61×10^-8^ mm^3^/Nm), an order of magnitude lower than that of the hydrogen-containing C film (5.64×10^-7^ mm^3^/Nm).

To quantitatively understand the sustainability of the metal reservoir during prolonged cycling, the Cu consumption rate and wear track depth of the Cu/C film at different friction stages are characterized in Supplementary Fig. 18. During the initial running‑in stage, the effective lubricating interface has not yet fully formed. The process is dominated by mechanical wear, leading to significant wear and requiring substantial metal migration until a stable lubricating interface is established. Thereafter, the lubrication state at the friction interface gradually stabilizes (Supplementary Fig. 19), reaching a dynamic balance between metal replenishment and consumption at the frictional interface. Even if the lubrication state occasionally deteriorates, only a small amount of Cu metal needs to be supplied to the frictional interface to maintain the lubrication state. The results of the wear track of the Cu/C film, wear debris, and wear scar of the counterpart ball after long-duration test (Supplementary Fig. 20) also provide direct evidence supporting the proposed mechanistic framework. The dynamic balance between metal replenishment and consumption is key for this intelligent feedback loop, where the high sliding speed is beneficial to migration ability, and the high sliding frequencies result in high consumption, thus leading to certain adaptabilities to test conditions such as linear reciprocating frequencies (Supplementary Fig. 2). Finally, replacing Cu with lower-melting, highly catalytic metals (e.g., Au) should speed migration and promote formation of ordered lubricating layers, offering a straightforward route to enhance the responsiveness of the feedback mechanism.

## Discussion

In summary, we have developed a Cu/C nanocomposite film as an intelligent lubricant capable of autonomous tribological regulation, demonstrated through real-time monitoring of friction coefficient, electrical resistance, and metal release, which reveals its self-sensing, self-adjusting, and self-repairing capabilities. The fundamental feedback mechanism, identified via in-situ experiments and simulations, is driven by frictional heat, inducing a solid-to-liquid phase transition in Cu nanoparticles, enabling their migration along nanopores to the friction interface, where they catalyze the formation of low-friction carbon nanostructures. This self-limiting cycle enables outstanding tribological performance, sustaining an ultralow friction (*μ* ~ 0.04) and an ultra-long wear life of over 40 km in vacuum, thereby overcoming the long-standing problem of rapid failure in carbon-based lubricants under such conditions. By converting frictional energy into a driver for autonomous surface regulation, this work establishes a general design paradigm for intelligent materials with broad potential in tribology, sensing, corrosion protection and advanced manufacturing.

## Methods

### Film preparation

The hydrogen-free C film, hydrogen-containing C film, and Cu/C film were prepared using a magnetron sputtering system (PlasMag CF-800, Teer, U.K.) through co-sputtering from graphite and metal targets. The films were deposited on Si wafers, 304 stainless steel (06Cr19Ni10) and M2 high-speed steel (W6Mo5Cr4V2). Before deposition, the substrates were ultrasonically cleaned with anhydrous ethanol and acetone, then dried with dry N_2_. The deposition process and information about the films are shown in Supplementary Fig. 21.

### Friction and wear tests

Friction and wear tests were conducted using a ball-on-disk tribometer (HVTRB, CSM, Anton Paar, Switzerland) in vacuum (pressure <2×10^-5^ mbar) under rotating mode at a linear speed of 26.7 cm/s and a maximum Hertz contact stress of 0.54 GPa. GCr15 steel balls (Ф6 mm, AISI52100, roughness ≈ 20 nm) were used as counterpart balls. To investigate the intelligent lubrication behaviors of the Cu/C film, a mass spectrometer (PrismaPro, Pfeiffer, Germany) and a multimeter (8845 A/8846 A, Fluke, USA) were connected outside the tribometer chamber to detect the metal release and the electrical resistance during friction. The reciprocating module of the tribometer was adopted for friction tests at an amplitude of 5 mm and different linear reciprocating frequencies (1 Hz for Fig. 1b), using the same GCr15 counterpart balls described above. The morphology of wear scars was observed by optical microscopy (STM6, OLYMPUS, Japan). The elemental composition of wear scars was identified through equipped with energy-dispersive X-ray spectroscopy (EDS, X-max 80, Oxford instrument, The United Kingdom). The depths of wear tracks were measured via the three-dimensional white light interferometry (UP-Lambda, Rtec-Instruments, America) and the wear rates were further calculated from these data. The Cu content in the wear tracks at different friction stages was detected by EDS (EDS, EDAX, America). The Cs-corrected TEM (Spectra 300, Thermo Fisher Scientific, America) was applied to observe the morphology of wear debris from the Cu/C film. The cross-sectional morphology of the characteristic wear tracks was examined through TEM (TECNAI G2 F20, FEI, America). The TEM cross-sectional samples were prepared using a focused ion beam (FIB, Helias 5UX, Thermoscientific, America) method. The corresponding elemental composition was analyzed using EDS-equipped equipment.

### Vacuum annealing tests

Annealing experiments were performed using the heating chamber of the ball-on-disk tribometer (HVTRB, Anton Paar, Switzerland) in vacuum (pressure <2×10^-5^ mbar), The samples were held at 100 °C, 200 °C, and 300 °C for 3 h each to study the metal migration behavior.

### Mechanical performance tests

The mechanical properties of the Cu/C film were tested by nanoindentation (NHT2, CSM, Anton Paar, Switzerland) and a CSM scratch meter (RSTNHT2, Anton Paar, Switzerland). For details, see Supplementary Figs. 11 and 22.

### Characterization

The original structure of the Cu/C film is shown in Supplementary Fig. 23. The crystal structure information was obtained by X-Ray Diffraction (XRD, Empyrean, Malvern Panalytical, Netherlands). The carbon structure information was tested by Raman spectroscopy (Renishaw, UK, wavelength = 532 nm). The dynamic structure evolution of a-C during friction was online investigated through in-situ Raman spectroscopy (wavelength = 532 nm). A three-dimensional white light interferometry (MicroXAM-3D, ADE, America) was used to measure the film thickness. The FESEM (SU8020, HITACHI, Japan) was used to observe the surface morphology of the films before and after annealing at different temperatures. The elemental composition of the surface after annealing at 300 °C was tested by point scanning using the equipment equipped with EDS (EDS, EDAX, America). Ex-situ XPS (ESCALAB 250Xi, Thermo Fisher, America) was used to analyze the elemental content of the Cu/C film, powders derived from the Cu/C film before and after DSC-TG testing, and the contact area before and after the static pressure test. The elemental analysis of the Cu/C film was further characterized by in-situ XPS (VersaProbe 4, PHI, Japan) at different temperatures during heating. The migration behavior of Cu NPs in a-C matrices was observed by in-situ TEM (JEM-ARM300F2, JEOL, Japan) at 25 frames per second, with an electron beam voltage of 300 kV. The in-situ TEM system was equipped with an electric heating holder, yielding a temperature accuracy of ± 10 °C. The in-situ TEM samples were prepared from the Cu/C film using FIB (Helios Nanolab 600i, Thermo Scientific, America). The melting point of Cu NPs in the Cu/C film was determined by the DSC-TG test (STA 449 F3 Jupiter, Netzsch, Germany). Ar gas flow was introduced before heating, followed by heating at 10 °C/min, holding for 5 min, and then cooling at the same rate.

### Development of the neuroevolution potential (NEP) model

The NEP model^53,54^ writes the site energy of a central atom *i* as:

1 $${U}_{i}\left(\{{q}_{\nu }^{i}\}_{\nu=1}^{{N}_{{\rm{des}}}}\right)=\mathop{\sum }\limits_{\mu=1}^{{N}_{\rm{neu}}}\,{w}_{\mu }^{(1)}\tanh \left(\mathop{\sum }\limits_{\nu=1}^{{N}_{\rm{des}}}\,{w}_{\mu \nu }^{\left(0\right)}{q}_{\nu }^{i}-{b}_{\mu }^{\left(0\right)}\right)-{b}^{\left(1\right)}$$

where $\tanh (*)$ denotes the activation function of the hidden layer, ${N}_{{\rm{des}}}$ denotes the number of dimensions of the descriptor vector, and ${N}_{{\rm{neu}}}$ denotes the number of neurons in the hidden layer of a single layer neural network. The parameters ${w}^{(0)}$, ${w}^{(1)}$, ${b}^{(0)}$, and ${b}^{(1)}$ are the weight and bias parameters to be optimized during the training process. The descriptor ${\left\{{q}_{\nu }^{i}\right\}}_{\nu=1}^{{N}_{{des}}}$ consists of radial and angular components.

To develop a NEP model capable of accurately describing Cu–C interactions, we started from the C–C dataset constructed by Wang et al.^55^ and substantially extended it by building dedicated Cu–C and Cu reference datasets tailored for this work. As schematically illustrated in Supplementary Fig. 24, the dataset comprises pure C structures, pure Cu configurations (including bulk and surface slabs), Cu–C bulk mixtures with varying compositional ratios, Cu–C interfacial and surface models, as well as Cu clusters interacting with carbon frameworks. The Cu–C bulk mixtures were sampled from AIMD simulations conducted at temperatures between 300 K and 800 K under the NPT ensemble using a Langevin thermostat, while Cu–C surface models, together with bulk Cu and Cu surface slabs, were sampled from AIMD simulations under the NVT ensemble using a Nosé-Hoover thermostat over the same temperature range. After establishing the datasets, DFT single-point energy calculations were performed using the Vienna Ab-initio Simulation Package (VASP)^56,57^, employing the projected augmented Wave (PAW) method and the Perdew-Burke-Ernzerhof (PBE) functional^58^. The calculations used a plane-wave energy cutoff of 550 eV, a *k*-point spacing of 0.2 /Å, and an energy convergence threshold of 10^-6^ eV. The final DFT reference dataset comprised 1644 configurations, which were randomly divided into a training set of 1480 configurations and a validation set of 164 configurations. The training results of the NEP model are presented in Supplementary Fig. 25, and the hyperparameters for training are listed in Supplementary Table 1.

### Model and set-up for MD simulations

Our MD simulations comprised three parts: (i) migration of metal NPs within a quasi-conical pore in an a-C model, (ii) guided metal NP motion to probe catalytic behavior, and (iii) AIMD simulations to explore Cu-C interfacial interactions. For metal migration, a quasi-conical pore (base diameter ≈ 3 nm; top diameter ≈ 1 nm; Supplementary Fig. 10a) was carved into the a-C model (model size ≈ 3 × 3 × 7 nm^3^; Supplementary Fig. 26) with a 227-atom Cu NP placed near the pore entrance. The initial structure was energy-minimized with the FIRE method (force <10^-^⁶ eV/Å) and further equilibrated at 800 K for 1 ns while fixing the bottom 1 nm in the NVT ensemble (Nosé-Hoover thermostat, 1 fs timestep). For metal migration behavior, simulations were performed with the GPU-accelerated molecular dynamics (GPUMD) package^53^. To study the catalysis effect of migrating metal NPs, the metal NP was driven at 5 m/s along the pore axis using the Large-scale Atomic/Molecular Massively Parallel Simulator (LAMMPS)^59^. The simulation utilized a 1 fs timestep, and a Nosé-Hoover thermostat at 300 K. Interfacial interactions during the Cu-catalyzed ordered carbon were further examined by AIMD in VASP (PBE functional, 500 eV cutoff, 10⁻⁴ eV energy convergence, and a 1× 1× 1 *k*-point mesh). NVT simulations were run at 800 K with a 3 fs timestep for 30 ps. The initial model comprised an amorphous *sp*³-rich carbon substrate (66 atoms) with a 32-atom Cu NP on top in a 0.7 × 0.7 nm² cell with 3 nm vacuum spacing along the *z*-axis.

### Model and set-up for DFT calculations

All DFT calculations were performed using VASP^56,57^ with the PBE^58^ functional and a plane-wave energy cutoff of 500 eV. Atomic positions were fully relaxed until residual forces were below 0.01 eV/Å and total energies were converged to 10^-^⁴ eV. Brillouin-zone sampling employed a 7 × 7 × 1 Monkhorst-Pack grid.

The differential charge density distribution was calculated by:

2 $${\rho }_{{\rm{diff}}}\left(x,y,z\right)={\rho }_{{\rm{tot}}}\left(x,y,z\right)-{\rho }_{{\rm{Cu}}}\left(x,y,z\right)-{\rho }_{{\rm{C}}}\left(x,y,z\right)$$

where ${\rho }_{{\rm{tot}}}(x,y,z)$, ${\rho }_{{\rm{Cu}}}(x,y,z)$ and ${\rho }_{{\rm{C}}}(x,y,z)$ are the charge density distribution of Cu-C, Cu and C.

The CI-NEB method was used to map the reaction energy landscape, with the optimized pre- and post-catalytic Cu-C structures as the initial and final states, respectively. The reaction path was discretized using 11 images, and the spring constant was set to 5.0 eV/Å². The work function was calculated to characterize the electronic properties of the constructed Cu-C model. The Cu slab was cleaved using a 2 × 2 × 2 surface supercell, with 4 atomic layers and ~20 Å of vacuum along the *z*-axis. The bottom two layers were fixed to emulate bulk termination, while the top two layers were relaxed until the residual forces were below 0.01 eV/Å. Brillouin-zone sampling used a 7 × 7 × 1 Monkhorst-Pack grid. Additionally, we employed an a-C slab representative of the substrate. The final slab contained ~66 atoms in the active region, with an in-plane cross-section ~7.5 × 7.5 Å^2^ and ~35 Å of vacuum. All calculations were performed using DFT with the generalized gradient approximation PBE (GGA-PBE) as implemented in the VASP. The work function was extracted as

3 $$\Phi={E}_{{\rm{vac}}}-{E}_{{\rm{Fermi}}}$$

where ${E}_{{\rm{Fermi}}}$ denotes the Fermi level of the system. ${E}_{{\rm{vac}}}$ denotes the vacuum energy level of the system.

The COHP analysis was performed to quantify bonding and antibonding interactions between specific atom pairs. Single-point wavefunctions from VASP were post-processed with the Local Orbital Basis Suite Towards Electronic-Structure Reconstruction (LOBSTER)^60^, which projects plane-wave states onto localized orbital bases for electronic-structure analysis.

### Reporting summary

Further information on research design is available in the Nature Portfolio Reporting Summary linked to this article.

## Supplementary information

Supplementary information

Description of Additional Supplementary File

Supplementary Movie 1

Supplementary Movie 2

Supplementary Movie 3

Reporting Summary

Transparent Peer Review file

## Source data

Source Data

## Data Availability

The data supporting the findings of this study are available within the main text and supplementary information files/Source Data file. Source data are provided with this paper.
