# Supplementary material for: An intelligent feedback loop for sustaining self-lubrication and wear resistance
Source: Nat Commun. 2026 Jun 2;17:7071. doi: 10.1038/s41467-026-73957-6 (PMC13392075; doi:10.1038/s41467-026-73957-6)
Supplement: Supplementary file 1 — Supplementary information [file 41467_2026_73957_MOESM1_ESM.pdf]

Supplementary information

An intelligent feedback loop for sustaining self-lubrication and wear resistance

Fuyan Kang[1,2,#], Shilin Deng[3,#], Panpan Li[1,#], Rui Zhao[4], Xiaohong Liu[1,2], Hongxuan Li[1,2,*], Huidi Zhou[1], Jianmin Chen[1], Wengen Ouyang[3,5,*] & Li Ji[1,2,*]

[1] State Key Laboratory of Solid Lubrication, Lanzhou Institute of Chemical Physics, Chinese Academy of Sciences, Lanzhou, Gansu, 730000, P. R. China.

[2] Center of Materials Science and Optoelectronics Engineering, University of Chinese Academy of Sciences, Beijing, 100049, P. R. China.

[3] Department of Engineering Mechanics, School of Civil Engineering, Wuhan University, Wuhan, Hubei, 430072, P. R. China.

[4] School of Mechanical and Electrical Engineering, Xinyu University, Xinyu, 338004, P. R. China.

[5] State Key Laboratory of Water Resources Engineering and Management, Wuhan University, Wuhan, Hubei, 430072, P. R. China.

[#] These authors contributed equally to this study.

[*] Corresponding author e-mails: lihx@licp.cas.cn; w.g.ouyang@whu.edu.cn; jili@licp.cas.cn

This Supplementary information file includes:

Supplementary Note 1: The intelligent lubrication behavior of the Cu/C film. 2

Supplementary Note 2: Heat-induced Cu migration. 4

Supplementary Note 3: The studies of Cu migration mechanism. 7

Supplementary Note 4: The mechanism of ordered carbon formation catalyzed by Cu NPs. 9

Supplementary Note 5: Friction interfacial analysis of the Cu/C film. 12

Supplementary Note 6: The film preparation and information. 14

Supplementary Note 7: Development of the NEP model and set-up for MD simulations. 16

Supplementary References 18

Supplementary Note 1: The intelligent lubrication behavior of the Cu/C film.

A custom real-time detection setup was used to monitor the intelligent lubrication behavior of the Cu/C film, as depicted in Supplementary Fig. 1. A mass spectrometer and a multimeter connected to the tribometer chamber enabled the synchronous detection of metal release and electrical resistance during sliding.

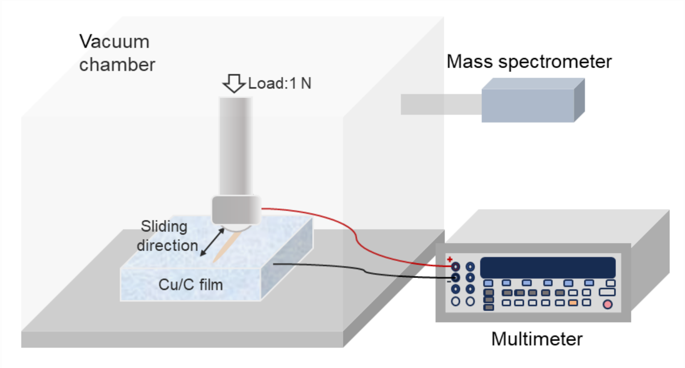

Supplementary Fig. 1| The schematic diagram of the real-time detection device.

Supplementary Fig. 2 shows friction coefficient curves and the corresponding electrical resistance of the Cu/C film at different reciprocating frequencies. At 1.0, 1.5, and 2.0 Hz, the electrical resistance exhibits intermittent changes. When the frequency reaches 3 Hz, the electrical resistance remains high throughout the entire test. Concurrently, the running-in time of the Cu/C film shortens progressively as the reciprocating frequency increases.

These results indicate that a higher reciprocating frequency corresponds to a higher sliding speed and more intense frictional heating, which rapidly drives Cu migration to establish a low-friction state. This is evidenced by the shortening running-in time. Simultaneously, a high frequency leads to more cycles per unit time (a high consumption rate), which prevents the lubricating interface from stabilizing and necessitates continuous replenishment. This is reflected in the electrical resistance behavior: at 3 Hz (6 cycles/s), pronounced fluctuations indicate sustained metal migration, leading to rapid depletion of the metal reservoir and eventual lubrication failure. At frequencies below 3 Hz, intermittent electrical resistance changes signify that metal migration reaches a dynamic equilibrium and is periodically regulated.

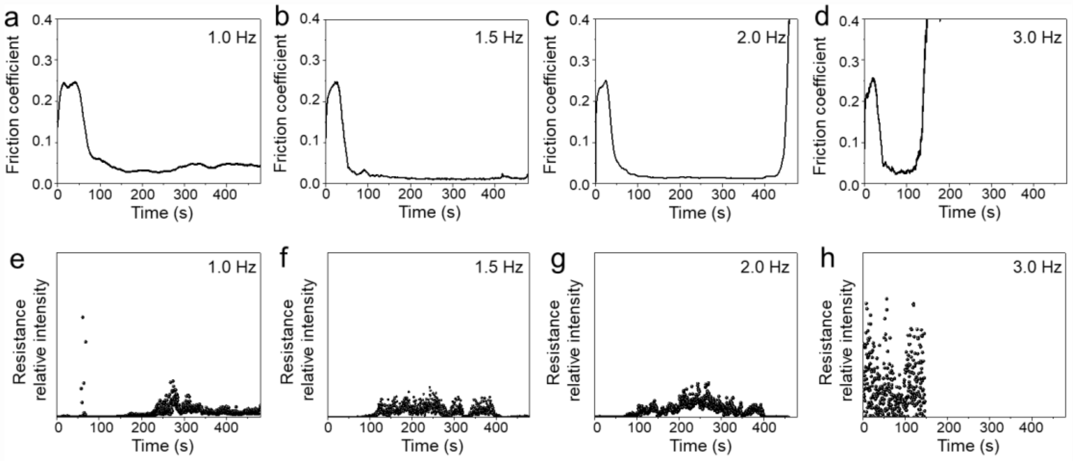

Supplementary Fig. 2| Friction coefficient curves and corresponding electrical resistance of the Cu/C film at different linear reciprocating frequencies. (**a, e**) 1.0 Hz. (**b, f**) 1.5 Hz. (**c, g**) 2.0 Hz. (**d, h**) 3.0 Hz.

The associations among the friction coefficient (μ), electrical resistance (R), and Cu release were quantified using the Spearman's rank correlation coefficient. For two time series x and y sampled at paired time points, Spearman's coefficient is defined as the Pearson correlation of their rank-transformed variables[1]:

$$\rho_s = \frac{\sum_{i=1}^{n}\left(r(x_i)-\overline{r(x)}\right)\left(r(y_i)-\overline{r(y)}\right)}{\sqrt{\sum_{i=1}^{n}\left(r(x_i)-\overline{r(x)}\right)^2}\sqrt{\sum_{i=1}^{n}\left(r(y_i)-\overline{r(y)}\right)^2}} \quad (1)$$

where ρ_s is Spearman's rank correlation coefficient; n is the number of paired observations; x_i and y_i are the i-th samples of variables x and y, respectively; $r(x_i)$ and $r(y_i)$ denote the ranks of x_i and y_i, respectively. The rank operator $r(\cdot)$ assigns the rank in the presence of ties, defined as

$$r(x_i) = 1 + \sum_{j=1}^{n} 1(x_j < x_i) + \frac{1}{2}\sum_{j=1}^{n} 1(x_j = x_i) \quad (2)$$

and analogously for $r(y_i)$, where $r(\cdot)$ is the indicator function.

The mean ranks are

$$\overline{r(x)} = \frac{1}{n}\sum_{i=1}^{n} r(x_i) \quad (3)$$

$$\overline{r(y)} = \frac{1}{n}\sum_{i=1}^{n} r(y_i) \quad (4)$$

The analysis shows that, while the qualitative trends among friction coefficient, electrical resistance, and metal release are consistent, their overall quantitative correlations are moderate rather than uniformly strong. This reflects the intrinsically multivariate nature of the friction process, in which no single parameter uniquely determines the system response. During the running-in stage (Stage I), mechanical wear dominates and the catalytic lubricating interface has not yet formed, leading to weak quantitative correlations. In the stable stage (Stage III), a mature lubricating interface is established and metal migration largely saturates, again resulting in weak coupling among the three signals. By contrast, in the self-adjusting stage (Stage II), where active metal migration and interfacial reconstruction jointly regulate the lubrication state, the correlations become pronounced, with relatively high Spearman coefficients (Supplementary Fig. 3).

We also note that temporal asynchrony further reduces apparent correlation strength. Changes in friction first trigger metal phase transitions and migration, which inherently require finite time, and the mass spectrometry detection of released metal introduces an additional delay of several seconds. These physical and instrumental lags are explicitly captured by the lagged correlation analysis.

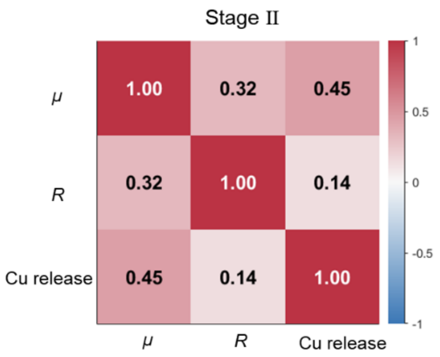

Supplementary Fig. 3| Correlation heatmap of friction coefficient (μ), electrical resistance (R) and Cu release during the self-adjusting stage (II).

Supplementary Note 2: Heat-induced Cu migration.

A static pressure test was performed to investigate whether mechanical force alone could drive Cu nanoparticle (NP) migration. A maximum Hertz contact stress of 1.34 GPa was applied (Supplementary Fig. 4a, b). Energy-dispersive X-ray spectroscopy (EDS) mapping of the contact area showed no significant Cu aggregation (Supplementary Figs. 4c, d). X-ray photoelectron spectroscopy (XPS) analysis further confirmed that the elemental content and chemical states of Cu and C remained unchanged before and after the test (Supplementary Figs. 4e, f). These results demonstrate that mechanical force alone is insufficient to drive Cu NP migration within the Cu/C film.

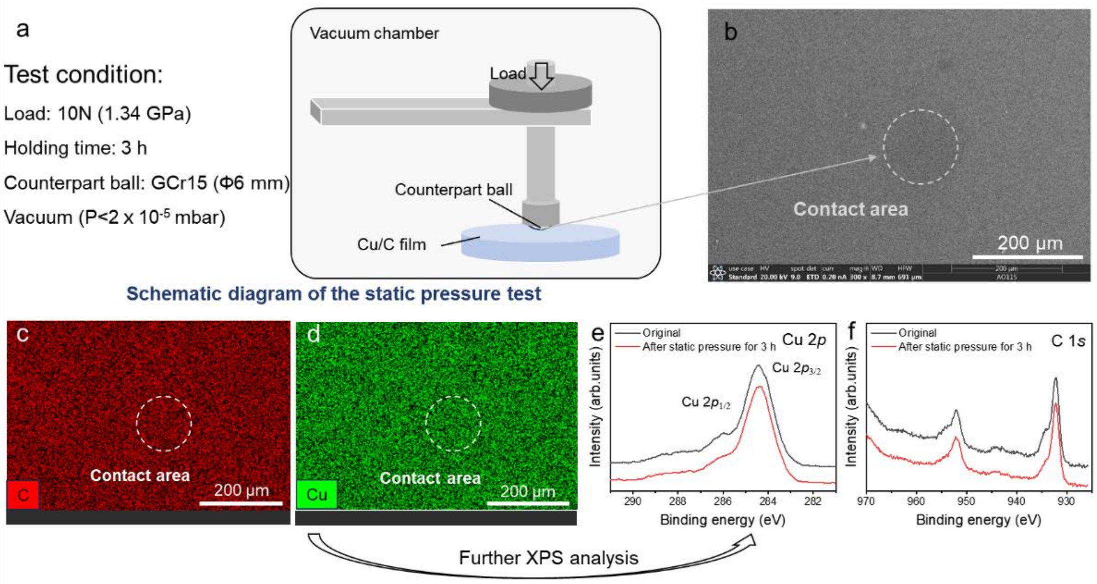

Supplementary Fig. 4| Static pressure test. (**a**) Schematic diagram and test conditions of the static pressure test. (**b**) FESEM image of the Cu/C film surface. (**c, d**) Element distribution mappings of the Cu and the C element, respectively. (**e**) XPS of Cu 2*p* before and after the static pressure test. (**f**) XPS of C 1*s* before and after the static pressure test.

The effect of temperature on Cu migration was investigated via vacuum annealing (<2×10^{-5} mbar for 3 h). Supplementary Figs. 5a-d show field-emission scanning electron microscopy (FESEM) images of the Cu/C film surface before and after annealing at 100, 200, and 300 °C. Nanopores inherent to the as-deposited film enlarge after annealing at 100 °C. At 200 °C, Cu NPs appear on the surface alongside reduced pore sizes. At 300 °C, a greater density of Cu NPs is observed, and the surface remains dense with only small pores; elemental point analysis confirms these NPs are predominantly Cu (Supplementary Fig. 5e). In-situ XPS shows the surface Cu/C atomic ratio increases steadily with temperature (Supplementary Fig. 5f), consistent with the annealing-induced morphological changes.

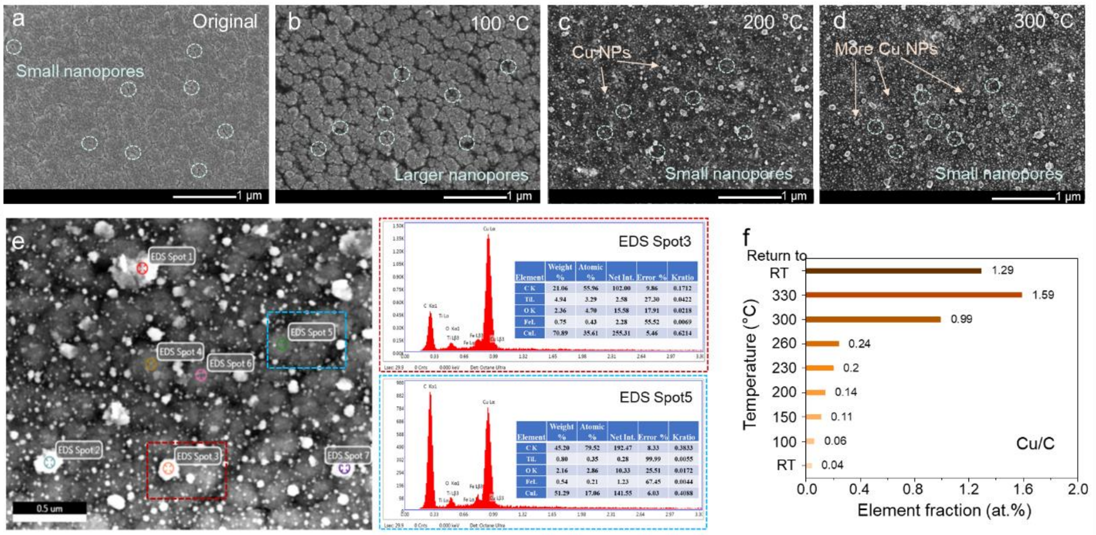

Supplementary Fig. 5| Heat-induce metal migration toward the film surface. (**a-d**) FESEM images of the Cu/C film surface before and after annealing at different temperature in vacuum. (**e**) Elemental point scanning of the Cu/C film surface after annealing at 300 °C in vacuum. (**f**) In-situ XPS analysis of the Cu/C ratios evolution on the film surface during heating.

Supplementary Fig. 6 shows a high-resolution transmission electron microscope (HRTEM) image of the microstructure of the Cu/C film. Cu nanocrystals with a size of 2–5 nm are uniformly dispersed in the amorphous carbon (a-C) matrix, and exhibit the characteristic (111), (200), (220), (311) and (400) crystal planes of Cu.

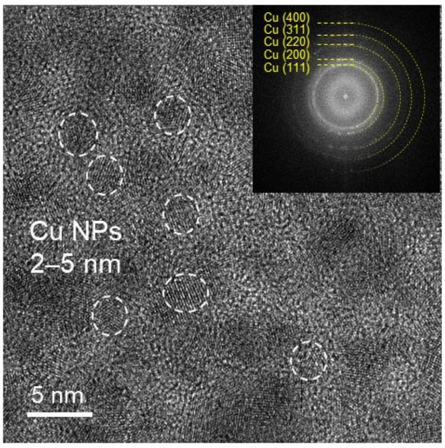

Supplementary Fig. 6| HRTEM image of the Cu/C film microstructure.

Supplementary Fig. 7 shows the in-situ TEM morphology of the Cu/C film microstructures during heating, where obvious structural changes are observed above 180 °C. As the temperature increases to 200 °C, some new Cu NPs gradually grow within the a-C matrix. With the temperature further rising to 300 °C, these newly grown Cu NPs gradually become larger and exhibit a spherical morphology.

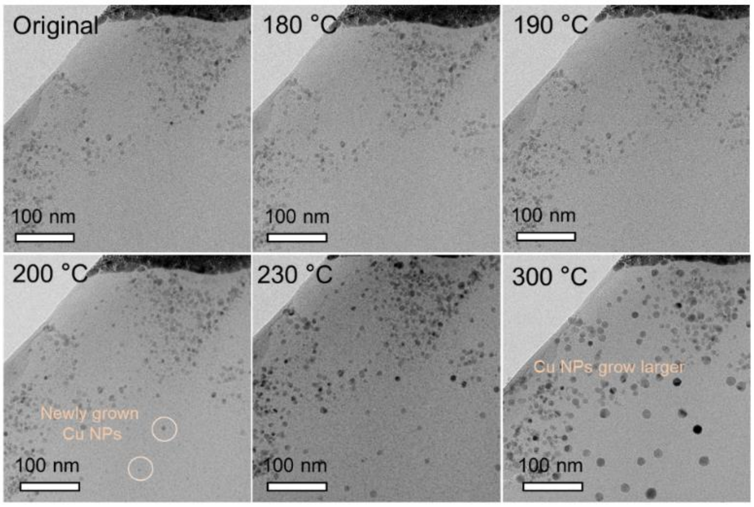

Supplementary Fig. 7| In-situ TEM images of the Cu/C film microstructures during heating.

Supplementary Note 3: The studies of Cu migration mechanism.

Supplementary Fig. 8 shows the in-situ TEM images of the Cu/C film during heating. Newly grown Cu NPs are observed near the surface at 300 °C, which is consistent with the results shown in Supplementary Fig. 7. When the temperature is raised to 400 °C, the Cu NPs close to the upper surface evaporate and gradually disappear.

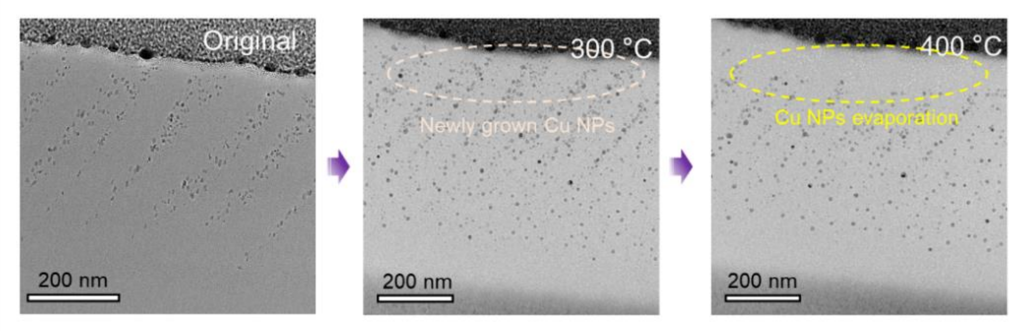

Supplementary Fig. 8| In-situ TEM images of Cu/C film during heating.

Supplementary Fig. 9 presents the Cu 2*p* XPS spectra of the Cu/C film measured before and after differential scanning calorimetry-thermogravimetric (DSC-TG) test. Before the DSC-TG test, an obvious Cu signal is observed. However, no Cu signal can be detected after the DSC-TG test. These results demonstrate the loss of Cu during the DSC-TG test. Combined with results in Supplementary Fig. 8, they indicate that an excessively high temperature causes Cu NPs to melt and evaporate, leading to the breakdown of their crystal structure.

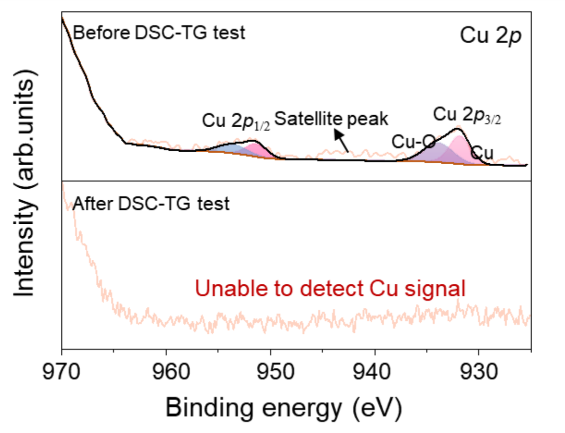

Supplementary Fig. 9| Cu 2*p* XPS spectra of the Cu/C film measured before and after the DSC-TG test.

To simulate Cu migration along nanopores, we constructed a quasi-conical pore geometry (Supplementary Fig. 10a) by introducing random atomic-scale roughness onto an ideal conical wall, closely mimicking realistic pore profiles. Molecular dynamics (MD) simulations using this model show the displacement of a Cu NP toward the surface over time (Supplementary Fig. 10b).

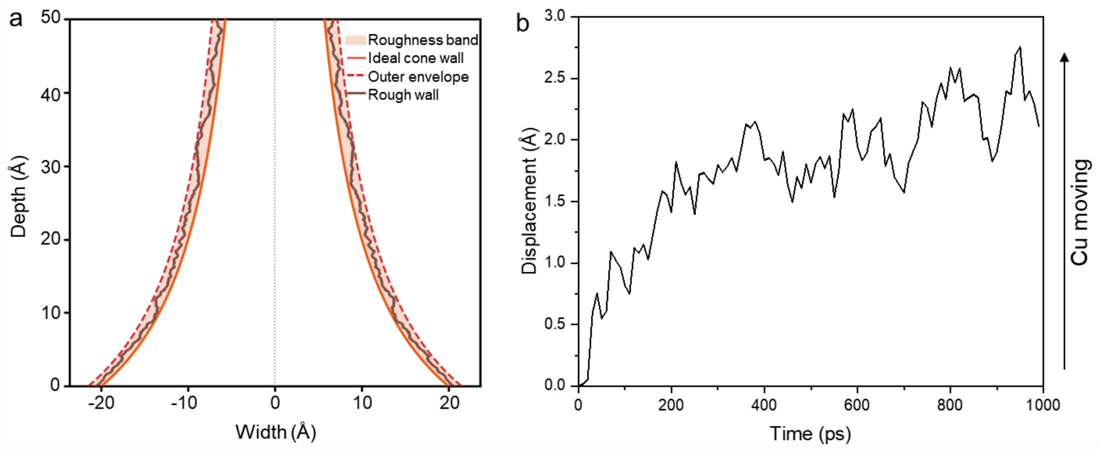

Supplementary Fig. 10| Construction of the nanopore model and MD simulation of Cu NP migration along the nanopore. (**a**) Schematic comparison between an ideal conical pore and a quasi-conical pore with randomly introduced surface roughness. (**b**) Time evolution of the displacement of a Cu NP in the quasi-conical pore from MD simulations.

Following annealing at 300 °C, which causes numerous Cu NPs to precipitate on the surface (Supplementary Fig. 5), the film's mechanical properties were evaluated. Neither the hardness nor the elastic modulus shows deterioration compared to the original film (Supplementary Fig. 11), indicating that the Cu/C film retains its mechanical integrity after Cu migration.

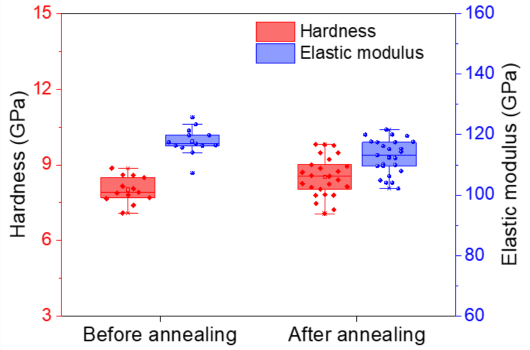

Supplementary Fig. 11| Hardness and elastic modulus of the Cu/C film before and after annealing at 300 °C in vacuum for 3 h.

Supplementary Note 4: The mechanism of ordered carbon formation catalyzed by Cu NPs.

Beyond the observation area in the main text (Fig. 3c, d), the transformation of a-C surrounding Cu NPs into ordered-carbon-encapsulated structures was also observed during heating, as shown in Supplementary Fig. 1 2.

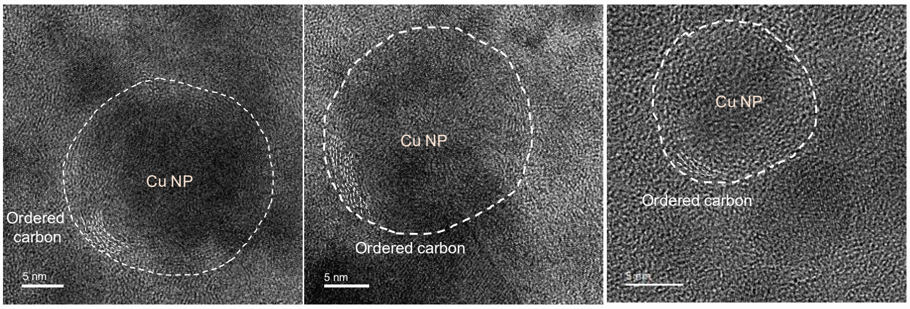

Supplementary Fig. 12| In-situ TEM images of ordered carbon at different regions of the Cu/C film during heating.

Supplementary Fig. 13 displays all atomic configurations along the reaction pathway from a-C to ordered carbon, calculated using the climbing-image nudged elastic band (CI-NEB) method. Frame 00 is the initial state, frame 12 is the final state, and intermediate frames show the transition. The process involves continuous bond breaking and reformation in the original carbon structure, culminating in a lower-energy ordered configuration.

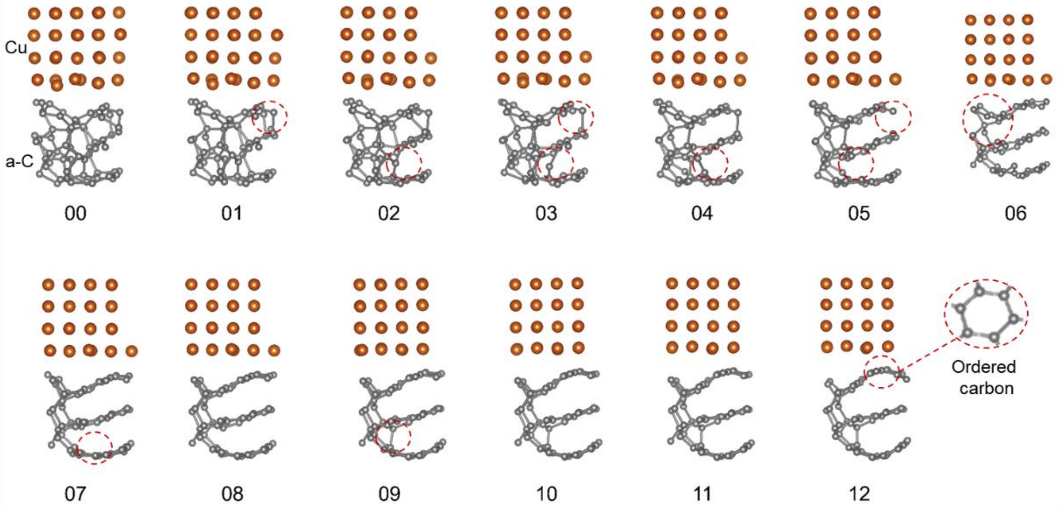

Supplementary Fig. 13| Atomic configurations along the reaction pathway obtained from the CI-NEB method.

Supplementary Fig. 14 shows the differential charge density (DCD) contours of the Cu–C configuration before the catalytic reaction. The pronounced charge redistribution indicates net electron transfer from Cu to C, which is consistent with the difference in their electronegativity.

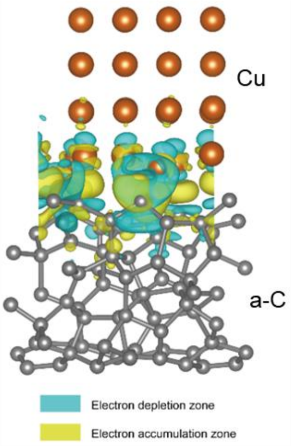

Supplementary Fig. 14| DCD contours of the Cu–C configuration before the catalytic reaction.

Density of states (DOS) comparisons further substantiate the evolution of the carbon electronic structure. Supplementary Fig. 15 shows that the DOS of pristine carbon has a pronounced peak near the Fermi level (E_f). This peak is significantly suppressed after Cu incorporation, accompanied by a redistribution of electronic states over a broader energy range, indicating that Cu–C interactions modify the local carbon electronic structure.

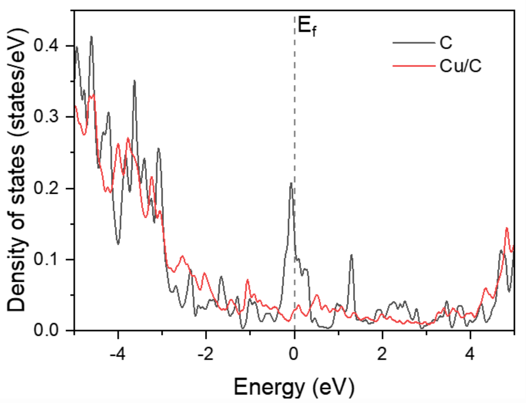

Supplementary Fig. 15| Density of states of C before and after Cu incorporation.

Supplementary Fig. 16 shows the negative crystal orbital Hamilton population (COHP) for the Cu–C bond. By convention, negative COHP values indicate bonding interactions, whereas positive values signify antibonding interactions. The integrated COHP (ICOHP), evaluated up to the Fermi level (E_f), provides a quantitative measure of bond strength. The presence of positive COHP states near the Fermi level indicates the formation of antibonding Cu–C interactions.

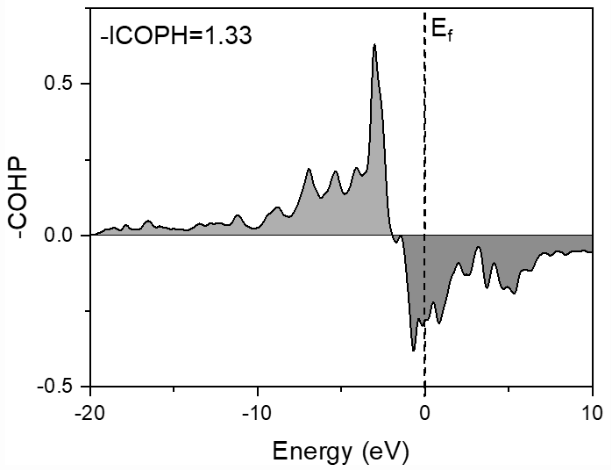

Supplementary Fig. 16| -COHP of the Cu–C bond.

To interrogate the electronic driving forces, we computed the work functions of Cu and a-C surfaces. The work function of Cu (4.62 eV) is substantially lower than that of a-C (6.05 eV), implying a greater propensity for electron donation from Cu.

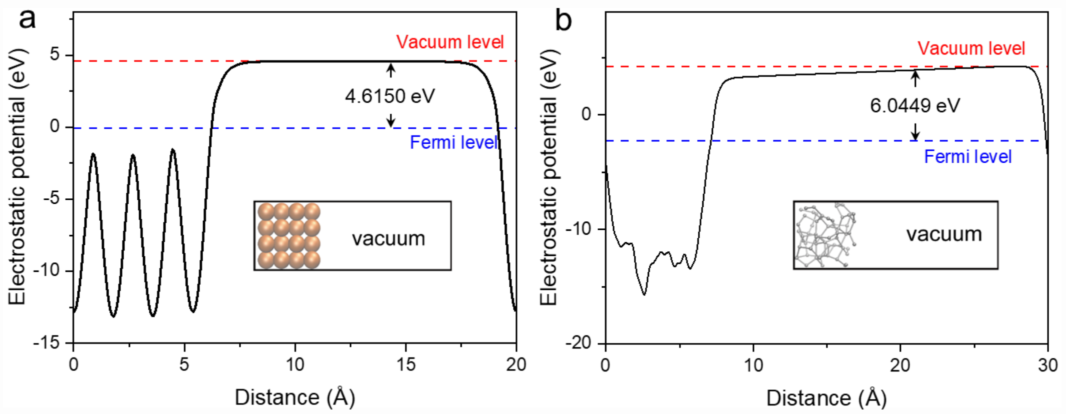

Supplementary Fig. 17| Work function calculations for the individual components. (**a**) Work function of the Cu system. (**b**) Work function of the a-C system.

Supplementary Note 5: Friction interfacial analysis of the Cu/C film.

The wear track depth of the Cu/C film and Cu consumption at different friction stages (after the running-in stage, after about 5000 m, and after over 10000 m) are shown in Supplementary Fig. 18. During the running-in stage (corresponding to a sliding distance of ~480.8 m), the Cu/C film suffers severe wear, with a considerable Cu consumption of about 2.46 at.%. The Cu consumption rate reaches 5.12×10^{-3} at.%/m, and the wear track depth is 166.7 nm. Subsequently, the friction system stabilizes, with the Cu content at around 22.95 at.% and the corresponding Cu consumption at 3.26 at.%, while the wear track depth further increases by only 49.2 nm after 5000 m of friction. When the sliding distance exceeds 10000 m, the Cu content decreases by a mere additional 0.41 at.%, reflecting a low Cu consumption rate of 3.77×10^{-4} at.%/m, while the wear track depth further increases by a further 64.8 nm. In summary, severe wear predominantly occurs during the running-in stage, whereas the wear decreases significantly in the stable friction stage. The Cu consumption in the Cu/C film remains very low over prolonged sliding, demonstrating that the Cu/C film can exhibit long-term low-friction performance.

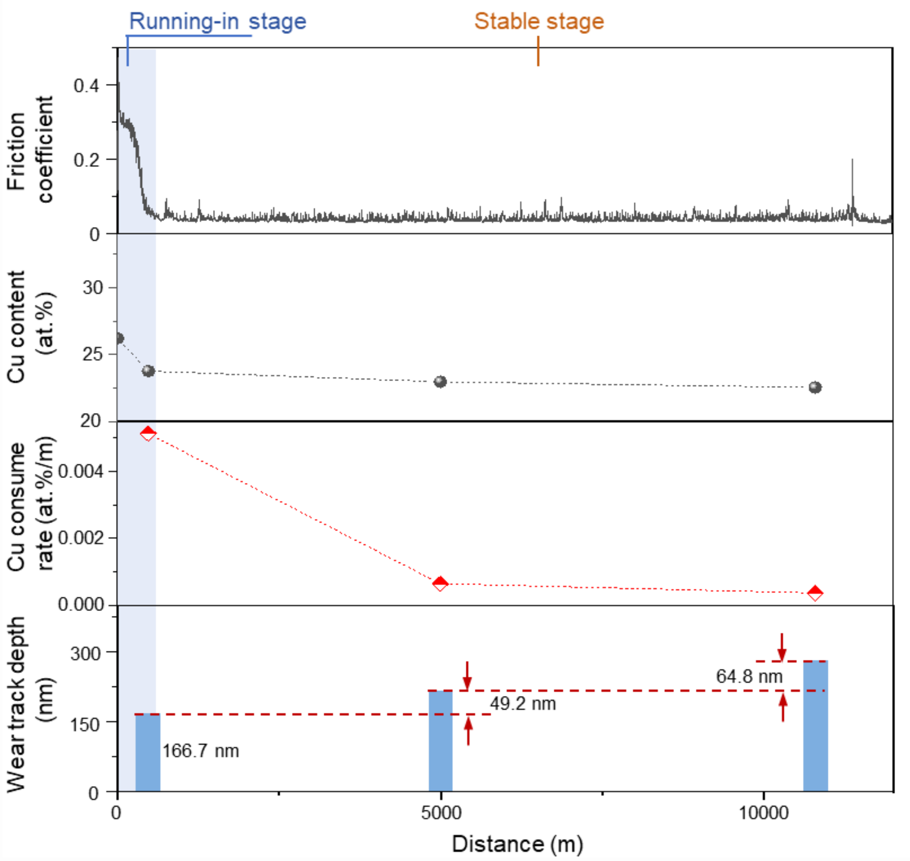

Supplementary Fig. 18| Friction coefficient curve, Cu content, Cu consume rate, and wear track depth of the Cu/C film at different friction stages.

The carbon structure changes of the Cu/C film at the friction interface can be reflected by Raman signal. The *D* and *G* peaks in Raman spectrum correspond to the breathing mode of the sp^2C atoms in the ring structure and the stretching of the sp^2C atoms in both ring and chain structures, respectively[2]. Here, the *D* and *G* peaks are fitted, and the ratio (I_D/I_G) of the areas of the *D* and *G* peaks is used to qualitatively characterize the structural change of the Cu/C film.

As shown in Supplementary Fig. 19, the I_D/I_G ratios fluctuate drastically during the running-in stage, indicating severe structural damage and an unstable friction interface. The ratios stabilize during the stable friction stage, signifying a robust interfacial structure.

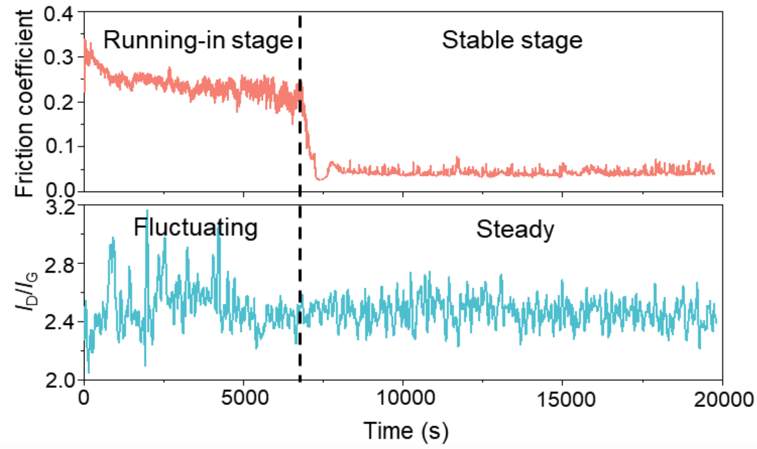

Supplementary Fig. 19| Friction coefficient curve and corresponding I_D/I_G ratios of the Cu/C film detected by in-situ Raman during friction in vacuum.

Supplementary Fig. 20 displays the microstructure and elemental composition of the wear scar, wear debris, and wear track of the Cu/C film after the long-duration friction test. The optical morphology and elemental distribution mapping of the wear scar (Supplementary Fig. 20a-d) indicate that Cu transfers to the counterpart ball. The HRTEM images of wear debris (Supplementary Fig. 20e-f) on the friction interface reveal the formation of numerous catalytically ordered carbon nanostructures. The cross-section TEM image and elemental distribution mapping of the wear track (Supplementary Fig. 20h-k) demonstrate that Cu migrates along the nanopores toward the friction interface and accumulates locally thereon.

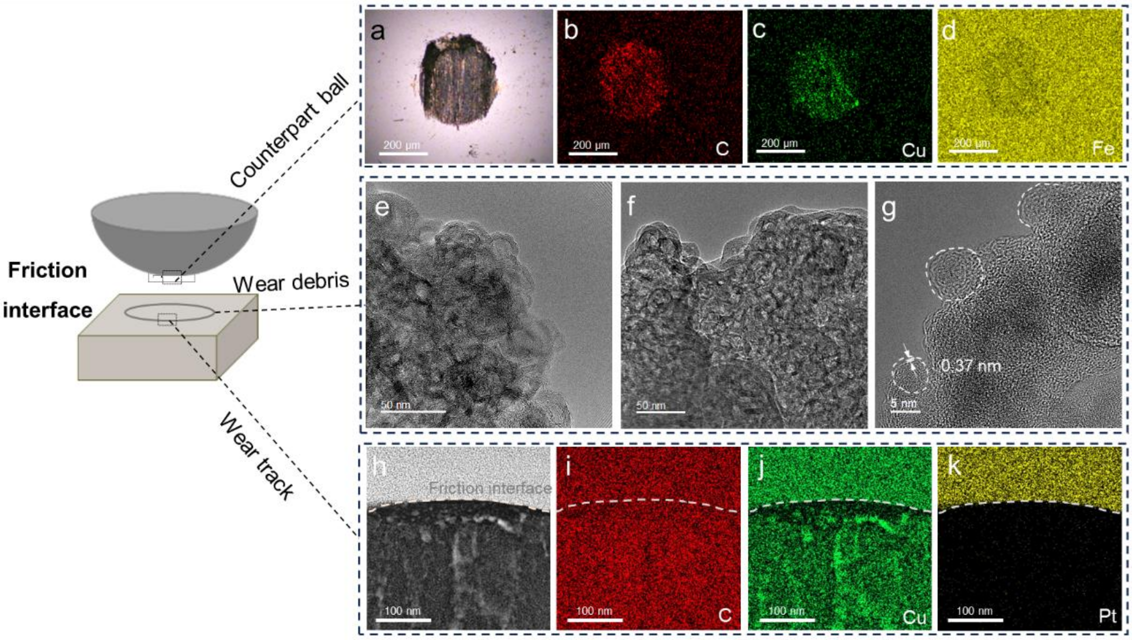

Supplementary Fig. 20| Interfacial analysis of the Cu/C film after long-duration test. (**a**) Optical morphology of wear scar. (**b-d**) Element distribution mapping of wear scar. (**e-g**) HRTEM images of wear debris. (**h-k**) TEM images and corresponding element distribution of cross-sections of the wear track.

Supplementary Note 6: The film preparation and information.

The Cu/C and C films were prepared using a closed-field unbalanced magnetron sputtering technique, with two graphite targets (99.99% purity), one Ti target (99.99% purity), and one Cu target (99.99% purity), as presented in Supplementary Fig. 21a. Prior to deposition, surface contaminations on the substrates were etched away by Ar^+ plasma with a pulsed bias of -400 V for 40 min. The Ti and TiC interlayers were then deposited to enhance the adhesion between the films and substrates. The holder rotation speed was set to 5 r/min. The C and Cu/C films were prepared separately by adjusting the Cu sputtering current (0A and 0.50–0.75 A) and graphite sputtering current (6 A). Ar gas was continuously supplied as the sputtering gas at a flow of 30 sccm throughout the deposition process. During the deposition of the Cu/C film, the bias voltage was set to -70 V. Supplementary Fig. 21b, c shows FESEM images of the surface and cross-sectional morphologies of the C and Cu/C films (the Cu content is about 26.21 at.%; Supplementary Fig. 18). Both the C and Cu/C films are dense, and the thicknesses of the C and Cu/C films are 2.76 μm and 3.12 μm, respectively.

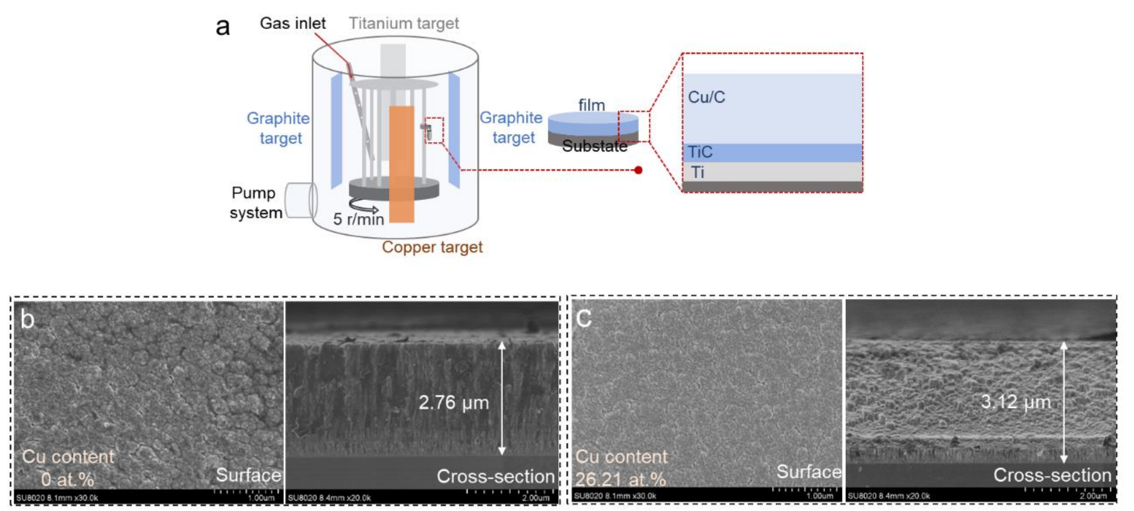

Supplementary Fig. 21| Schematic diagram of the film deposition device and information about the Cu/C film. (a) Schematic diagram of the magnetron sputtering device. **(b, c)** FESEM images of surface and cross morphology of the C film and the Cu/C film.

The hardness values of the Cu/C and C films are 8.01 GPa and 10.36 GPa, respectively. For adhesion strength, the scratch test shows that only minor traces appear on the Cu/C film at a load of 44.28 N, a value that corresponds to the film-substrate adhesion[3]. This indicates that the Cu/C film possesses high adhesive strength to the substrates, as illustrated in Supplementary Fig. 22.

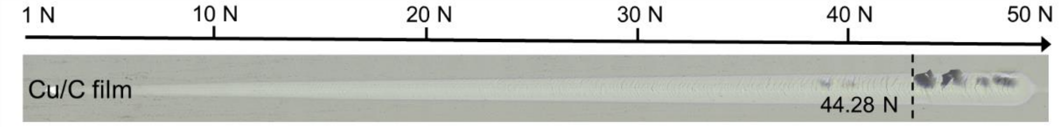

Supplementary Fig. 22| Scratch morphology of the Cu/C film.

The Raman spectra of the C and Cu/C films are shown in Supplementary Fig. 23a. Both the C and Cu/C films display characteristic *D* and *G* peaks, with their I_D/I_G ratios being 2.795 and 3.119 respectively. The X-ray diffraction (XRD) patterns of the C and Cu/C films are depicted in Supplementary Fig. 23b. The Cu diffraction peak is broad and weak, indicating that Cu exists in the a-C matrix as small-sizes nanocrystals. The XPS spectra of C 1*s*, O 1*s*, Cu 2*p*, and Cu *LMM*

of the Cu/C film are shown in Supplementary Fig. 23c-f. Cu in the Cu/C film is present as metallic Cu and Cu_2O, and no obvious chemical bonding is detected between Cu and C. Combined with the result in Supplementary Fig. 6, the localized oxidation of Cu NPs on the surface might be attributed to exposure to atmospheric environment for some time before the XPS test.

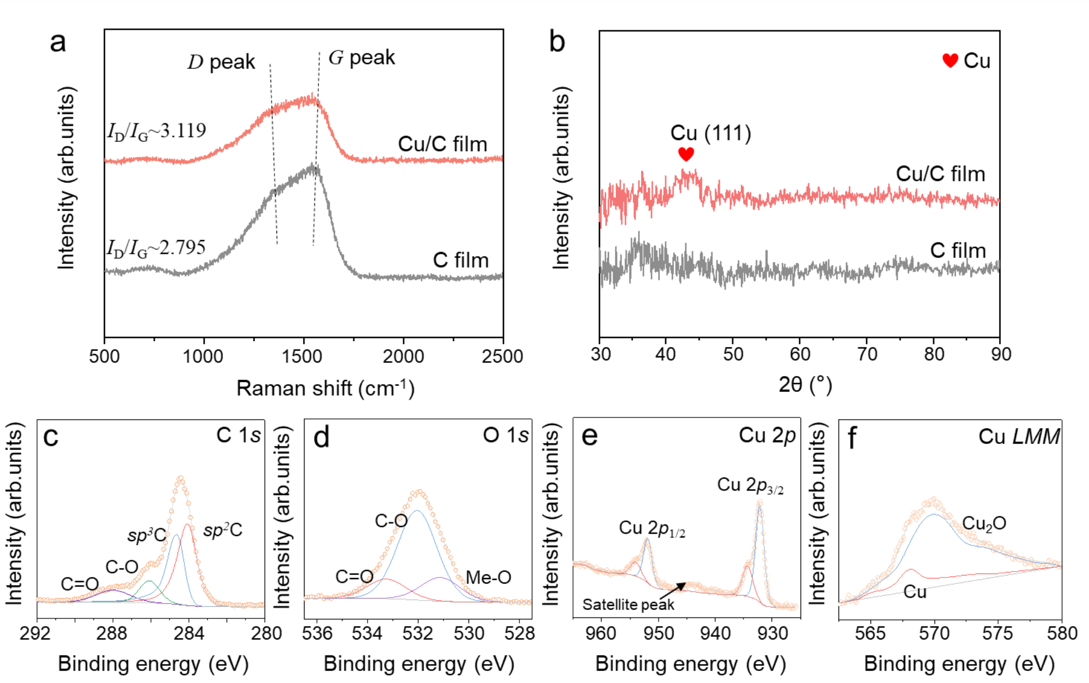

Supplementary Fig. 23| The original structure of the Cu/C film and C film. (**a**) Raman spectra. (**b**) XRD patterns. (**c-f**) XPS of the Cu/C film.

Supplementary Note 7: Development of the NEP model and set-up for MD simulations.

As schematically shown in Supplementary Fig. 24, the Cu–C training dataset construction and neuroevolution potential (NEP) training process began with an initial C dataset adapted from Wang *et al*.[4]. This was combined with newly generated reference structures for pure Cu and Cu–C systems, including bulk Cu, Cu clusters, and various Cu–C configurations with different compositions and surface models to cover relevant atomic environments.

Initial configurations underwent ab initio molecular dynamics (AIMD) to generate diverse structures, which were labeled by single-point density functional theory (DFT) calculations using the Vienna Ab-initio Simulation Package (VASP) [5,6]. The DFT-labeled configurations were split into training and test sets to train the NEP model. An active-learning strategy was employed, iteratively optimizing the training by evaluating against a target accuracy. If the criterion was unmet, additional configurations were added to the dataset, and training repeated until convergence. The final converged NEP model was used for all MD simulations.

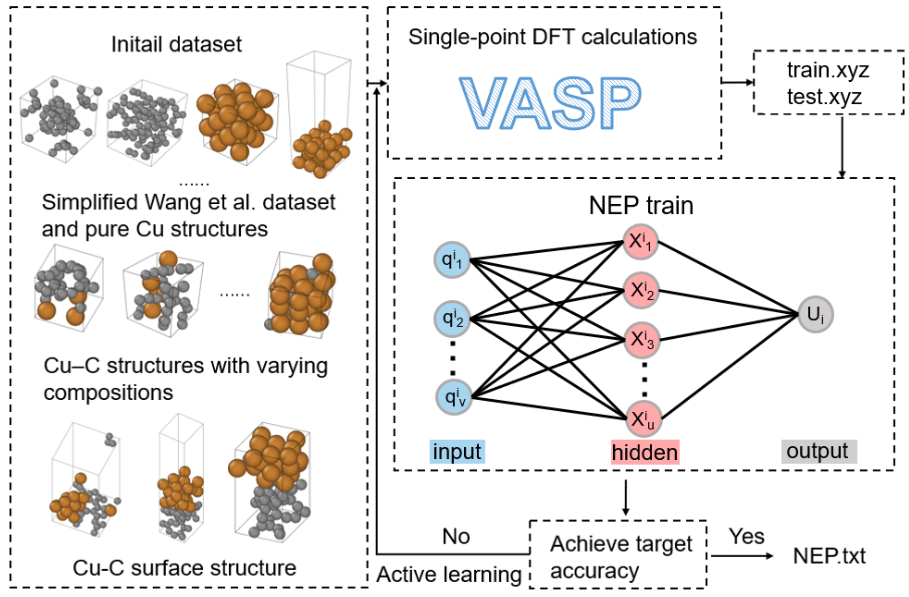

Supplementary Fig. 24| Schematic overview of the dataset construction and NEP training procedure.

The trained Cu–C NEP model exhibits excellent overall performance in predicting energy, force and virial, with the test errors of RMSEe = 11.173 meV/atom, RMSEf = 0.306 eV/Å, and RMSEv = 49.231 meV/atom, respectively (Supplementary Fig. 25).

To further validate this force field, an a-C model for migration simulations was constructed using a melt-quench procedure. A diamond 8×8×20 supercell (10240 atoms) was heated to 9000 K in the NVT ensemble for 400 ps to form a liquid. This liquid was cooled to 5000 K and equilibrated for 200 ps. A rapid quench from 5000 K to 1000 K at 10^{12} K/s produced a disordered a-C sample. Finally, the system was quenched from 1000 K to 300 K over 200 ps and equilibrated at 300 K for 500 ps to eliminate metastable configurations and internal stress. The radial distribution function (RDF) from NEP-based MD simulations (Supplementary Fig. 26) compares well with experimental data[4], showing characteristic peaks at ~1.42 Å and ~2.46 Å corresponding to short-range order of sp^2-bonded carbon, confirming the presence of graphitic-like domains.

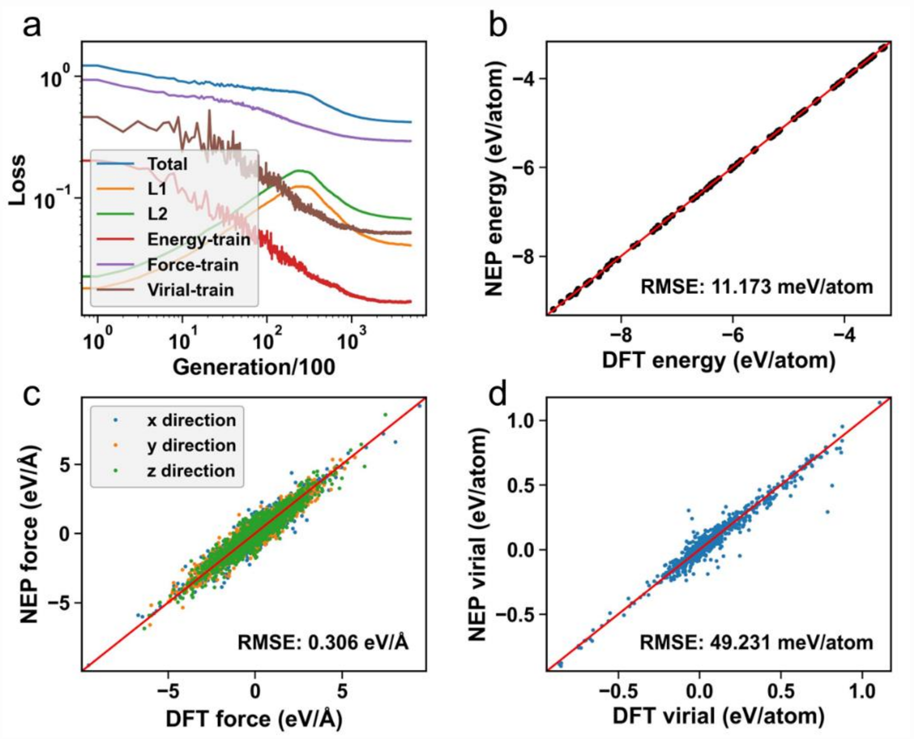

Supplementary Fig. 25| Trained NEP model. (**a**) Evolution of RMSEs of energy (eV/atom), force (eV/Å) and virial (eV/atom) as a function of training iterations for the training set. (**b-d**) Parity plots of energy, force, and virial comparing NEP predictions with DFT-PBE reference data for the test set.

Supplementary Table. 1| Hyperparameters for NEP training.

version	4	lambda_1	0.05
type	2 Cu C	lambda_2	0.05
zbl	2.0	lambda_e	1.0
cutoff	6 4	lambda_f	1.0
n_max	8 8	lambda_v	0.1
basis_size	12 12	batch	1000
l_max	4 2	population	50
neuron	50	generation	500000

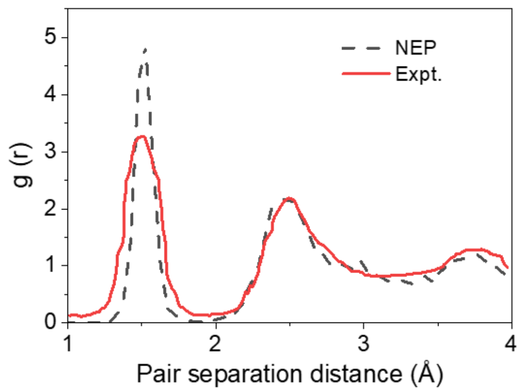

Supplementary Fig. 26| The RDF curve of the a-C sample from NEP-based MD simulations (dashed line) and experiment measurements (solid line).

Supplementary References

1 Spearman C. The proof and measurement of association between two things. *The American Journal of Psychology* **100**, 441-71 (1904).
2 Ferrari, A. C. & Robertson, J. Interpretation of Raman spectra of disordered and amorphous carbon. *Physical review B* **61**, 14095 (2000).
3 Zaidi, H., Djamai, A., Chin, K. J. & Mathia, T. Characterisation of DLC coating adherence by scratch testing. *Tribology International* **39**, 124-128 (2006).
4 Wang, Y., Fan, Z., Qian, P., Caro, M. A. & Ala-Nissila, T. Density dependence of thermal conductivity in nanoporous and amorphous carbon with machine-learned molecular dynamics. *Physical Review B* **111,** 094205 (2025).
5 Kresse, G. & Furthmüller, J. Efficient iterative schemes for ab initio total-energy calculations using a plane-wave basis set. *Physical Review B* **54**, 11169-11186 (1996).
6 Kresse, G. & Joubert, D. From ultrasoft pseudopotentials to the projector augmented-wave method. *Physical Review B* **59**, 1758-1775 (1999).
