# Supplementary material for: An intelligent feedback loop for sustaining self-lubrication and wear resistance
Source: Nat Commun. 2026 Jun 2;17:7071. doi: 10.1038/s41467-026-73957-6 (PMC13392075; doi:10.1038/s41467-026-73957-6)
Supplement: Supplementary file 2 — Description of Additional Supplementary File [file 41467_2026_73957_MOESM2_ESM.pdf]

The Description of Additional Supplementary Files

Supplementary Movie 1 is the molecular dynamics simulation of Cu melting and migrating along a nanopore under heat.

Supplementary Movie 2 is the in-situ TEM observation of ordered carbon formation catalyzed by Cu during heating.

Supplementary Movie 3 is the molecular dynamics simulation of ordered carbon formation catalyzed by Cu.
