# Supplementary material for: An intelligent feedback loop for sustaining self-lubrication and wear resistance
Source: Nat Commun. 2026 Jun 2;17:7071. doi: 10.1038/s41467-026-73957-6 (PMC13392075; doi:10.1038/s41467-026-73957-6)
Supplement: Supplementary file 7 — Transparent Peer Review file [file 41467_2026_73957_MOESM7_ESM.pdf]

nature portfolio

Peer Review File

An intelligent feedback loop for sustaining self-lubrication and wear resistance

Corresponding Author: Dr Li Ji

This file contains all reviewer reports in order by version, followed by all author rebuttals in order by version.

Version 0:

Reviewer comments:

Reviewer #1

(Remarks to the Author)
The author combined experimental and simulation methods to reveal the self-adjusting lubricating properties of Cu(Au)/C nanocomposite films. They point out that the Cu(Au)/C nanocomposite films have excellent lubricating performance due to the friction-induced migration of Cu(Au) metal nanoparticles along nano-pores to the friction interface, which catalyzes the in-situ formation of ordered carbon nano-structures. The film achieves robust lubrication with an ultra-long wear life in vacuum. Their idea to design a frictional film with self-sensing, self-adjusting, and self-repairing properties is quite interesting and novel. The author needs to address several issues before considering publication by Nature Communications.
1. The author claims that the Cu(Au)/C nanocomposite films have excellent lubricating performance. However, the characterization and film preparation are only related to the Cu/C film. It isn't very clear whether the author used only one kind of metal or a Cu-Au mixture metal to combine with the carbon to form the nanocomposite films.
2. Continue with the former comment. The author also needs to clarify how the characterization and simulation of the Cu/C film support the function of Au in the Au/C film.
3. One of the key mechanisms that enables the Cu(Au)/C nanocomposite films to exhibit excellent lubricating performance is that the NPs can migrate to the surface under thermal conditions. However, Figs. 2a, b, d; Supplementary Figs. 10 and 11 are not clear enough to show this migrant trend. Supplementary Video 1 is too shaky, with something moving but not clearly visible. Additionally, those distinctive particles appear to be growing in size with increasing temperature but not moving. The author needs to clearly mark and show the migration of NPs to support their hypothesis.
4. Fig. S4a exhibits period noise. What is the reason?
5. The author claims that metal migration in the film can be precisely regulated in response to frictional heat, enabling intelligent lubrication. According to Supplementary Note 1, it appears that the film can only work within a specific range of sliding speeds. For high speed, the film wears out very quickly. The reasons for the film's failure at high speed, as well as its limitations in adjusting ability, should be discussed in the manuscript.
6. The author claims that the Cu/C film shows robust lubrication with an ultra-long wear life, 147 times and 38 times longer than that of hydrogen-free C films and hydrogen-containing C films, respectively. The experimental details and results of hydrogen-free C films and hydrogen-containing C films should be provided, particularly the wear rate.

Reviewer #2

(Remarks to the Author)
The authors discovered a novel phenomenon of metal migration and catalysis to achieve intelligent lubrication in Cu or Au doped amorphous carbon-based films, significantly extending their lifespan in vacuum. Furthermore, the dynamic metal migration and catalytic mechanisms were revealed through in situ HRTEM observations in combined with simulation calculations. This study experimentally and visually demonstrates the bioinspired intelligent lubrication behavior of the Cu(Au)/C films, which is of great interest and offers a new avenue to design intelligent materials. Some points require the authors' attention before publication.
1.This work claimed that the anti-wear life of amorphous carbon films in vacuum environments was extended through intelligent lubrication of Cu(Au)/C films. How does this performance as compared with other similar materials at present?
2.The authors found that the melting temperature of Cu nanoparticles varies with size by in situ observations, which maybe a good method for detecting the melting temperature of nanomaterials with different sizes. It is suggested to supplement the

corresponding discussion in the manuscript.
3.The migration of Cu within the amorphous carbon matrices affects the film microstructure. How about the variations in mechanical properties of the Cu doped amorphous carbon films with the above-mentioned migration?
4.The authors considered that thermal effect induces the migration of doped metals. In fact, forces can also cause lattice distortion and atomic motion. Explain whether the effect of force is alone considered or not?
5.The authors qualitatively analyzed the changes in bonding states and interfacial electronic properties through COHP and work function calculations. If possible, adding density of states (DOS) comparisons before and after Cu incorporation would further substantiate the electronic structure evolution of carbon.
6.Some film parameters, such as thickness and mechanical properties, are important for their applications. What about the thickness, hardness, and adhesion strength of the films used in this study?
7.The elemental composition of the steel substrate should be provided.
8.The authors constructed various configurations, including bulk-mixed and interfacial models, to capture the key states of Cu–C interactions and developed a NEP potential to describe the corresponding interatomic forces. How reliable is the developed NEP potential?
9.Some details need to be verified, for example
a)It seems that the images in Fig. 3 are out of order.
b)Check "Cu(" and "C(" in S2. If it is a special symbol, please provide an annotation and explanation.
c)Ensure consistent capitalization formatting for titles in the references.
d)It is mentioned “The depth of wear tracks was measured via the three-dimensional white light interferometry (UP-Lambda, Rtec-Instruments, America) and further calculated the wear rates”. Grammatical revision is needed for this sentence.

Reviewer #3

(Remarks to the Author)
This paper proposes and validates a class of biomimetic intelligent self-regulating lubricating nanocomposite films. By harnessing heat generated within friction pairs to activate phase transitions, self-migration, and catalytic behavior in metallic nanoparticles, it establishes a closed-loop self-regulating lubrication system. Comprehensive in-situ characterization and multi-scale simulations provide robust theoretical and experimental evidence. However, several questions are still required to further clarify.
1)Regarding the stability of metal supply during prolonged cycling, whilst the authors demonstrate the sustainability of the metal reservoir, the paper lacks quantitative metrics (total metal content, consumption rate, etc.). Quantitative analysis should be supplemented.
2)The catalytic formation of ordered carbon structures is substantiated solely by local TEM images, lacking statistical data to confirm its prevalence.
3)The rationale for selecting nonporous structures in the model warrants further discussion. While MD simulations employed a single conical pore configuration, actual thin-film pore distributions exhibit greater complexity.
4)The role of Au is not sufficiently elaborated. Although the paper refers to Cu (Au)/C, the results predominantly feature Cu/C, with Au only marginally addressed in transfer film observations.
5)The process annotations in Figure 1b (states I–VI) require enhanced explanation, clearly elucidating the physical significance of each stage.
6)Some TEM and IFFT images in Figure 2 are insufficiently clear. It is recommended to enhance contrast or annotate key crystal planes.
7)The energy barrier diagram in Figure 3 would be more readable if molecular structures representing reaction pathways were incorporated.
8)Unit formats throughout the paper need standardization.
9)The language is required to further refine its clarity and readability.

Reviewer #4

(Remarks to the Author)
The manuscript reports Cu(Au)/C nanocomposite films designed as bioinspired lubricants with self-adjusting and self-repairing capabilities under friction. The authors integrate in situ TEM, Raman spectroscopy, DSC-TG, electrical measurements, tribological testing, MD simulations, and DFT calculations to establish a mechanistic framework in which friction-induced heat triggers metal nanoparticle melting, migration, and catalytically driven ordering of carbon nanostructures.

Although the concept of in situ tribofilm formation and self-lubrication has been explored previously (Nature 536, 67–71 (2016); Carbon 138, 61–68 (2018); Mater. Today Chem. 26, 101112 (2022); ACS Appl. Mater. Interfaces 15, 30070–30082 (2023)), the depth of investigation, mechanistic correlation, and tribological performance demonstrated in this work are impressive. The study is comprehensive and experimentally rigorous, offering detailed mechanistic insights that are potentially valuable to the field of tribology and related disciplines.

However, several scientific claims require solid supporting evidence and/or clearer presentation before the manuscript can be considered for publication. A major revision is required before the manuscript can be considered for potential acceptance

1. The central concept that friction-induced solid-liquid transition of metal nanoparticles enables autonomous replenishment

and catalytic ordering of carbon is compelling and represents a distinctive mechanistic pathway for self-regulated lubrication. However, the manuscript overstates “bio-inspired intelligence,” which is essentially an intelligent feedback loop driven by frictional heat rather than a physical mimicry of any biological structure. Moreover, the concepts of self-sensing and self-regulation are not unique to biomimetic systems; they are recognised across engineering and materials science without necessarily implying biological inspiration.

2. The proposed feedback cycle relies on the tight coupling among friction coefficient, resistance, metal release, and interfacial structure. While qualitative trends are shown, quantitative correlations are limited. It is recommended to provide cross-correlation analysis to confirm causality rather than coincidence.

3. The dominant results and discussion focus on the Cu/C nanocomposite, while only Fig. 1(b) and Supplementary Figs. 5 and 6 pertain to the Au/C system. A comparison between the Cu/C and Au/C systems should be provided, including their tribological performance and the corresponding mechanistic framework, to clearly distinguish the roles of Au and Cu. This is particularly important because Au exhibits a relatively low melting temperature at the nanoscale, stronger catalytic activity, and significantly higher cost, all of which influence the lubrication mechanism and practical relevance.

4. The proposed intelligent Cu/C nanocomposite demonstrates impressive tribological performance over the 40 km test. However, material characterisation of both the ball and the wear track of the nanocomposite after this long-duration test is missing. This analysis is essential to confirm Cu migration to the near-surface region of the composite film, the catalytic ordering of carbon within the worn track, and the presence of Cu transfer films on the ball. That information is essential to further support the proposed mechanistic framework for long-duration test.

5. It is unclear why the high-frequency reciprocating tests (6 Hz ≈ 9.4 cm/s and 9 Hz ≈ 14.2 cm/s) show significant different performance compared with the rotating test conducted at a higher linear speed (25.7 cm/s). Although the shear directions differ, rotation imposes a unidirectional shear while reciprocation alternates the sliding direction, the discrepancy in tribological behaviour is not fully explained. Specifically, it is puzzling why the high-frequency reciprocating tests, which still operate at significantly lower linear speeds than the rotating test, result in high friction and failure.

6. It is recommended to thoroughly improve the grammar and clarity of the descriptions in the supplementary document. For example, the statements ‘Supplementary Fig. 2–4 depicts the relationship between friction coefficient (μ) and R at different friction frequencies is shown in’ and ‘From Supplementary Fig. 2 and 3, when the friction interface is in a metal-lack state (run-in state), during which the R values are relatively high’ are grammatically incorrect and should be revised.

7. The information about how the amorphous and graphitic-like structures in Fig. 3f and 3i are generated is missing. It is unclear if that structure is generated from an MD simulation. The definition of reaction coordination should be clearly defined.

8. The DFT-COHP analysis indicates C-C weakening in the presence of Cu. However, the calculation is based on a target C-C bond. This practice lowers the reliability of the calculation. It is recommended to provide the calculation that averages all of the C-C bonds or C-C bonds near the interface.

9. The reported material in the experiment is a Cu(Au)/C film; however, the simulation considers only a Cu/C system. Because Au nanoparticles are excellent catalysts that could significantly influence tribofilm formation, the authors need to provide a clear justification for their model selection (i.e., why only Cu was included) and explain the expected behaviour and contribution of Au.

10. The potential used in this work is adapted from Wang et al. [57], which primarily describes C-C interactions. Because this study focuses on the influence of Cu, an adequate and dedicated Cu-C dataset is needed to ensure that the potential can reliably capture Cu-C interactions.

The authors stated that “several representative Cu-C structures with varying compositional ratios were selected” and that “Cu-C surface models” were included. However, this description is highly generic and insufficient. A detailed description of the Cu-C training dataset is needed.

Importantly, for the simulations presented in the manuscript, (i) the migration of melted Cu nanoparticles within a conical pore in the a-C model, and (ii) Cu-nanoparticle-induced catalytic behaviour, it is not clear whether these specific atomic environments were explicitly included in the training set.

11. The key behaviors examined in the MD simulation section rely on nanometer-scale Cu nanoparticles, whose properties differ substantially from bulk Cu. However, it remains unclear how the training dataset reflects the distinct physicochemical characteristics of nanosized Cu as opposed to bulk Cu.

The manuscript indicates that only Cu-C related structures were included in the training set, with no dedicated dataset for isolated nanoscale Cu clusters. It is necessary to validate if the developed/trained potential can indeed reproduce nanosized-Cu properties in MD simulation, such as the reduced melting point.

Version 1:

Reviewer comments:

Reviewer #1

(Remarks to the Author)
The author gave a reasonable response to my comments. The manuscript is now more solid and clear, and meets the journal's quality requirements. I have no further comments.

Reviewer #2

(Remarks to the Author)
The authors have addressed all my concerns in the revised version,and I believe that it is now acceptable for publication in the Nature Communications in the present form.

Reviewer #3

(Remarks to the Author)
The manuscript has been revised in the light of comments, which could be accepted as its current state.

Reviewer #5

(Remarks to the Author)
I evaluated the manuscript revisions and author responses to Reviewer #4's comments 7 to 11 regarding the simulation work. I believe all concerns have been fully addressed.

Open Access This Peer Review File is licensed under a Creative Commons Attribution 4.0 International License, which permits use, sharing, adaptation, distribution and reproduction in any medium or format, as long as you give appropriate credit to the original author(s) and the source, provide a link to the Creative Commons license, and indicate if changes were made.
In cases where reviewers are anonymous, credit should be given to 'Anonymous Referee' and the source.
The images or other third party material in this Peer Review File are included in the article’s Creative Commons license, unless indicated otherwise in a credit line to the material. If material is not included in the article’s Creative Commons license and your intended use is not permitted by statutory regulation or exceeds the permitted use, you will need to obtain permission directly from the copyright holder.
To view a copy of this license, visit https://creativecommons.org/licenses/by/4.0/

Response to the Reviewers' comments

Manuscript ID: NCOMMS-25-79557

First, we would like to thank the Reviewers' works and the detailed comments to improve our manuscript. They are meaningful and useful to promote our manuscript to be a more qualified one. Besides the **Revised manuscript**, we also submit a copy as the supporting information (as **Revised manuscript 2**), in which the revised parts were highlighted in red color.

Reviewer: #1 (Remarks to the Author)

The author combined experimental and simulation methods to reveal the self-adjusting lubricating properties of Cu(Au)/C nanocomposite films. They point out that the Cu(Au)/C nanocomposite films have excellent lubricating performance due to the friction-induced migration of Cu(Au) metal nanoparticles along nano-pores to the friction interface, which catalyzes the in-situ formation of ordered carbon nano-structures. The film achieves robust lubrication with an ultra-long wear life in vacuum. Their idea to design a frictional film with self-sensing, self-adjusting, and self-repairing properties is quite interesting and novel. The author needs to address several issues before considering publication by Nature Communications.

Response: We sincerely thank the Reviewer for their thoughtful evaluation and valuable suggestions, which have helped us significantly strengthen the manuscript. We are encouraged by their positive assessment of the novelty and interest of our work. We have carefully addressed each of the points raised, and we hope our explanations and revisions in the manuscript provide satisfactory clarification.

1. The author claims that the Cu(Au)/C nanocomposite films have excellent lubricating performance. However, the characterization and film preparation are only related to the Cu/C film. It isn't very clear whether the author used only one kind of metal or a Cu-Au mixture metal to combine with the carbon to form the nanocomposite films.

Author reply: We thank the Reviewer for raising this important point regarding the clarity of the film composition, and we apologize for the ambiguity in the original presentation. In the study, two distinct systems were investigated: a Cu-doped carbon (Cu/C) film and an Au-doped carbon (Au/C) film. Although both systems exhibit qualitatively similar metal migration behavior and tribological trends, we agree that intermixing results from the two films was not sufficiently rigorous, as material-specific differences may influence the detailed mechanisms.

In direct response to this comment, we have undertaken a substantial revision to improve clarity and internal consistency. We have now assembled a complete, self-consistent dataset focused exclusively on the Cu/C film, including in-situ TEM characterization, comprehensive tribological

measurements, and supporting theoretical simulations. This comprehensive evidence fully substantiates the proposed intelligent lubrication framework.

Accordingly, in the Revised manuscript, all data previously derived from the Au/C system have been replaced with corresponding results from the Cu/C film. The revised narrative now focuses unambiguously on elucidating the intelligent lubrication behavior and underlying mechanism in the Cu/C system.

The principal data modifications in the Revised manuscript are summarized as follows:

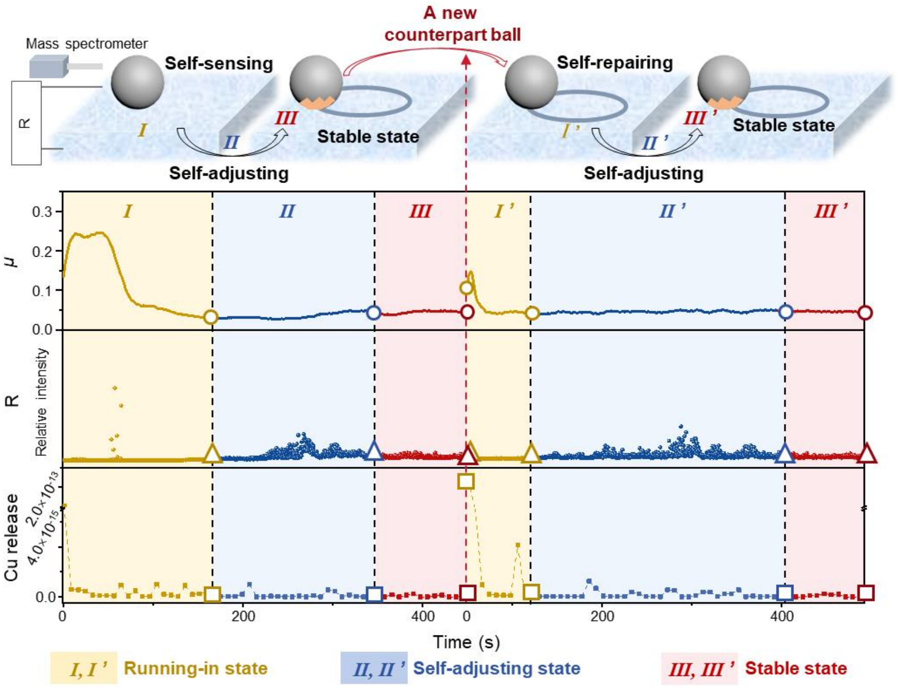

Fig. 1b. Real-time monitoring results of μ, electrical resistance (R) and Cu release during two consecutive friction processes of the Cu/C film.

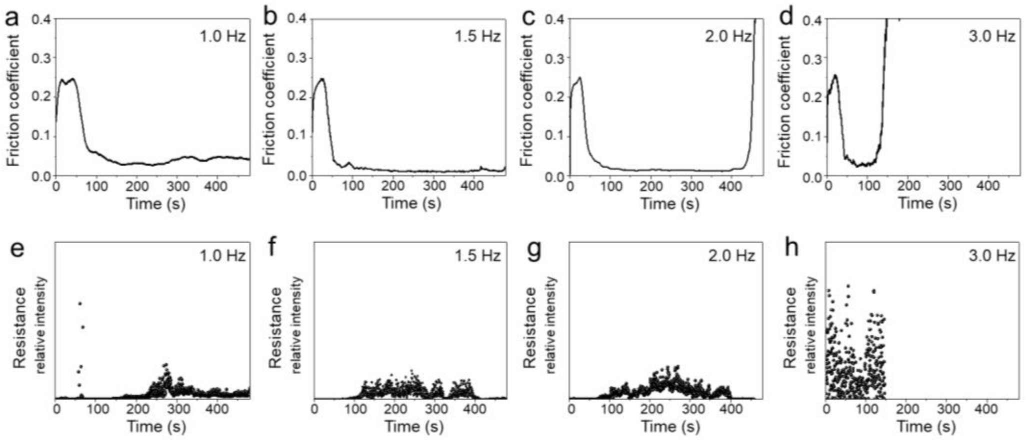

Supplementary Fig. 2. Friction coefficient curves and corresponding electrical resistance of the Cu/C film at different reciprocating frequencies. (a, e) 1.0 Hz. (b, f) 1.5 Hz, (c, g) 2.0 Hz, (d, h) 3.0 Hz.

2. Continue with the former comment. The author also needs to clarify how the characterization and simulation of the Cu/C film support the function of Au in the Au/C film.

Author reply: We thank the Reviewer for their continued attention to this point. As detailed in our response to the first comment, we have undertaken a major revision to focus the manuscript exclusively on the Cu/C system. Consequently, all experimental data and simulation results previously pertaining to the Au/C film have been replaced with a complete, self-consistent dataset for the Cu/C film. Therefore, in the Revised manuscript, the proposed scientific findings and the intelligent lubrication framework are now fully and rigorously supported by the characterization and simulation evidence obtained from the Cu/C film alone. This focused approach ensures clarity and eliminates any ambiguity regarding the evidence base for our conclusions.

3. One of the key mechanisms that enables the Cu(Au)/C nanocomposite films to exhibit excellent lubricating performance is that the NPs can migrate to the surface under thermal conditions. However, Figs. 2a, b, d; Supplementary Figs. 10 and 11 are not clear enough to show this migrant trend. Supplementary Video 1 is too shaky, with something moving but not clearly visible. Additionally, those distinctive particles appear to be growing in size with increasing temperature but not moving. The author needs to clearly mark and show the migration of NPs to support their hypothesis.

Author reply: We thank the Reviewer for this careful and constructive comment, which prompted us to clarify both the evidence for nanoparticle (NP) migration and the interpretation of our TEM data. The most direct evidence of Cu NP migration is provided by the cross-sectional TEM image of the wear track in Fig. 1a of the original manuscript. This specimen was prepared by focused-ion-beam (FIB) sectioning after the friction test, allowing direct observation of the through-thickness microstructure in the worn region (Fig. R1-1a). In this geometry, Cu NPs are clearly seen to accumulate toward the friction interface, revealing their migration pathways and directions under friction-induced thermal conditions. In the Revised manuscript, we have explicitly marked these migration paths in Fig. 1a and expanded the corresponding discussion in the main text to make this evidence unambiguous.

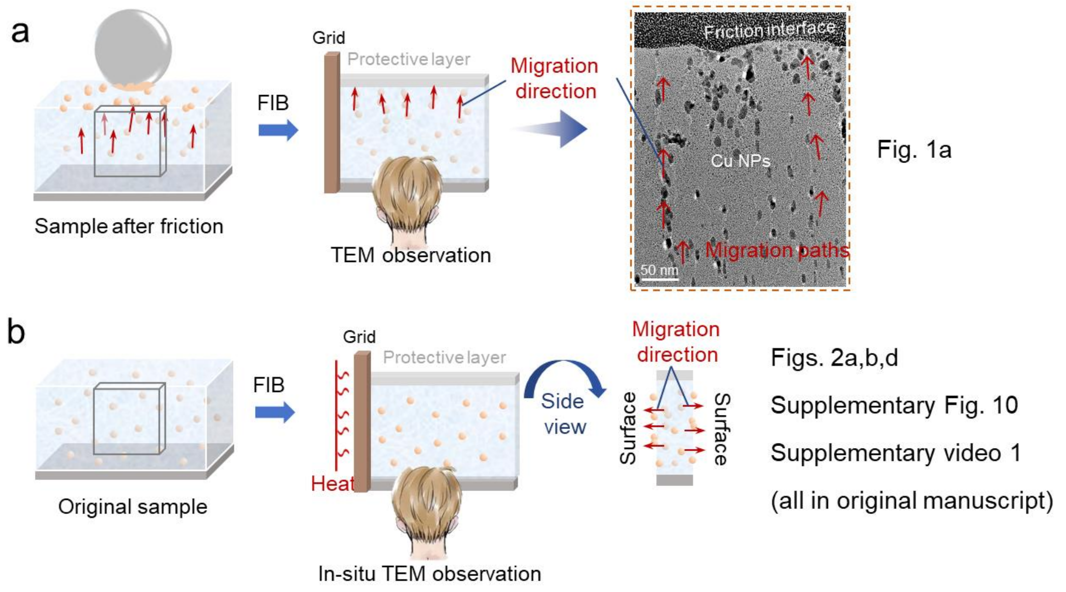

Fig. R1-1. Schematic diagram of Cu migration in a-C matrix via different TEM characterizations.

By contrast, Figs. 2a, 2b, and 2d and Supplementary Figs. 10 and 11 originate from in situ heating TEM and static HRTEM observations, which have intrinsic limitations in revealing migration trends. The in-situ TEM samples are extremely thin (<100 nm) and are observed in projection along a single viewing direction (Fig. R1-1b). Upon heating, Cu NPs preferentially migrate along the shortest available free path, which is often along the beam direction. Consequently, in the two-dimensional TEM projection this three-dimensional migration manifests primarily as an apparent increase in particle size, resulting from successive nanoparticles moving into the same projected area, rather than as clear lateral displacement. This projection effect explains why the particles appear to grow with increasing temperature in these figures, even though material transport is occurring.

Supplementary Fig. 11 shows the pristine microstructure of the Cu/C film without friction or heating and therefore does not display migration behavior. Supplementary Video 1 was indeed affected by camera instability during acquisition, which reduced the visual clarity of the migration process. To address this, Supplementary Fig. 10 presents a sequence of representative static frames extracted from the video, enabling readers to more clearly follow the structural evolution during heating. In the Revised manuscript, we have clarified these experimental constraints and explicitly discussed how sample geometry and projection effects influence the interpretation of in-situ TEM observations.

Together, the cross-sectional FIB–TEM evidence and the in-situ heating experiments provide complementary support for Cu nanoparticle migration toward the friction interface. We believe that the added annotations, revised figures, and expanded discussion now clearly substantiate this key mechanism and address the Reviewer's concern.

4. Fig. S4a exhibits period noise. What is the reason?

Author reply: Thanks for this comment, which helped us identify and address a critical experimental artifact. We conducted repeated friction tests on the Au/C film at 6 Hz (Fig. S4a) and traced the source of the periodic noise to mechanical vibration from the vacuum pump system.

To resolve this, we implemented vibration isolation measures, which successfully eliminated the periodic noise, as shown in the control data (Fig. R1-2). We note that the remaining non-periodic fluctuations in the friction coefficient at higher frequencies (6 Hz and 9 Hz) are inherent to the lubrication state of the film and not related to external vibration.

In the Revised manuscript, we have replaced the affected dataset with new friction tests performed on the Cu/C film under optimized conditions, which are free from this vibration interference. This ensures the accuracy and reliability of the tribological data presented.

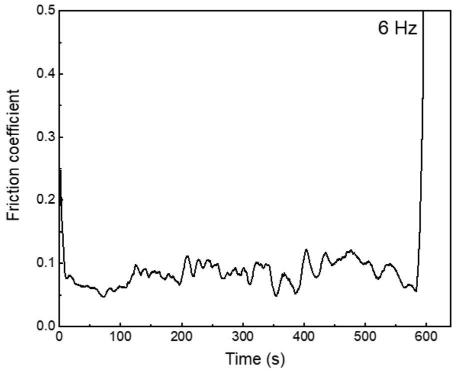

Fig. R1-2. Repetition of the friction coefficient curve of Au/C film at 6 Hz.

5. The author claims that metal migration in the film can be precisely regulated in response to frictional heat, enabling intelligent lubrication. According to Supplementary Note 1, it appears that the film can only work within a specific range of sliding speeds. For high speed, the film wears out very quickly.

The reasons for the film's failure at high speed, as well as its limitations in adjusting ability, should be discussed in the manuscript.

Author reply: We thank the Reviewer for this insightful comment and for highlighting the importance of discussing the operational limits of the self-adjusting lubrication mechanism. We agree that the intelligent lubrication behavior is effective only within a defined range of sliding conditions, and this point has now been explicitly clarified and discussed in the Revised manuscript.

Our experiments show that, in reciprocating friction tests, both the Cu/C and Au/C films operate stably only within a certain range of reciprocating frequencies (or sliding speeds) and fail rapidly at higher frequencies. As shown in Fig. R1-3, the Cu/C film fails at 3 Hz, whereas the Au/C film fails at 6 Hz, indicating a broader adaptability window for the Au/C system. This behavior can be understood in terms of a dynamic balance between the metal migration rate and the consumption rate at the friction interface, which underpins the intelligent lubrication state.

On the one hand, increasing reciprocating frequency corresponds to higher sliding speed and greater frictional heat generation, which accelerates metal nanoparticle migration and facilitates rapid establishment of a low-friction interface. This is reflected in both systems by a systematic reduction in running-in time with increasing frequency. **On the other hand**, excessively high reciprocating frequencies dramatically increase the rate of material consumption per unit time, preventing the lubrication interface from being stably maintained and requiring continuous replenishment that exceeds the system's capacity. This imbalance ultimately leads to lubrication failure.

Electrical resistance measurements provide direct evidence for this mechanism. At frequencies below 3 Hz for the Cu/C film and below 6 Hz for the Au/C film, the electrical resistance exhibits intermittent fluctuations, indicating that metal migration reaches a dynamic equilibrium and undergoes periodic self-adjustment. In contrast, at 3 Hz for the Cu/C film and 6 Hz for the Au/C film, the electrical resistance remains persistently high, signifying continuous, non-equilibrated metal migration and rapid depletion, which culminates in failure. These observations demonstrate that the self-adjusting

capability of the films is governed by the combined effects of sliding speed and frequency, which respectively control metal migration and interfacial consumption.

The superior adaptability of the Au/C film relative to the Cu/C film is attributed to both intrinsic and compositional factors. Au has a slightly lower melting point (1064 °C vs 1083 °C for Cu) and a substantially higher concentration in the film (15.82 at.% for Au compared with 2.75 at.% for Cu, from XPS), enabling more efficient migration and replenishment during friction. This is further supported by the consistently shorter running-in times observed for the Au/C film under comparable conditions. Based on these insights, the Revised manuscript discusses how improving metal migration and replenishment capacity represents a viable route to extend the operational window of intelligent lubrication systems. This can be achieved by strategies such as optimizing metal content or incorporating metallic components with lower melting points.

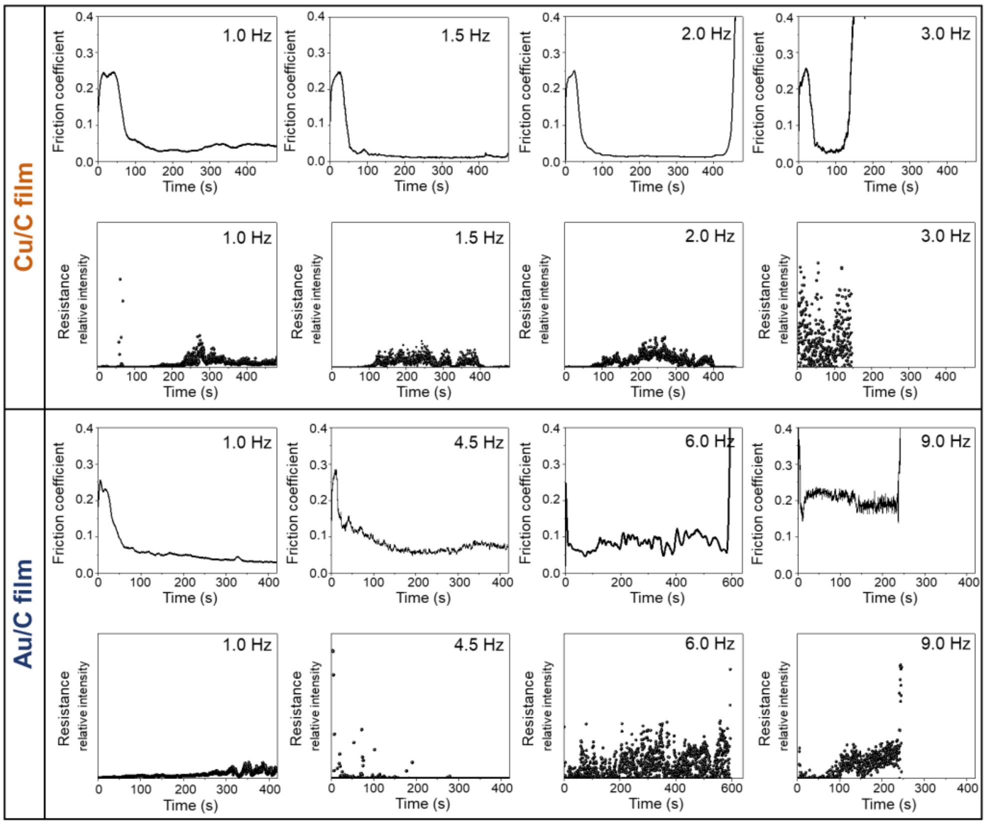

Fig. R1-3. Friction coefficient curves and corresponding electrical resistance of the Cu/C film and Au/C film at different reciprocating frequencies.

6. The author claims that the Cu/C film shows robust lubrication with an ultra-long wear life, 147 times and 38 times longer than that of hydrogen-free C films and hydrogen-containing C films, respectively.

The experimental details and results of hydrogen-free C films and hydrogen-containing C films should be provided, particularly the wear rate.

Author reply: We thank the Reviewer for this helpful comment and apologize for the insufficient description of the experimental details and comparative results for the hydrogen-free C and hydrogen-containing C films in the original manuscript. In response, we have substantially revised the manuscript to improve clarity, transparency, and quantitative comparability.

Specifically, in Fig. 4b and the Experimental Methods section, we have now added complete testing parameters, including the vacuum level, counterpart ball material, contact stress, motion mode, and sliding speed. We have also expanded the Experimental Methods section to clearly describe the preparation procedures for both the hydrogen-free C films and the hydrogen-containing C films, ensuring that the three film systems are directly comparable.

In the Results section, we now explicitly report the wear life (sliding distance to failure) and, where measurable, the wear rates of all three films during the stable friction stage. The hydrogen-free C film fails extremely rapidly (within <6 m of sliding), which prevents reliable determination of a steady-state wear rate. For this reason, the Revised manuscript focuses on a direct comparison of wear life among the three films, as presented in Fig. 4b, while reporting wear rates only where meaningful and experimentally resolvable. We believe these additions fully address the Reviewer's concern and substantiate the claim of the ultra-long wear life of the Cu/C film.

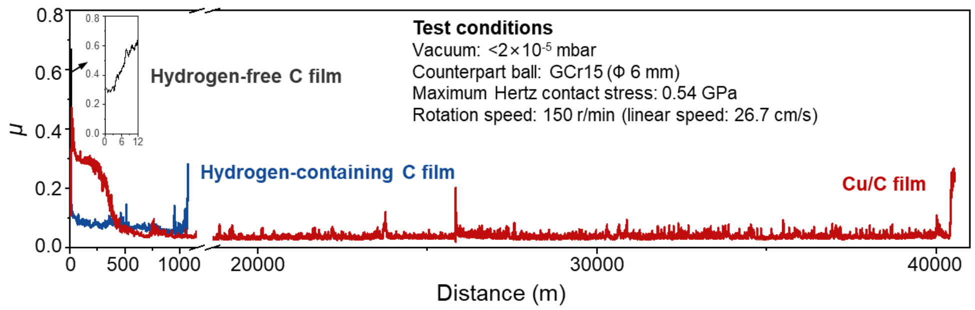

Fig. 4b. Wear life of the hydrogen-free C film, hydrogen-containing C film and Cu/C film.

Reviewer: #2 (Remarks to the Author)

The authors discovered a novel phenomenon of metal migration and catalysis to achieve intelligent lubrication in Cu or Au doped amorphous carbon-based films, significantly extending their lifespan in vacuum. Furthermore, the dynamic metal migration and catalytic mechanisms were revealed through in situ HRTEM observations in combined with simulation calculations. This study experimentally and visually demonstrates the bioinspired intelligent lubrication behavior of the Cu(Au)/C films, which is of great interest and offers a new avenue to design intelligent materials. Some points require the authors' attention before publication.

Response: We sincerely thank the Reviewer for their positive assessment and valuable feedback on our work. We are encouraged that they find the phenomenon novel and the approach of interest. Their constructive comments have been instrumental in strengthening the manuscript. We have carefully considered each point raised and have made thorough revisions to address them. We hope our detailed responses and the Revised manuscript now meet the expected standards. Thank you once again for the time and insightful guidance provided throughout the review process.

1. This work claimed that the anti-wear life of amorphous carbon films in vacuum environments was extended through intelligent lubrication of Cu(Au)/C films. How does this performance as compared with other similar materials at present?

Author reply: Thanks for raising this point of comparison. The premature failure of carbon-based lubricants in vacuum remains a well-known challenge. As summarized in Fig. R2-1, our Cu/C film achieves a wear life of >40 km in vacuum, which substantially exceeds the performance of current state-of-the-art carbon-based material systems documented in the literature. This demonstrates a meaningful advance in addressing this long-standing limitation.

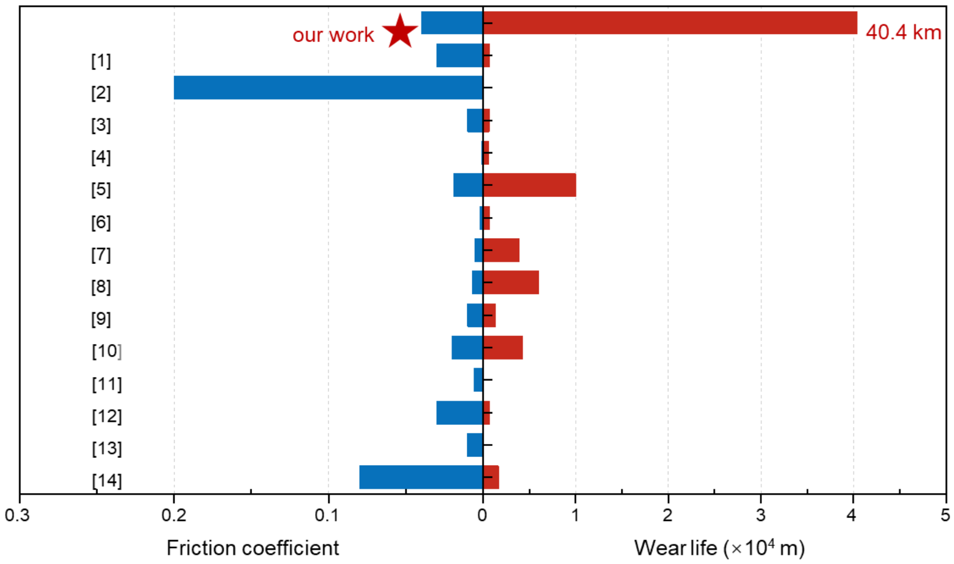

[1]. MoS_2/a-C, J. Phys. D: Appl. Phys., 2013, 46: 425301.
[2]. a-C, Tribol. Int., 2024, 193: 109345.
[3]. a-C:S:F, Carbon, 2016, 96: 411-420.
[4]. (Si, Ag)/a-C:H, Adv. Mater., 2012, 24(34): 4614-4617.
[5]. MoS_2/a-C:H multilayer, Surf. Coat. Tech., 2013, 236: 438-443.
[6]. MoS_2/a-C:H, Tribol. Lett., 2013, 52: 371-380.
[7]. Highly H-DLC, Surf. Coat. Tech.,1999, 120±121: 612-617.
[8]. Highly H-DLC, Tribol. Int., 2007, 40(7): 1186-1194.
[9]. FL-C:H, Vacuum, 2017, 143: 36-39.
[10] Textured a-C:H, J. Phys. D: Appl. Phys, 2014, 47: 235301.
[11]. a-C:H, Friction, 2022,10(12):1967-1984.
[12]. RGO/a-C:H, Nanomaterials, 2019, 9(12):1733.
[13]. F/DLC, Surf. Interface Anal., 2020, 52: 339-347.
[14]. Graphene/DLC, Diam. Relat. Mater., 2022, 125, 108998.

Fig. R2-1. Comparison of the tribological properties of our film with reported typical carbon-based materials.

2. The authors found that the melting temperature of Cu nanoparticles varies with size by in situ observations, which maybe a good method for detecting the melting temperature of nanomaterials with different sizes. It is suggested to supplement the corresponding discussion in the manuscript.

Author reply: We appreciate the Reviewer's insightful suggestion and agree that our in-situ technique has significant potential for probing the melting points of size-specific nanoparticles. We have added corresponding discussion to the Revised manuscript to address this point.

3. The migration of Cu within the amorphous carbon matrices affects the film microstructure. How about the variations in mechanical properties of the Cu doped amorphous carbon films with the above-mentioned migration?

Author reply: Thanks for this comment. Compared with the original Cu/C film， the mechanical properties of the Cu/C film remain unchanged despite the migration and precipitation of Cu nanoparticles. As shown in Fig. R2-2, after annealing at 300 °C in vacuum for 3 h, a large number of Cu nanoparticles have precipitated onto the surface of the Cu/C film. Neither the hardness nor the

elastic modulus shows deterioration compared to the original film, which indicates that the Cu/C film maintains its good mechanical properties following the aforementioned migration process.

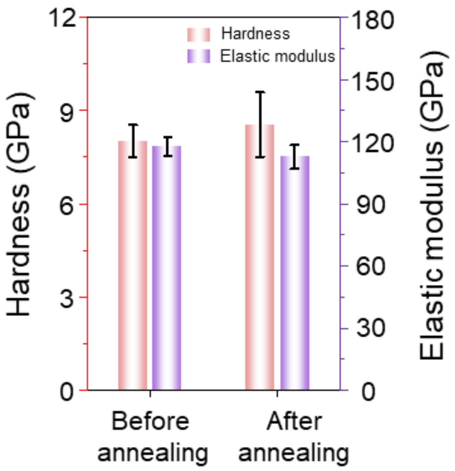

Fig. R2-2. Hardness and elastic modulus of the Cu/C film before and after annealing at 300 °C in vacuum for 3 h.

4. The authors considered that thermal effect induces the migration of doped metals. In fact, forces can also cause lattice distortion and atomic motion. Explain whether the effect of force is alone considered or not?

Author reply: Thanks for the Reviewer's thoughtful question regarding the influence of force alone in inducing metal migration. Indeed, friction inevitably generates both heat and force simultaneously. To investigate the effect of force alone, a static pressure test (a maximum Hertz contact stress of 1.34 GPa; Fig. R2-3) was conducted in vacuum for 3 h without sliding/frictional heating. The results indicate that force alone is insufficient to induce migration of Cu nanoparticles towards the surface. As depicted in Fig. R2-3, EDS analysis of the contact area reveals no notable aggregation of Cu (Fig. R2-3c and 2-3d). XPS of Cu and C (Fig. R2-3e and 2-3f) also indicts that their content and chemical bonding state are unchanged before and after the static pressure test. Therefore, the above results demonstrate that the force (stress) alone is insufficient to induce the migration of the Cu nanoparticles within the Cu/C film.

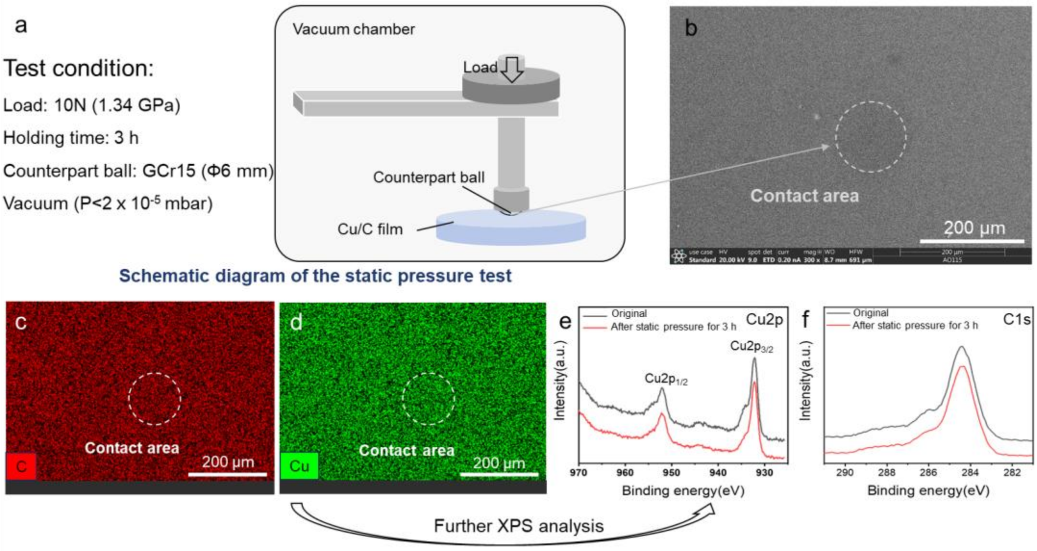

Fig. R2-3. Static pressure test. (a) Schematic diagram and test conditions of the static pressure test. (b) FESEM image of the Cu/C film surface. (c and d) Element distribution mappings of the Cu and the C element, respectively. (e) XPS of Cu 2p before and after the static pressure test. (f) XPS of C1s before and after static pressure test.

5. The authors qualitatively analyzed the changes in bonding states and interfacial electronic properties through COHP and work function calculations. If possible, adding density of states (DOS) comparisons before and after Cu incorporation would further substantiate the electronic structure evolution of carbon.

Author reply: We thank the Reviewer's constructive suggestion, which gives us an opportunity for better explanation. Following this recommendation, we have added a comparison of the DOS before and after Cu incorporation in Revised supplementary information, as shown below. The DOS of pristine carbon exhibits a pronounced peak near the Fermi level (E_f), whereas this peak is significantly suppressed after Cu incorporation, accompanied by a redistribution of electronic states over a wider energy range. These changes indicate that Cu–C interactions modify the local electronic structure of carbon and reduce its localized electronic states near the Fermi level. The added DOS analysis provides complementary evidence to the COHP and work-function calculations, further substantiating the electronic structure evolution induced by Cu incorporation. Corresponding discussions have been incorporated into both the Revised manuscript and the Supplementary Information.

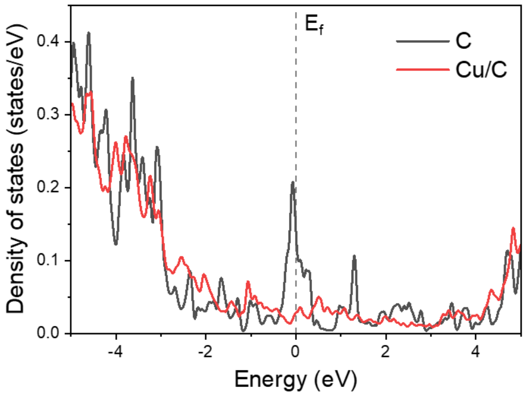

Supplementary Fig. 15. Density of states of carbon before and after Cu incorporation.

6. Some film parameters, such as thickness and mechanical properties, are important for their applications. What about the thickness, hardness, and adhesion strength of the films used in this study?

Author reply: Thank you for raising this issue. As you pointed out, the thickness and mechanical properties of films are important for their application. In this study, the thickness of the Cu/C film is 2.76 μm. The hardness of the Cu/C film is 8.01 GPa. For adhesion strength, the scratch test shows that only minor traces appear on the Cu/C film at a load of 44.28 N, which corresponds to the film-substrate adhesion, as illustrated in Fig. R2-4.

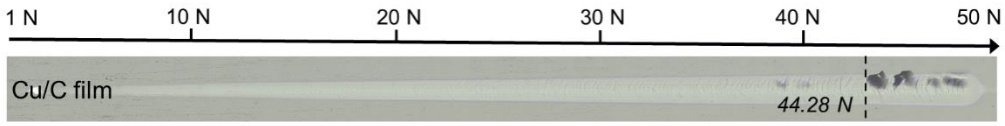

Fig. R2-4. Scratch morphology of the Cu/C film.

7. The elemental composition of the steel substrate should be provided.

Author reply: Thanks for the Reviewer's kind reminder. The steel substrates used in this work include 304 stainless and M2 high-speed two types of steel substrate. The elements of the 304 stainless steel (grade:06Cr19Ni10) mainly include Cr, Ni, Mn, Si, C, P, and S, while those of the M2 high speed steel (grade:W6Mo5Cr4V2) mainly include W, Mo, Cr, V, C, Si and Mn. To clearly show the primary elemental composition of the steel substrates in our work, we have added their grade to the Methods section in the Revised manuscript.

8. The authors constructed various configurations, including bulk-mixed and interfacial models, to capture the key states of Cu–C interactions and developed a NEP potential to describe the corresponding interatomic forces. How reliable is the developed NEP potential?

Author reply: We thank the Reviewer raising this important point. To better demonstrate the reliability of the developed NEP model, we have added additional benchmarking comparisons between NEP and first-principles calculations and experimental data, as shown in Fig. R2-5.

Specifically, the equation of state (EOS) for bulk Cu, diamond, and amorphous carbon shows only minor deviations between NEP and DFT across a broad range of volumes, indicating that the NEP model faithfully captures the energetic response to volumetric deformation. Furthermore, the phonon dispersions of bulk Cu and diamond demonstrate close agreement between NEP (dashed blue) and DFT (solid red) along the Γ–M–K–Γ high-symmetry path, confirming that the local curvature of the potential energy surface is accurately reproduced. In addition, the radial distribution function obtained from NEP-based molecular dynamics simulations is in good agreement with experimental data, both in terms of peak positions and their relative intensities.

In summary, these benchmarks demonstrate that the developed NEP potential provides a reliable and physically consistent description of Cu–C interatomic interactions within the scope of this study.

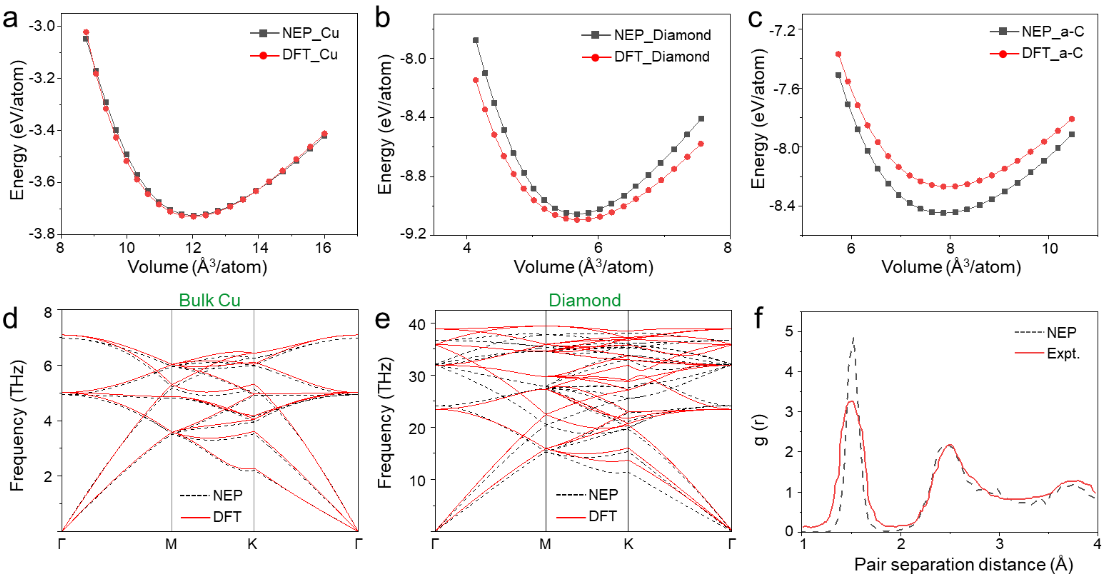

Fig. R2-5. Comparison between the NEP model and reference data. (a-c) Equation of state of bulk Cu (a), diamond (b), and a-C (c) from NEP and DFT. (d-e) Phonon dispersions of bulk Cu (d) and diamond (e) along Γ–M–K–Γ from NEP (dashed blue) and DFT (solid red). (f) Radial distribution function of a-C from NEP-based MD and experiment data extracted from wang *et al*. (*Physical Review B* **111**, 094205 (2025)).

9. Some details need to be verified, for example

a) It seems that the images in Fig. 3 are out of order.

Author reply: Thanks for pointing this issue. We have corrected the order of images in Fig. 3 in the Revised manuscript.

b) Check "Cu(" and "C(" in S2. If it is a special symbol, please provide an annotation and explanation.

Author reply: We appreciate the Reviewer for raising this question. "Cu(" and "C(" in S2 are mistake rather than special symbols. We have corrected and revised the formula in the Revised manuscript.

$$\rho_{\mathrm{diff}}(x,y,z) = \rho_{\mathrm{tot}}(x,y,z) - \rho_{\mathrm{Cu}}(x,y,z) - \rho_{\mathrm{C}}(x,y,z) \quad \text{(S2)}$$

where $\rho_{\mathrm{tot}}(x,y,z)$, $\rho_{\mathrm{Cu}}(x,y,z)$, $\rho_{\mathrm{C}}(x,y,z)$ are the charge density of Cu–C, Cu and C.

c) Ensure consistent capitalization formatting for titles in the references.

Author reply: We thank the Reviewer's careful checks. We have corrected the capitalization formatting for titles in the Revised manuscript.

d) It is mentioned "The depth of wear tracks was measured via the three-dimensional white light interferometry (UP-Lambda, Rtec-Instruments, America) and further calculated the wear rates". Grammatical revision is needed for this sentence.

Author reply: Thanks for this comment. We have made corresponding corrections in the Revised manuscript, listed as follow:

"The depths of wear tracks were measured via the three-dimensional white light interferometry (UP-Lambda, Rtec-Instruments, America) and the wear rates were further calculated from these data."

Reviewer: #3 (Remarks to the Author)

This paper proposes and validates a class of biomimetic intelligent self-regulating lubricating nanocomposite films. By harnessing heat generated within friction pairs to activate phase transitions, self-migration, and catalytic behavior in metallic nanoparticles, it establishes a closed-loop self-regulating lubrication system. Comprehensive in-situ characterization and multi-scale simulations provide robust theoretical and experimental evidence. However, several questions are still required to further clarify.

Response: We sincerely thank the Reviewer for her/his careful reading and constructive suggestions, which have significantly improved the presentation and rigor of our work. We have carefully considered all points raised and have undertaken thorough revisions accordingly. These include supplementing important new experimental data (such as quantitative analysis and TEM characterizations) and simulations, as well as refining relevant statements throughout the manuscript. We believe our detailed responses and the Revised manuscript now provide the necessary clarification and hope these changes meet with the Reviewer's approval.

1. Regarding the stability of metal supply during prolonged cycling, whilst the authors demonstrate the sustainability of the metal reservoir, the paper lacks quantitative metrics (total metal content, consumption rate, etc.). Quantitative analysis should be supplemented.

Author reply: We appreciate the Reviewer's insightful suggestion. To quantitatively understand the sustainability of the metal reservoir during prolonged cycling, we characterized the Cu content and wear volume (wear track depth) of the Cu/C film at different friction stages, and calculated the Cu consumption rate. Quantitative analysis data and discussion have been supplemented in the Revised manuscript and the Supplementary information, which is helpful for gaining deeper insights into the underlying mechanism and improving the quality of the paper. The specific revisions are as follows:

The wear track depth of the Cu/C film and Cu consumption at different friction stages (after the running-in stage, after about 5000 m, and after over 10000 m) are shown in Supplementary Fig. 18. During the running-in stage (corresponding to a sliding distance of ~480.8 m), the Cu/C film suffers

severe wear, with a considerable Cu consumption of about 2.46 at.%. The Cu consumption rate reaches 5.12×10^{-3} at.%/m, and the wear track depth is 166.7 nm. Subsequently, the friction system stabilizes, with the Cu content at around 22.95 at.% and the corresponding Cu consumption at 3.26 at.%, while the wear track depth further increases by only 49.2 nm after 5000 m of friction. When the sliding distance exceeds 10000 m, the Cu content decreases by a mere additional 0.41 at.%, reflecting a low Cu consumption rate of 3.77×10^{-4} at.%/m, while the wear track depth further increases by a further 64.8 nm. In summary, severe wear predominantly occurs during the running-in stage, whereas the wear decreases significantly in the stable friction stage. The Cu consumption in the Cu/C film remains very low over prolonged sliding, demonstrating that the Cu/C film can exhibit long-term low-friction performance.

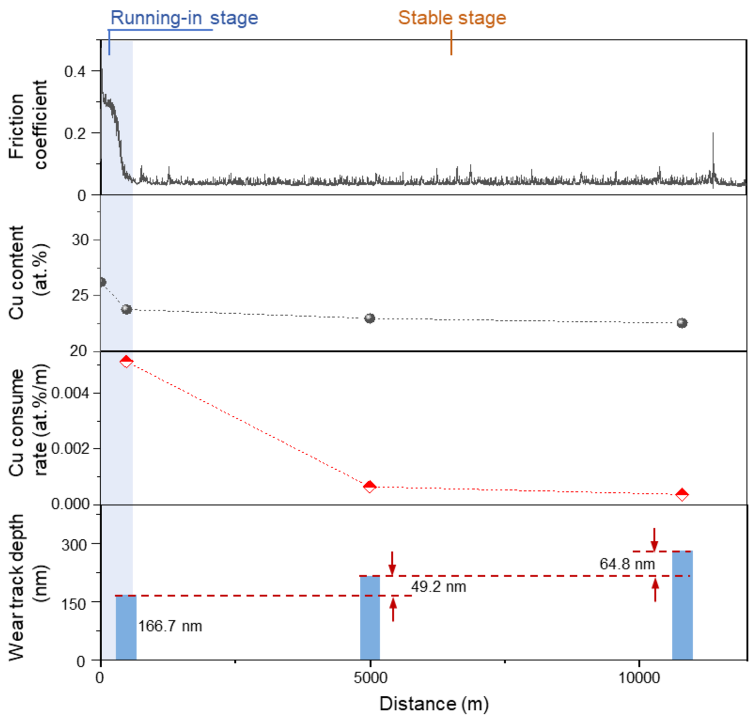

Supplementary Fig. 18. Friction coefficient curve, Cu content, Cu consume rate, and wear track depth of the Cu/C film at different friction stages.

These quantitative results provide valuable insights into understanding the mechanism. To be specific, during the initial running-in stage, metal migration is relatively slow, and an effective lubricating interface has not yet fully formed. The process is dominated by mechanical wear, leading to significant wear and requiring substantial metal migration until a stable lubricating interface is established. Thereafter, the lubrication state at the friction interface gradually stabilizes, reaching a

dynamic equilibrium between metal replenishment and consumption at the frictional interface. Even if the lubrication state occasionally deteriorates, only a small amount of Cu metal needs to be supplied to the frictional interface to maintain lubrication state.

This mechanism is also supported by other experimental results. For example, Fig. R3-1 shows that the running-in stage shortens with increasing sliding speed (frequency) due to the enhanced driving force for migration. Additionally, Fig. 1b indicates that when a new counterpart ball is used, the running-in stage becomes very short, because after the first round of friction process, migration pathways has been formed, and Cu can migrate to frictional interface rapidly.

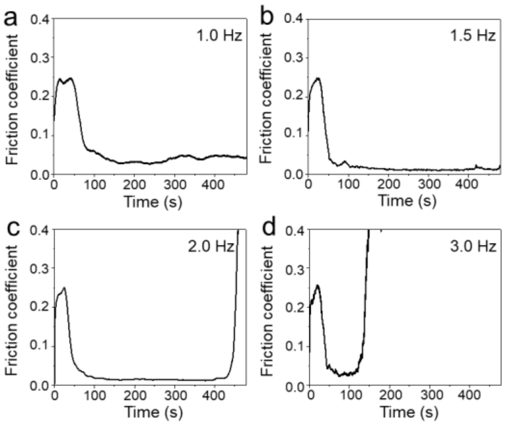

Fig. R3-1. Friction coefficient curves at different reciprocating frequencies (a) 1.0 Hz, (b) 1.5 Hz, (c) 2.0 Hz, (d) 3.0 Hz.

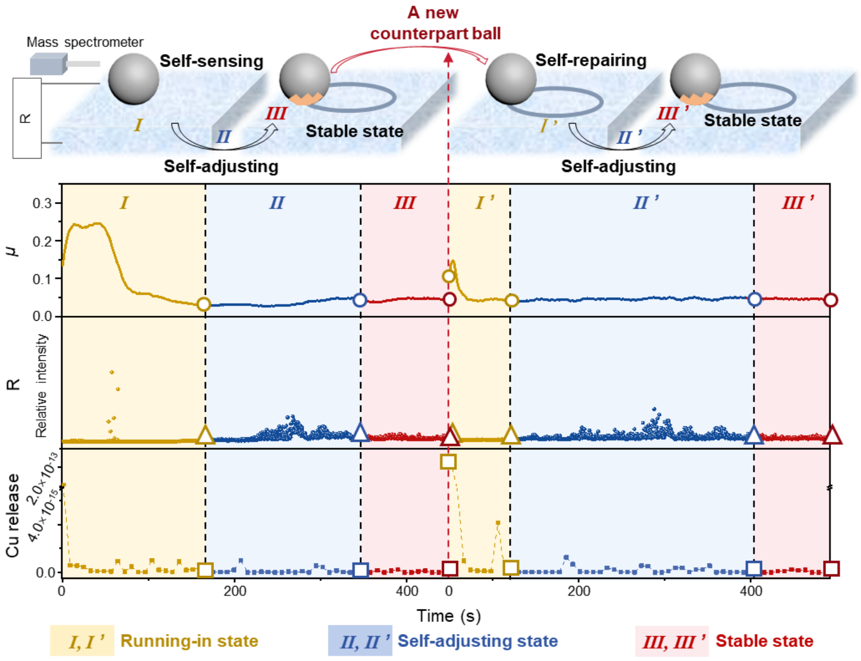

Fig. 1b. Real-time monitoring results of μ, electrical resistance (R) and Cu release during two consecutive friction processes of the Cu/C film.

2. The catalytic formation of ordered carbon structures is substantiated solely by local TEM images, lacking statistical data to confirm its prevalence.

Author reply: Thanks for this comment. Following the Reviewer's helpful recommendation, we have supplemented high-resolution TEM (HRTEM) images of samples during in-situ heating and at the friction interface in the Supplementary information (Supplementary Figs. 12 and 20). These statistical data (Fig. R3-2) demonstrate the prevalence of the catalytic formation of ordered carbon structures.

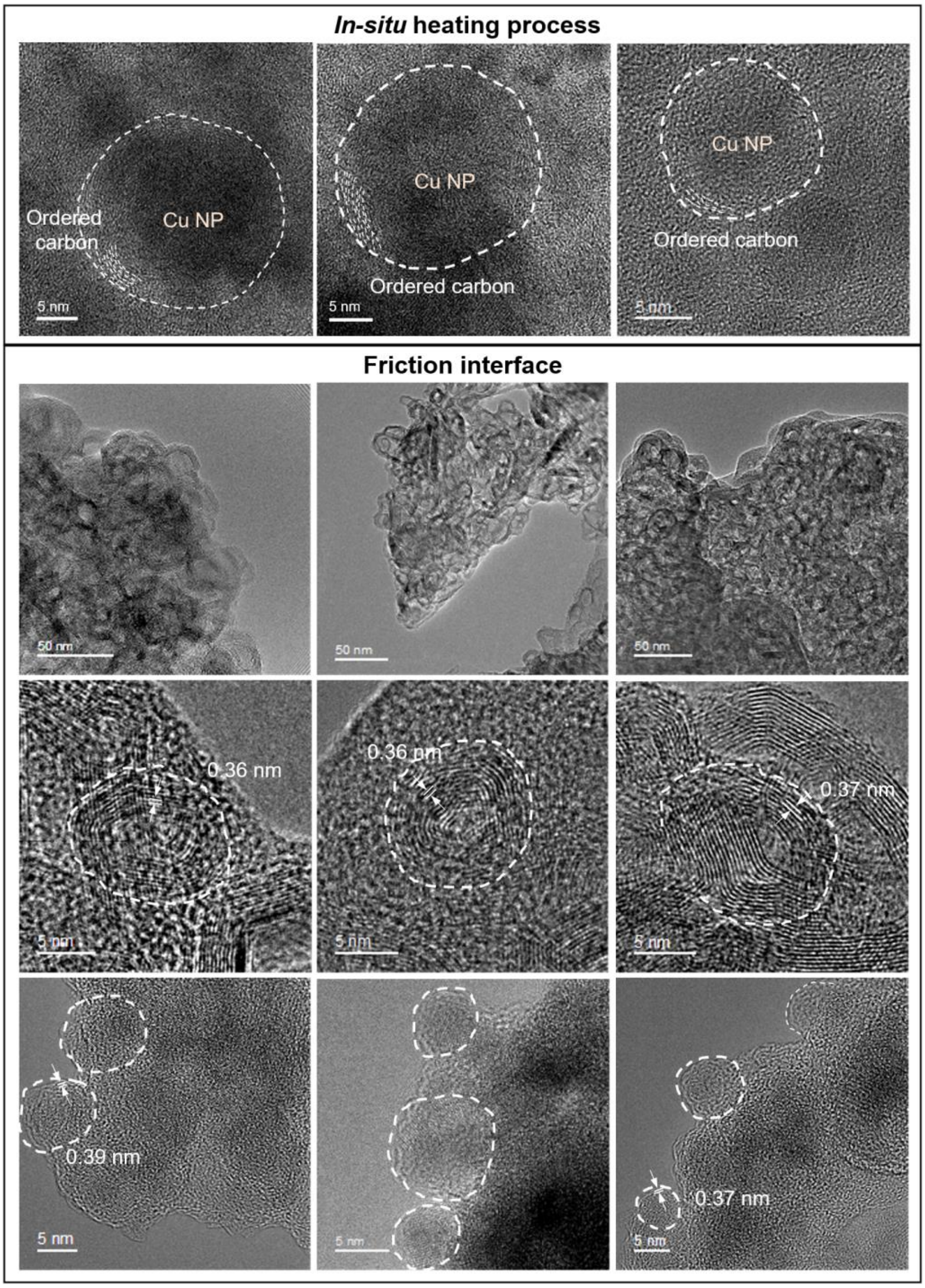

Fig. R3-2. HRTEM images of ordered carbon structures during in-situ heating process and at the friction interface.

3. The rationale for selecting nonporous structures in the model warrants further discussion. While MD simulations employed a single conical pore configuration, actual thin-film pore distributions exhibit greater complexity.

Author reply: We are greatly appreciative for this insightful comment. We agree that pore morphologies in real thin films are more complex than an idealized geometry. Our initial selection of a conical pore model was guided by experimental observations, as cross-sectional TEM images (e.g., Fig. R3-3) show that many nanoscale pores in these films exhibit a funnel-like morphology. This makes the cone a reasonable first-order approximation to capture the dominant confinement and directional driving effects. However, we fully acknowledge that pore shape complexity may influence detailed migration behavior.

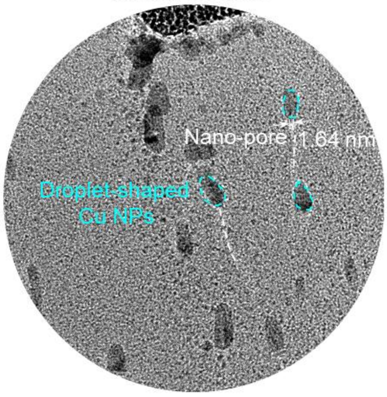

Fig. R3-3. Cross-sectional HRTEM image of the wear track of the Cu/C film at stable friction stage.

To address this point, we extended our model beyond an ideal cone by introducing random surface roughness to construct a quasi-conical pore geometry, which more closely resembles experimentally observed nanopores. As shown in Supplementary Fig. 10a, the optimized pore includes stochastic roughness superimposed on the ideal conical wall, leading to a more realistic pore profile. We then performed additional MD simulations using this quasi-conical pore model. Importantly, despite the increased geometric complexity, we consistently observed the migration of the Cu nanoparticle (NP) toward the pore direction, as demonstrated by the time evolution of particle displacement in Supplementary Fig. 10b. This result indicates that the key migration behavior reported in the manuscript is robust with respect to pore-shape variations and is not an artifact of assuming a perfectly

smooth conical pore. Moreover, we have modified the relevant description, Supplementary Fig. 10, and Fig. 2e in revised version.

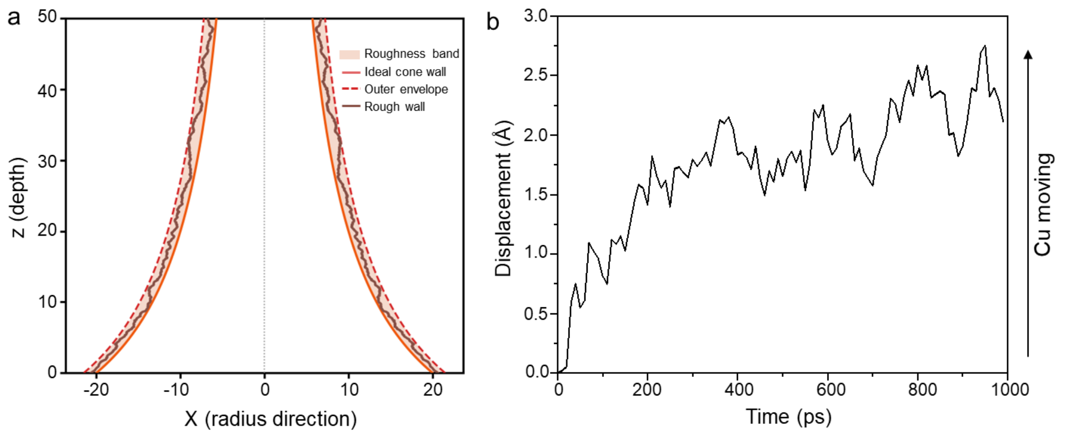

Supplementary Fig. 10. (a) Schematic comparison between an ideal conical pore and a quasi-conical pore with randomly introduced surface roughness. (b) Time evolution of the displacement of a Cu NP in the quasi-conical pore obtained from MD simulations.

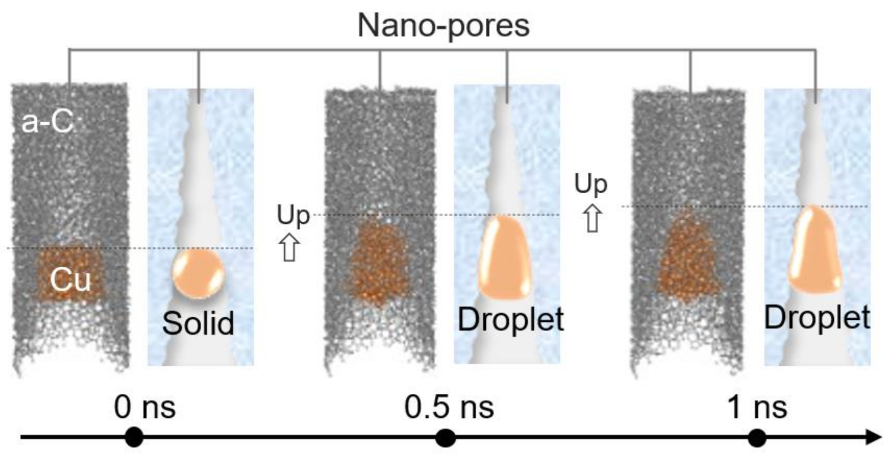

Fig. 2e. MD simulations and schematic diagram of a Cu NP migrating along the nanopores.

4. The role of Au is not sufficiently elaborated. Although the paper refers to Cu (Au)/C, the results predominantly feature Cu/C, with Au only marginally addressed in transfer film observations.

Author reply: Thank you very much for raising this question which we acknowledge was not sufficiently elaborated in the original manuscript. We have revised our approach to ensure clarity and focus. Our initial studies did investigate both Cu- and Au-doped carbon films, as both systems exhibited qualitatively similar metal migration and tribological behavior. We recognize, however, that presenting their results interchangeably lacked the rigor required to draw definitive material-specific conclusions.

To resolve this, we have undertaken a substantial revision to establish a clear and robust narrative. The revised manuscript is now focused exclusively on the Cu/C film, supported by a complete, self-consistent dataset of in-situ TEM, tribological tests, and theoretical simulations. This consolidated evidence provides a strong foundation for the proposed intelligent lubrication framework. Consequently, all data from the Au/C system have been replaced with corresponding results from the Cu/C film.

Regarding the Au/C film specifically, we agree that a deeper mechanistic study is of great interest. While we have made attempts to pursue additional characterization, practical constraints such as limited access to specialized in-situ TEM facilities have delayed a comprehensive investigation. Following the reviewer's insightful guidance, we plan to systematically investigate a series of soft metals (including Au and Ag) in future work, explicitly exploring how variations in properties like melting point and catalytic activity influence the intelligent lubrication mechanism. This will form the basis of a subsequent, comparative study.

5. The process annotations in Figure 1b (states I–VI) require enhanced explanation, clearly elucidating the physical significance of each stage.

Author reply: We appreciate this suggestion. To clarify the intelligent lubrication process of the Cu/C films, we have revised the annotations in Fig. 1b and supplemented descriptions of the physical significance of each stage in the Revised manuscript as follows.

The process unfolds in three distinct stages. In the initial running-in stage (*I*), before a stable lubricating interface forms, μ is high. This state stimulates substantial Cu NP migration toward the surface, reflected by sharp fluctuations in electrical resistance and continuous Cu release. Once a primary lubricating interface of ordered carbon is established, the system enters a self-adjusting stage (*II*). Here, minor increases in μ trigger slight, corrective replenishment via Cu migration, evidenced by small, correlated fluctuations in electrical resistance and metal release. Ultimately, a robust, ordered carbon interface is maintained in a stable state (*III*), where friction remains low and stable, accompanied by steady electrical resistance and a near-constant, low level of Cu release over extended

periods. Furthermore, the system demonstrates a self-repair capacity. Introducing a new counterpart ball disrupts the friction interface, causing the system to swiftly repeat the cycle from Stage *I* to *III*, thereby repairing the lubricating interface.

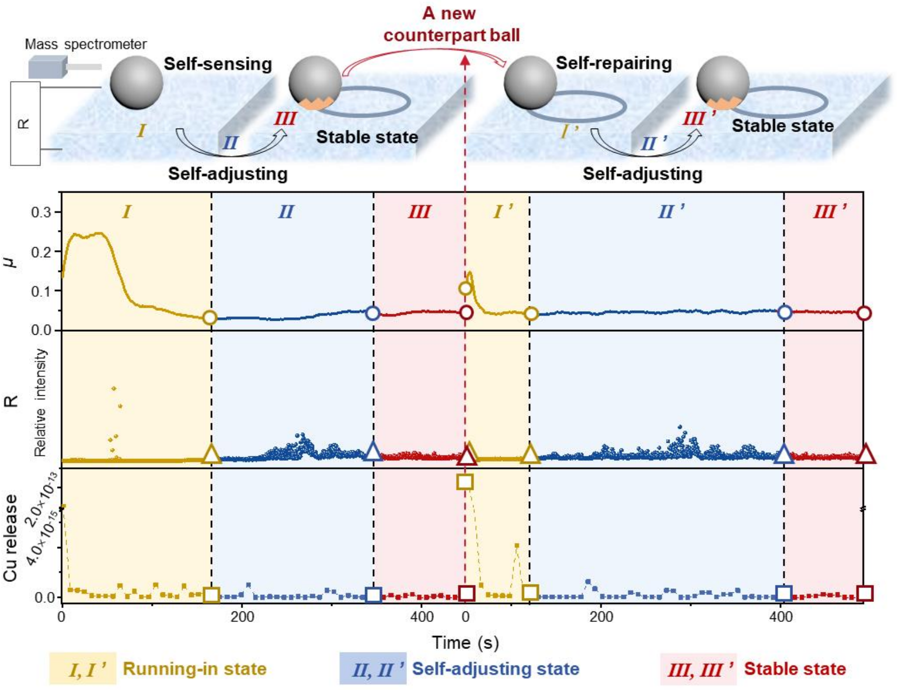

Fig. 1b. Real-time monitoring results of μ, electrical resistance (R) and Cu release during two consecutive friction processes of the Cu/C film.

6. Some TEM and IFFT images in Figure 2 are insufficiently clear. It is recommended to enhance contrast or annotate key crystal planes.

Author reply: We thank the reviewer for pointing out this issue. In response, we have improved the clarity of the TEM and IFFT images in Figure 2 by enhancing their contrast and providing clear annotations of the major crystal planes. The updated, higher-quality figures are presented below and are included in the Revised manuscript.

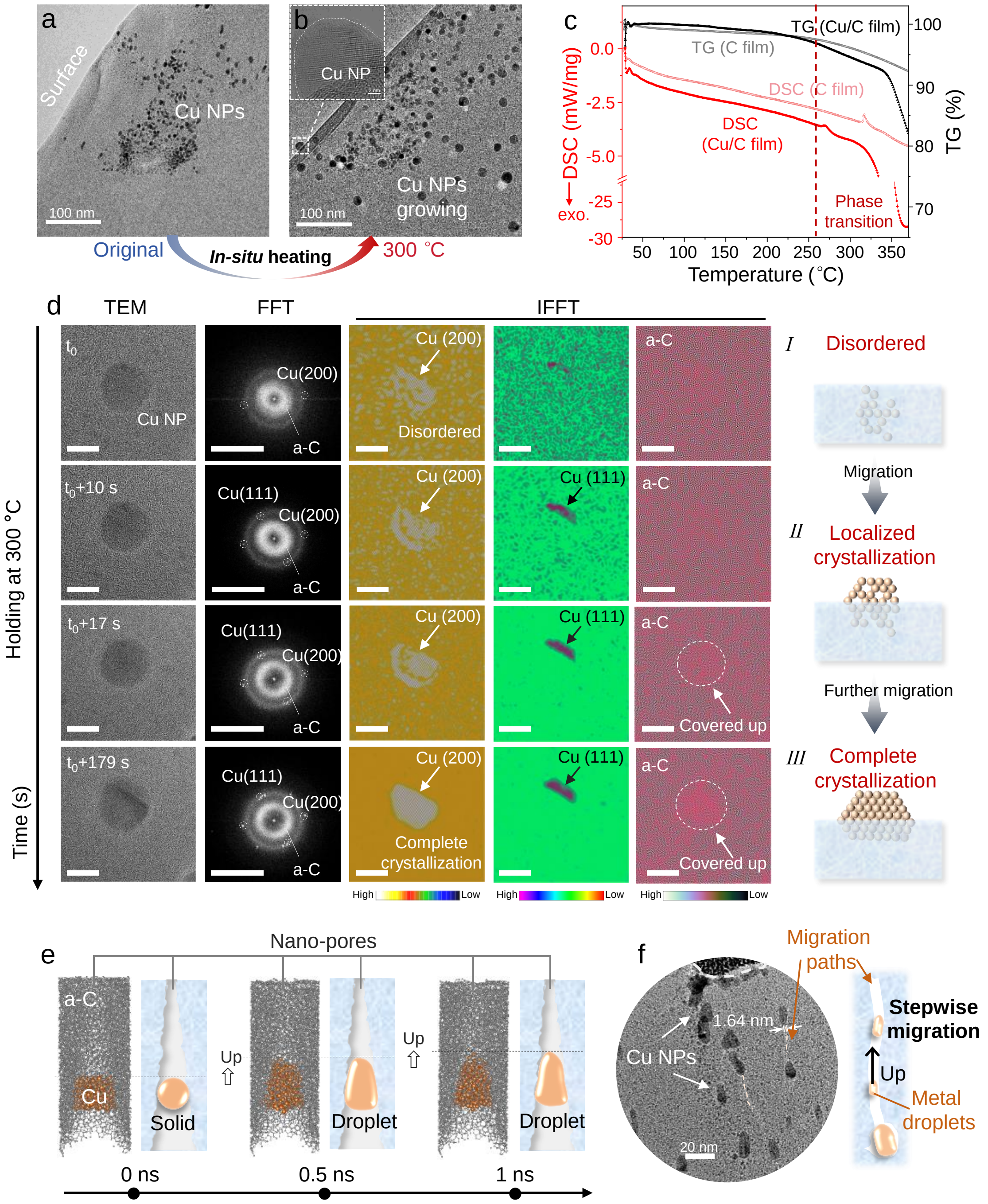

Fig. 2. Migration mechanism of heat-induced solid-liquid phase transition of Cu NPs: (a, b) In-situ HRTEM images of the Cu/C film during heating from room temperature to 300 °C, (c) DSC and TG curves of C and Cu/C films, (d) HRTEM images, fast Fourier transform (FFT) and IFFT patterns of Cu (200), Cu (100), and a-C, as well as schematic diagram of the microstructural evolution of the Cu/C film during holding at 300 °C (Scale bars in the TEM and IFFT images are 10 nm, while scale bars in FFT images is 10 1/nm), (e) MD simulations and schematic diagram of a Cu NP migrating along the nanopores, and (f) Cross-sectional TEM image of the wear track and schematic diagram of stepwise migration of Cu droplets.

7. The energy barrier diagram in Figure 3 would be more readable if molecular structures representing reaction pathways were incorporated.

Author reply: We thank the Reviewer for the helpful suggestion. Incorporating molecular configurations along the reaction pathway would indeed improve the clarity of the energy barrier

diagram. In response, we have enhanced Figure 3 by adding annotations for six key molecular structures that illustrate the primary stages of the catalytic process (now shown in Fig. 3f). To provide a complete reference, we have also included the full set of atomic configurations for the entire reaction pathway in the Supplementary information (Supplementary Fig. 13).

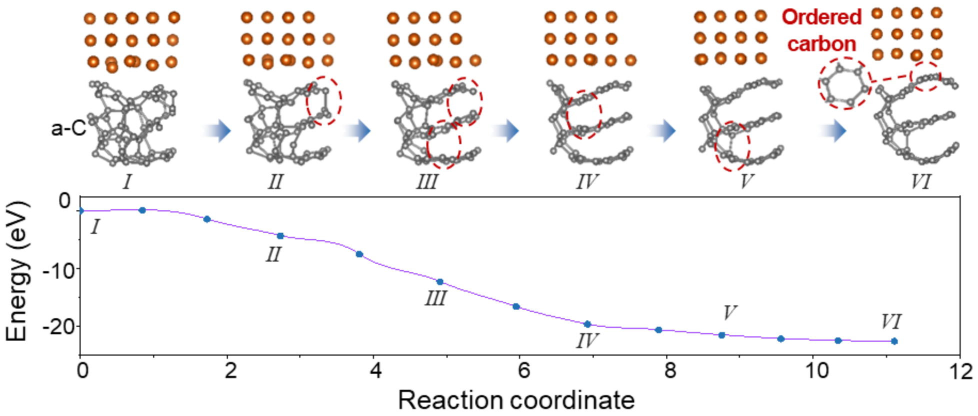

Fig. 3f. Energy changes in the Cu-catalyzed transformation from a-C to ordered carbon (*I*. a-C; *II*-*V*. sp^3C breakage and sp^2C recombination; *VI*. Ordered carbon).

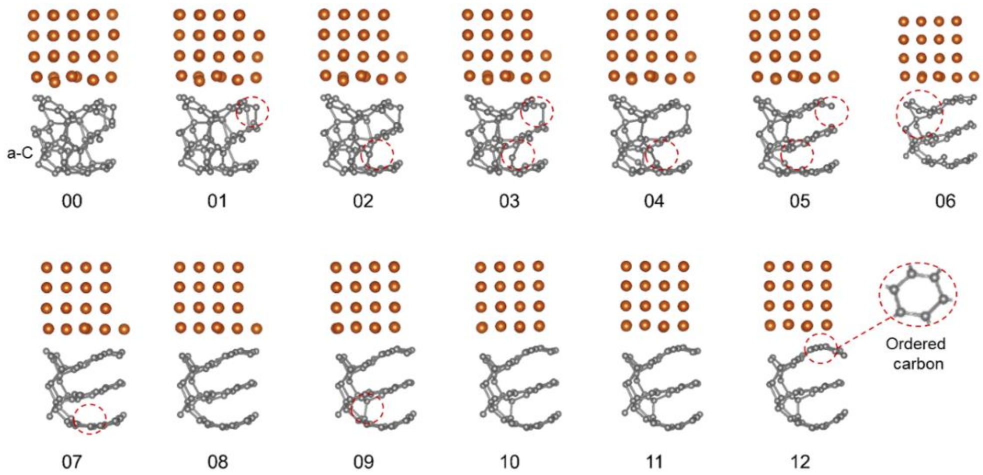

Supplementary Fig. 13. Atomic configurations along the reaction pathway obtained from the CI-NEB method. (Frame 00 corresponds to the initial state and frame 12 corresponds to the final state, with intermediate frames representing the transition pathway between them).

8. Unit formats throughout the paper need standardization.

Author reply: Thanks for this comment. We have carefully checked the entire manuscript and unified the unit format in the revised version. The key modifications are listed as follows:

a. We have unified unit collocation and typography, and ensured that all numbers are separated from units by a space (e.g., "270 °C" instead of "270°C").

b. We have unified the format of composite units with correct slashes and superscripts. (e.g., changed “eV $Å^{-1}$” to " eV/Å”).

9. The language is required to further refine its clarity and readability.

Author reply: Thank you for this comment. We have made effort to improve the language to enhance its clarity and readability, and we also invited English-proficient teachers to help revise the manuscript.

Reviewer: #4 (Remarks to the Author)

The manuscript reports Cu(Au)/C nanocomposite films designed as bioinspired lubricants with self-adjusting and self-repairing capabilities under friction. The authors integrate in-situ TEM, Raman spectroscopy, DSC-TG, electrical measurements, tribological testing, MD simulations, and DFT calculations to establish a mechanistic framework in which friction-induced heat triggers metal nanoparticle melting, migration, and catalytically driven ordering of carbon nanostructures.

Although the concept of in-situ tribofilm formation and self-lubrication has been explored previously (Nature 536, 67–71 (2016); Carbon 138, 61–68 (2018); Mater. Today Chem. 26, 101112 (2022); ACS Appl. Mater. Interfaces 15, 30070–30082 (2023)), the depth of investigation, mechanistic correlation, and tribological performance demonstrated in this work are impressive. The study is comprehensive and experimentally rigorous, offering detailed mechanistic insights that are potentially valuable to the field of tribology and related disciplines.

However, several scientific claims require solid supporting evidence and/or clearer presentation before the manuscript can be considered for publication. A major revision is required before the manuscript can be considered for potential acceptance.

Response: We sincerely appreciate the Reviewer's thorough evaluation and positive feedback on our work. We are delighted that the Reviewer's consider our study "comprehensive and experimental rigorously, offering detailed mechanistic insights that are potentially valuable to the field of tribology and related disciplines." We have carefully considered all the points regarding scientific claims that require further evidence or clearer explanation. In the Revised manuscript, we have supplemented and addressed each comments point-by-point (listed as below) by supplementing data, refining experiments, and improving textual clarity to ensure all conclusions are robustly supported. We believe these revisions have strengthened the scientific quality and persuasiveness of the work. We are grateful for the Reviewer's valuable guidance in further improving this work and hope the revised version meets with the Reviewer's approval.

1. The central concept that friction-induced solid-liquid transition of metal nanoparticles enables

autonomous replenishment and catalytic ordering of carbon is compelling and represents a distinctive mechanistic pathway for self-regulated lubrication. However, the manuscript overstates "bio-inspired intelligence," which is essentially an intelligent feedback loop driven by frictional heat rather than a physical mimicry of any biological structure. Moreover, the concepts of self-sensing and self-regulation are not unique to biomimetic systems; they are recognised across engineering and materials science without necessarily implying biological inspiration.

Author reply: We thank the Reviewer for this insightful observation. We fully agree that the self-replenishing and self-regulating lubrication behaviors revealed in this study are essentially an intelligent feedback loop driven by frictional heat rather than a physical mimicry of any biological structure. Following the reviewer's suggestion, we have removed the statements of "bio-inspired intelligence" throughout the manuscript. The main revisions are as follows:

New article title: **An intelligent feedback loop for sustaining self-lubrication and wear resistance**.

Introduction and Discussion sections: we have rewritten the description to avoid explicitly linking "self-sensing" or "self-regulation" to biomimetic systems. Instead, these concepts are now discussed within the broader context of engineering and materials science.

2. The proposed feedback cycle relies on the tight coupling among friction coefficient, resistance, metal release, and interfacial structure. While qualitative trends are shown, quantitative correlations are limited. It is recommended to provide cross-correlation analysis to confirm causality rather than coincidence.

Author reply: We thank the reviewer for raising this comment. In the Revised manuscript and the Supplementary information, we have supplemented the synchronous measurements of the friction coefficient, electrical resistance, and metal release for the Cu/C film (Fig. 1b), and the cross-correlation analysis was also performed according to the Spearman's rank correlation coefficient. For two time series x and y sampled at paired time points, Spearman's coefficient is defined as the Pearson correlation of their rank-transformed variables (*The American Journal of Psychology* **100**, 441-71 (1904)):

$$\rho_s = \frac{\sum_{i=1}^{n}\left(r(x_i) - \overline{r(x)}\right)\left(r(y_i) - \overline{r(y)}\right)}{\sqrt{\sum_{i=1}^{n}\left(r(x_i) - \overline{r(x)}\right)^2}\sqrt{\sum_{i=1}^{n}\left(r(y_i) - \overline{r(y)}\right)^2}}$$

where ρ_s is Spearman's rank correlation coefficient; n is the number of paired observations; x_i and y_i are the i-th samples of variables x and y, respectively; $r(x_i)$ and $r(y_i)$ denote the ranks of x_i and y_i, respectively. The rank operator $r(\cdot)$ assigns the rank in the presence of ties, defined as $r(x_i) = 1 + \sum_{j=1}^{n} 1(x_j < x_i) + \frac{1}{2}\sum_{j=1}^{n} 1(x_j = x_i)$, and analogously for $r(y_i)$, where $r(\cdot)$ is the indicator function. The mean ranks are $\overline{r(x)} = \frac{1}{n}\sum_{i=1}^{n} r(x_i)$ and $\overline{r(y)} = \frac{1}{n}\sum_{i=1}^{n} r(y_i)$.

The analysis shows that, while the qualitative trends among friction coefficient, electrical resistance, and metal release are consistent, their overall quantitative correlations are moderate rather than uniformly strong. This reflects the intrinsically multivariate nature of the friction process, in which no single parameter uniquely determines the system response. During the running-in stage (Stage *I*), mechanical wear dominates and the catalytic lubricating interface has not yet formed, leading to weak quantitative correlations. In the stable stage (Stage *III*), a mature lubricating interface is established and metal migration largely saturates, again resulting in weak coupling among the three signals. By contrast, in the self-adjusting stage (Stage *II*), where active metal migration and interfacial reconstruction jointly regulate the lubrication state, the correlations become pronounced, with relatively high Spearman coefficients (Supplementary Fig. 3).

We also note that temporal asynchrony further reduces apparent correlation strength. Changes in friction first trigger metal phase transitions and migration, which inherently require finite time, and the mass spectrometry detection of released metal introduces an additional delay of several seconds. These physical and instrumental lags are explicitly captured by the lagged correlation analysis.

Following the reviewer’s recommendation, the above cross-correlation methodology, results, and associated discussion have been incorporated into the Revised manuscript to more clearly substantiate the causal coupling underlying the proposed feedback cycle.

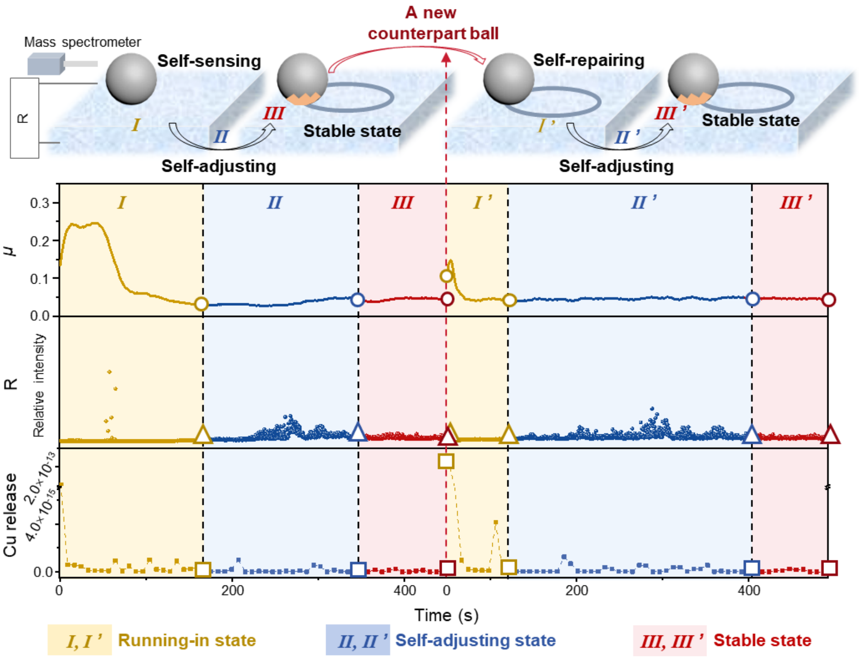

Fig. 1b. Real-time monitoring results of μ, electrical resistance (R) and Cu release during two consecutive friction processes of the Cu/C film.

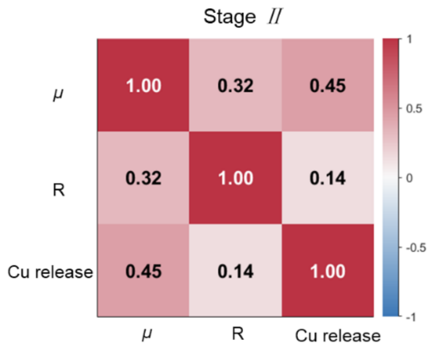

Supplementary Fig. 3. Correlation heatmap of friction coefficient (μ), electrical resistance (R) and Cu release during the self-adjusting stage (*II*).

3. The dominant results and discussion focus on the Cu/C nanocomposite, while only Fig. 1(b) and Supplementary Figs. 5 and 6 pertain to the Au/C system. A comparison between the Cu/C and Au/C systems should be provided, including their tribological performance and the corresponding mechanistic framework, to clearly distinguish the roles of Au and Cu. This is particularly important because Au exhibits a relatively low melting temperature at the nanoscale, stronger catalytic activity, and significantly higher cost, all of which influence the lubrication mechanism and practical relevance.

Author reply: We thank the Reviewer for her/his insightful and constructive suggestion. In our initial study, both Cu-doped and Au-doped carbon films exhibited qualitatively similar metal migration phenomenon (Fig. R4-1) and tribological behaviors (Fig.1b in Original and Revised manuscript). We

acknowledge, however, that presenting their results interchangeably was not methodologically sound, given the material-specific differences in properties such as melting point and catalytic activity that the reviewer rightly notes.

To establish a robust and unambiguous foundation for the proposed lubrication framework, we have undertaken a major revision. The manuscript now focuses exclusively on the Cu/C system, supported by a complete, self-consistent dataset including in-situ TEM, tribological tests, and simulations. This focused narrative allows us to present a clear mechanistic pathway without conflation.

We fully agree that a systematic, comparative study of different metal dopants (including Au and Ag) is a compelling and logical next step. Such work would directly explore how properties like melting point influence the adaptive mechanism, as the reviewer suggests. While practical constraints currently limit a full parallel investigation of Au/C, we plan to pursue this comparative analysis in future research.

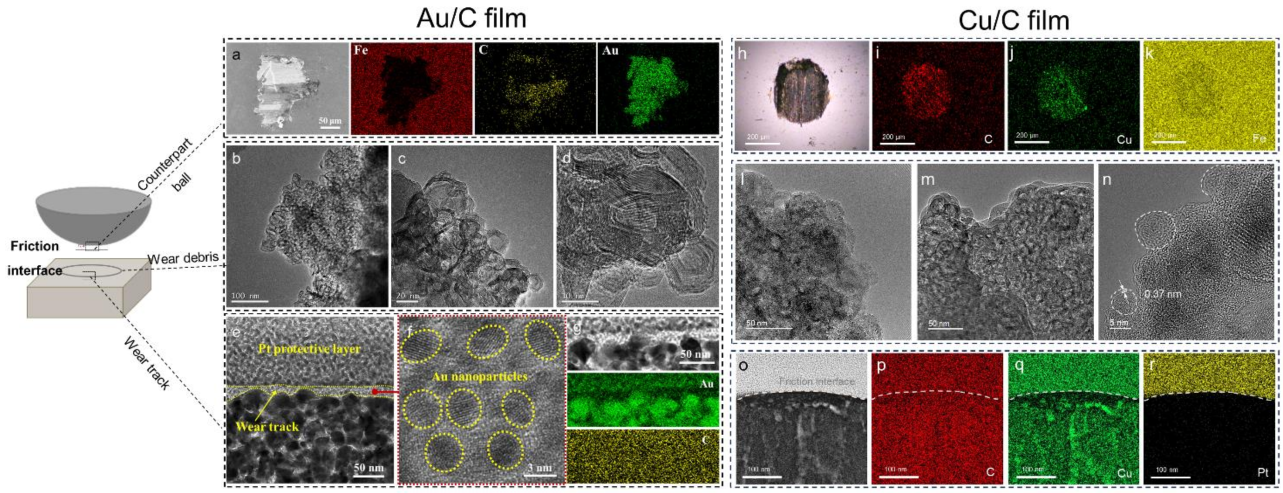

Fig. R4-1. Interfacial analysis of the Au/C film and Cu/C film after long-duration test. (a) The morphology and corresponding element distribution of the wear scar (the Au/C film). (b-d) TEM images of wear debris (the Au/C film). (e-g) TEM images and corresponding element distribution of cross-sections of the wear track (the Au/C film). (h) Optical morphology of wear scar (the Cu/C film). (i-k) Element distribution mapping of wear scar (the Cu/C film). (l-n) HRTEM images of wear debris (the Cu/C film). (o-r) TEM images and corresponding element distribution of cross-sections of the wear track (the Cu/C film).

4. The proposed intelligent Cu/C nanocomposite demonstrates impressive tribological performance over the 40 km test. However, material characterisation of both the ball and the wear track of the nanocomposite after this long-duration test is missing. This analysis is essential to confirm Cu

migration to the near-surface region of the composite film, the catalytic ordering of carbon within the worn track, and the presence of Cu transfer films on the ball. That information is essential to further support the proposed mechanistic framework for long-duration test.

Author reply: Thanks for this comment. We have supplemented the characterization of wear track of the Cu/C film, wear debris, and wear scar of the ball after long-duration test. As shown in Supplementary Fig. 20, cross-sectional HRTEM images combined with elemental mapping of the wear track clearly demonstrates that Cu migrates along specific channels toward the frictional interface and accumulates in the near-surface region. The surface morphology and elemental mapping of the counterpart ball confirm the presence of Cu transfer films on the counterpart ball. Furthermore, HRTEM image of the interfacial wear debris reveals the catalytic ordering of carbon within the worn track. Based on these, it provides direct evidence supporting the proposed mechanistic framework.

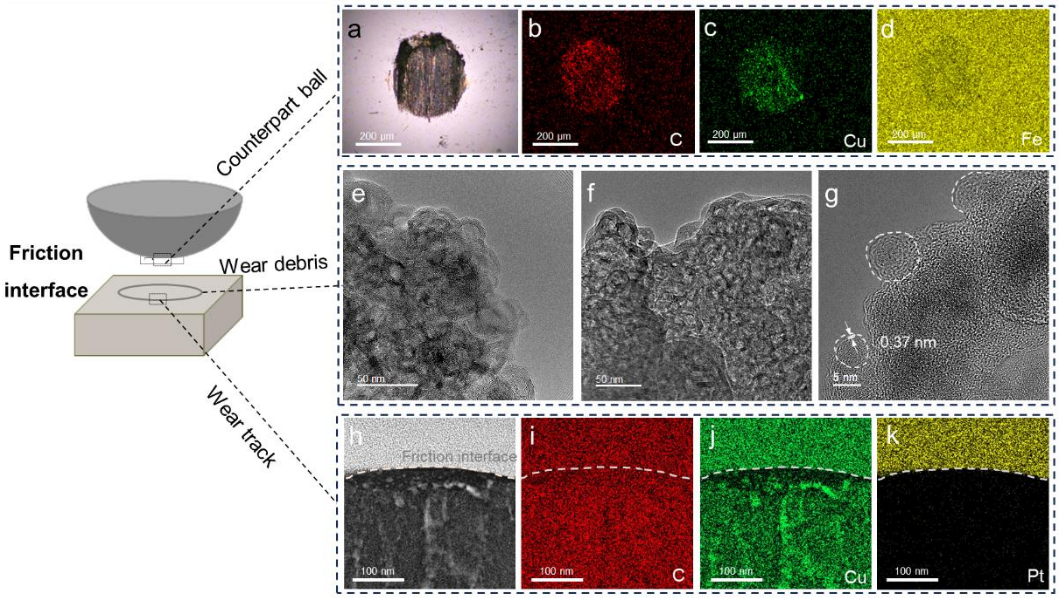

Supplementary Fig. 20. Interfacial analysis of the Cu/C film after long-duration test. (a) Optical morphology of wear scar. (b-d) Element distribution mapping of wear scar. (e-g) HRTEM images of wear debris. (h) TEM images and corresponding element distribution of cross-sections of the wear track.

5. It is unclear why the high-frequency reciprocating tests (6 Hz ≈ 9.4 cm/s and 9 Hz ≈ 14.2 cm/s) show significant different performance compared with the rotating test conducted at a higher linear speed (25.7 cm/s). Although the shear directions differ, rotation imposes a unidirectional shear while reciprocation alternates the sliding direction, the discrepancy in tribological behaviour is not fully explained. Specifically, it is puzzling why the high-frequency reciprocating tests, which still operate

at significantly lower linear speeds than the rotating test, result in high friction and failure.

Author reply: We thank the Reviewer for her/his insightful question, which prompted us to examine the underlying mechanisms in greater depth. The intelligent self-regulating lubrication state relies on a dynamic balance between the rate of metal migration to the interface and the rate at which the interfacial material is consumed. In this context, sliding speed primarily governs the migration rate, whereas sliding frequency strongly influences the consumption rate.

In high-frequency reciprocating motion, although the nominal linear sliding speed is lower than that in the rotating test, two unfavorable factors act simultaneously. **First**, the lower linear speed limits the efficiency of thermally driven metal migration compared with rotational sliding. **Second**, the much higher reciprocating frequency substantially increases the number of shear events per unit time. For example, 6 Hz and 9 Hz reciprocation correspond to 12 and 18 direction reversals per second, respectively, whereas rotation at 150 rpm corresponds to only about 2.5 shear cycles per second. This high-frequency alternation greatly accelerates interfacial consumption and disrupts the maintenance of a stable lubricating layer. The combined effect of insufficient migration and excessive consumption therefore leads to high friction and premature failure under high-frequency reciprocating conditions, despite the lower linear speeds.

This interpretation is further supported by the experimental evidence. As shown in Supplementary Fig. 2, increasing reciprocating frequency leads to shorter running-in times and reduced wear life for the Cu/C film. **On one hand**, higher frequencies generate greater frictional heating, which can initially promote rapid metal migration and quickly reduce friction. **On the other hand**, excessively high frequencies result in a consumption rate that exceeds the replenishment capacity, preventing the lubricating interface from reaching or sustaining equilibrium. This behavior is corroborated by the electrical resistance measurements. At frequencies below 3 Hz, the electrical resistance exhibits intermittent fluctuations, indicating a dynamic equilibrium with periodic self-adjustment. In contrast, at 3 Hz and above, the electrical resistance remains persistently high, reflecting continuous metal migration and depletion without stabilization of the interface.

Accordingly, the distinct tribological behaviors observed in high-frequency reciprocating tests and high-speed rotating tests arise from the combined and competing effects of sliding speed and sliding frequency on metal migration and consumption. We have incorporated this discussion into the revised manuscript to clarify the origin of the observed discrepancies.

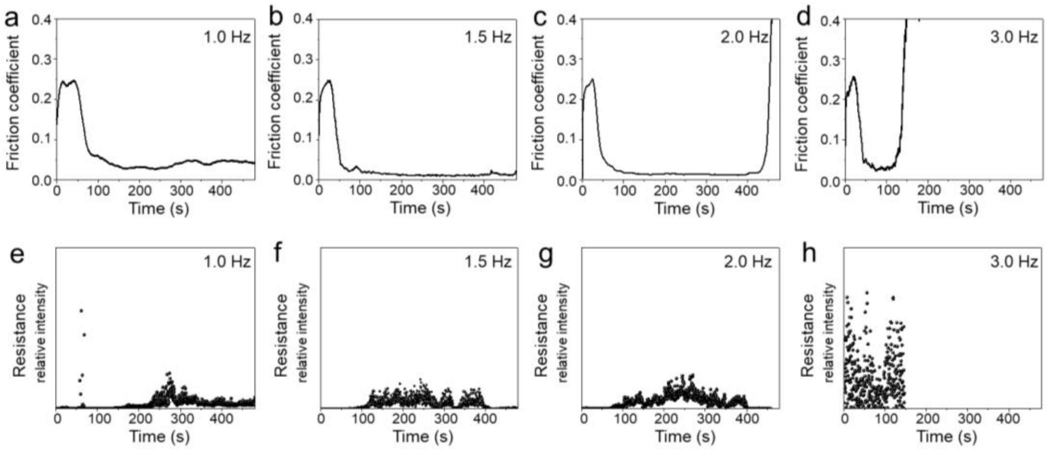

Supplementary Fig 2. Friction coefficient curves and corresponding electrical resistance at different reciprocating frequencies. (a, e) 1.0 Hz. (b, f) 1.5 Hz, (c, g) 2.0 Hz, (d, h) 3.0 Hz.

6. It is recommended to thoroughly improve the grammar and clarity of the descriptions in the supplementary document. For example, the statements 'Supplementary Fig. 2–4 depicts the relationship between friction coefficient (μ) and R at different friction frequencies is shown in' and 'From Supplementary Fig. 2 and 3, when the friction interface is in a metal-lack state (run-in state), during which the R values are relatively high' are grammatically incorrect and should be revised.

Author reply: Thanks for the Reviewer for pointing out these grammatical and clarity issues in the Supplementary Information. We have carefully revised and polished the language throughout both the manuscript and the Supplementary information to improve clarity, accuracy, and readability. All corrected sections have been clearly marked in the revised files.

Specifically, the sentence "Supplementary Fig. 2–4 depicts the relationship between friction coefficient (μ) and electrical resistance at different friction frequencies is shown in" should be "Supplementary Figs. 2–4 depict the relationship between friction coefficient (μ) and electrical resistance at different friction frequencies". Likewise, the sentence "From Supplementary Fig. 2 and 3, when the friction interface is in a metal-lack state (run-in state), during which the R values are

relatively high" should be "From Supplementary Figs. 2 and 3, when the friction interface is in a metal-lack state (run-in state), the R values are relatively high".

7. The information about how the amorphous and graphitic-like structures in Fig. 3f and 3i are generated is missing. It is unclear if that structure is generated from an MD simulation. The definition of reaction coordination should be clearly defined.

Author reply: Thanks for this comment. We clarify that the amorphous and graphitic-like structures shown in Fig. 3f and 3i (in original manuscript) were not taken from classical MD. Instead, they were generated using the DFT-based different melt–quench temperature method described in Supplementary Note 7. Briefly, the structures were obtained by heating to selected melt points followed by quenching and subsequent DFT relaxation to yield representative amorphous and graphitic-like configurations.

Regarding the reaction coordinate, as shown in the NEB energy profile in Fig. 3f, the horizontal axis represents a dimensionless reaction coordinate that describes the progress of the system along the minimum-energy reaction pathway obtained from the climbing-image nudged elastic band calculation. Specifically, this coordinate corresponds to the cumulative path length along the NEB band, constructed from a discrete set of intermediate images interpolated between the initial and final states. Each point on the curve corresponds to one optimized NEB image, and the coordinate increases monotonically from the initial to the final state. It reflects the accumulated atomic displacements along the reaction pathway rather than a real-space distance or physical time. The transition state is identified as the image with the highest energy along this reaction coordinate after the climbing-image refinement.

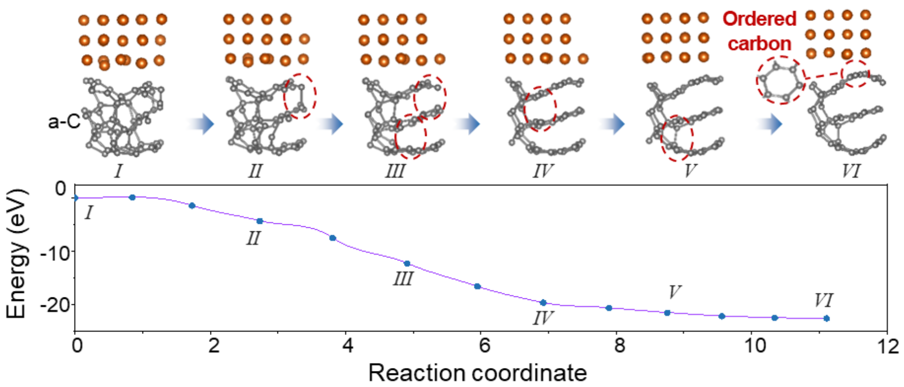

Fig. 3f. Energy changes in the Cu-catalyzed transformation from a-C to ordered carbon (*I*. a-C; *II*-*V*. sp^3C breakage and sp^2C recombination; *VI*. Ordered carbon).

8. The DFT-COHP analysis indicates C-C weakening in the presence of Cu. However, the calculation is based on a target C-C bond. This practice lowers the reliability of the calculation. It is recommended to provide the calculation that averages all of the C-C bonds or C-C bonds near the interface.

Author reply: We thank the Reviewer for her/his valuable suggestion. In response, we have revised the COHP analysis by averaging over all relevant C–C bonds, with particular emphasis on those located near the Cu–C interface, rather than focusing on a single target bond. As shown in Fig. 3i, the averaged ICOHP value for C–C bonds decreases from −11.15 eV in the Cu-free carbon system to −10.18 eV for C–C bonds in the presence of Cu. This systematic reduction indicates a general weakening of C–C bonding induced by Cu incorporation. Importantly, this averaged analysis demonstrates that the observed C–C weakening is a collective interfacial effect rather than an artifact of selecting a specific bond, thereby strengthening the reliability of the electronic-structure interpretation.

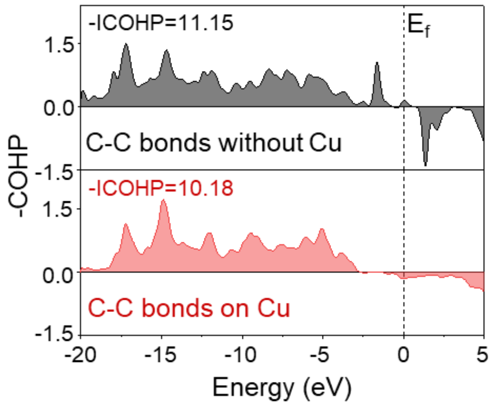

Fig. 3i. Averaged -COHP of C–C bonds with and without Cu NPs (The lower the absolute value of ICOHP, the weaker the C–C bond strength).

9. The reported material in the experiment is a Cu(Au)/C film; however, the simulation considers only a Cu/C system. Because Au nanoparticles are excellent catalysts that could significantly influence tribofilm formation, the authors need to provide a clear justification for their model selection (i.e., why only Cu was included) and explain the expected behaviour and contribution of Au.

Author reply: Thanks for this important comment. As clarified in our response to Comment 3, the Revised manuscript now focuses exclusively on the Cu/C system to ensure internal consistency between experiments and simulations. Accordingly, Cu was selected as the model system for atomistic

simulations because a complete and self-consistent experimental dataset is available for Cu/C, allowing direct and rigorous comparison between simulation and experiment.

We fully agree that Au nanoparticles can act as highly effective catalysts and may influence tribofilm formation and adaptive lubrication behavior. In our experiments, the Au/C film indeed exhibits qualitatively similar self-adjusting trends but with enhanced adaptability, which we attribute to its lower melting point and higher metal content, as discussed in the Revised manuscript. However, explicitly incorporating Au into the simulations would require additional, metal-specific parameterization and validation to ensure comparable reliability, which is beyond the scope of the present work.

We have clarified this modeling choice and the expected role of Au in the Revised manuscript. Guided by your valuable suggestion, we also note that a systematic comparative study incorporating a series of soft metals, such as Au and Ag, with varying melting points and catalytic activities, will be the focus of our future experimental and computational work.

10. The potential used in this work is adapted from Wang et al. [57], which primarily describes C-C interactions. Because this study focuses on the influence of Cu, an adequate and dedicated Cu-C dataset is needed to ensure that the potential can reliably capture Cu-C interactions.

The authors stated that "several representative Cu-C structures with varying compositional ratios were selected" and that "Cu-C surface models" were included. However, this description is highly generic and insufficient. A detailed description of the Cu-C training dataset is needed.

Importantly, for the simulations presented in the manuscript, (i) the migration of melted Cu nanoparticles within a conical pore in the a-C model, and (ii) Cu-nanoparticle-induced catalytic behaviour, it is not clear whether these specific atomic environments were explicitly included in the training set.

Author reply: We thank the Reviewer for raising this important point regarding the Cu–C training dataset. We agree that a clear and detailed description of the dataset is essential to assess the reliability of the developed potential. As shown in the Supplementary Fig. 24, although the initial dataset was

adapted from the simplified Wang *et al.* (*Physical Review B* **111,** 094205 (2025)). dataset for C–C interactions, the final NEP was trained on a substantially extended Cu–C and Cu dataset constructed specifically for this work. In particular, we independently constructed a dedicated pure Cu dataset, including bulk Cu, surface slabs configurations, to ensure an accurate description of Cu–Cu interactions. Beyond pure Cu and pure C reference structures, the training set explicitly includes Cu–C structures with varying compositional ratios, Cu–C interfacial and surface models, as well as Cu clusters interacting with carbon frameworks, which are directly relevant to the atomic environments explored in the simulations presented in this manuscript. These configurations were sampled from ab initio molecular dynamics (AIMD) simulations performed at temperatures ranging from 300 to 800 K for Cu bulk, Cu surface slabs, Cu–C bulk mixtures, Cu–C interfaces, and Cu-on-carbon surface systems, and were subsequently incorporated into the training dataset. As a result, the dataset explicitly covers (i) Cu surface interactions and coordination changes, and (ii) carbon structural reorganization in the presence of Cu, which are essential for accurately describing Cu nanoparticle melting, migration within confined carbon pores, and Cu-induced catalytic behavior. To further improve reproducibility, we publicly released the final trained NEP potential, together with training and test datasets, on Zenodo upon publication (https://doi.org/10.5281/zenodo.18051150). This will allow the community to fully assess and reuse the potential. In addition, we have supplemented detailed description of the Cu–C training dataset in the Revised manuscript and the Supplementary information.

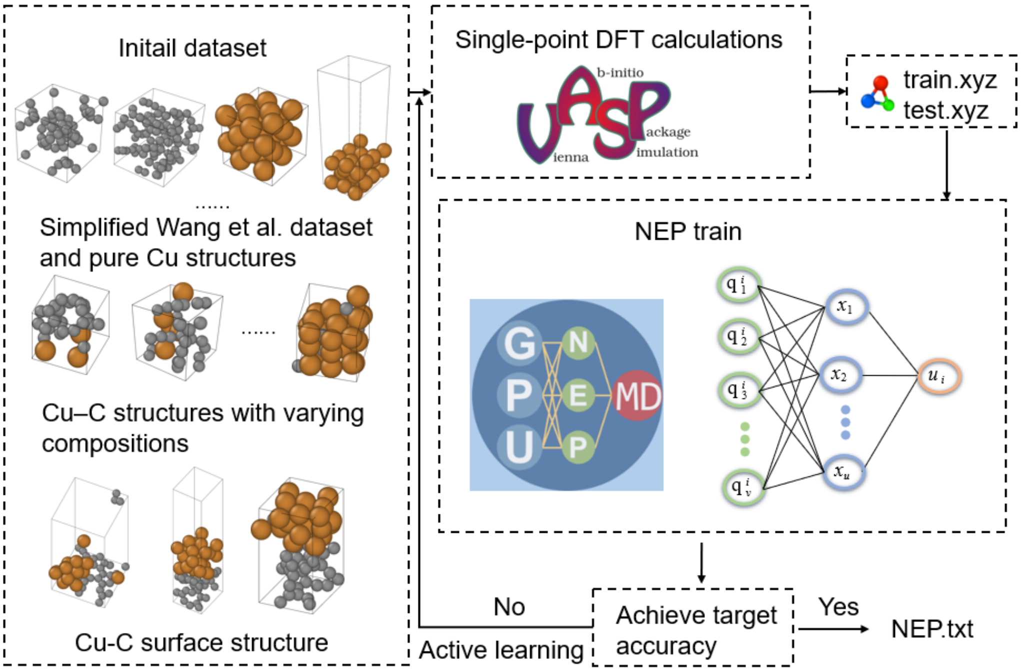

Supplementary Fig. 24. Schematic overview of the dataset construction and NEP training procedure used in this work.

11. The key behaviors examined in the MD simulation section rely on nanometer-scale Cu nanoparticles, whose properties differ substantially from bulk Cu. However, it remains unclear how the training dataset reflects the distinct physicochemical characteristics of nanosized Cu as opposed to bulk Cu.

The manuscript indicates that only Cu-C related structures were included in the training set, with no dedicated dataset for isolated nanoscale Cu clusters. It is necessary to validate if the developed/trained potential can indeed reproduce nanosized-Cu properties in MD simulation, such as the reduced melting point.

Author reply: Thanks for raising this important concern regarding the transferability of the potential to nanosized Cu clusters. We agree that nanosized Cu clusters exhibit physicochemical properties distinct from bulk Cu, and that validating the potential in this regime is essential.

Although the training dataset primarily includes bulk Cu and surface Cu slabs configurations, we further constructed independent nanoscale Cu clusters configurations (approximately 1 nm in size and containing 80 atoms) that were not included in the training set and used them as a validation benchmark.

As shown in Fig. R4-2, the developed NEP accurately reproduces the DFT reference data for these nanoscale Cu, yielding root-mean-square errors (RMSE) of 7.85 meV/atom for energies, 0.072 eV/Å for atomic forces, and 19.0 meV/atom for virials. These results indicate that the potential reliably captures Cu–Cu interactions across different local coordination environments beyond bulk phases.

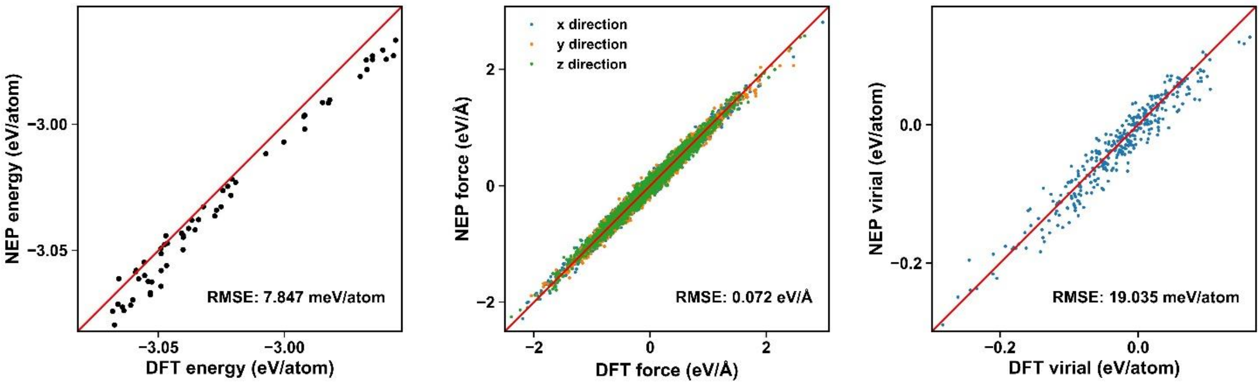

Fig. R4-2. Comparison between NEP predictions and DFT reference data for nanoscale Cu configurations. Parity plots of (a) energies, (b) atomic forces, and (c) virials, showing good agreement and indicating that the trained NEP accurately describes Cu–Cu interactions in nanometer-scale systems.

Following the reviewer's suggestion, we further explored the melting behavior of bulk Cu and isolated nanoscale Cu clusters (approximately spherical and ~1 nm in size) using MD simulations based on the developed NEP model. Bulk Cu was simulated under periodic boundary conditions, while Cu clusters were treated as isolated systems with non-periodic boundaries. All simulations were first equilibrated at 300 K and then gradually heated from 300 K to 2000 K in the NVT ensemble with a time step of 1 fs, and caloric curves were obtained from the potential energy–temperature relationship (see Fig. R4-3). For bulk Cu, the potential energy increases nearly linearly with temperature in the solid regime, followed by a clear change in slope and enhanced energy fluctuations in the temperature range of approximately 1300–1700 K, which corresponds to the melting transition in consistent with the experimental observations (*Materials & Design* **137**, 473-480 (2018)). In contrast, the Cu nanoparticles exhibit a similar but markedly shifted behavior. The solid-like regime persists only up to about 650 K, after which a pronounced change in the potential energy slope and fluctuations is observed in the range of approximately 650–800 K, indicating the onset of melting. The significantly lower transition temperature compared to bulk Cu clearly reflects the size-dependent melting-point

depression characteristic of nanoscale metal particles. These distinct energy discontinuities provide clear signatures of the solid–liquid transition for both systems and demonstrate that the developed NEP model is capable of capturing not only bulk Cu thermodynamics but also the reduced melting point of nanoscale Cu nanoparticles. Therefore, the NEP is suitable for describing the Cu nanoparticles involved in the MD simulations presented in this work.

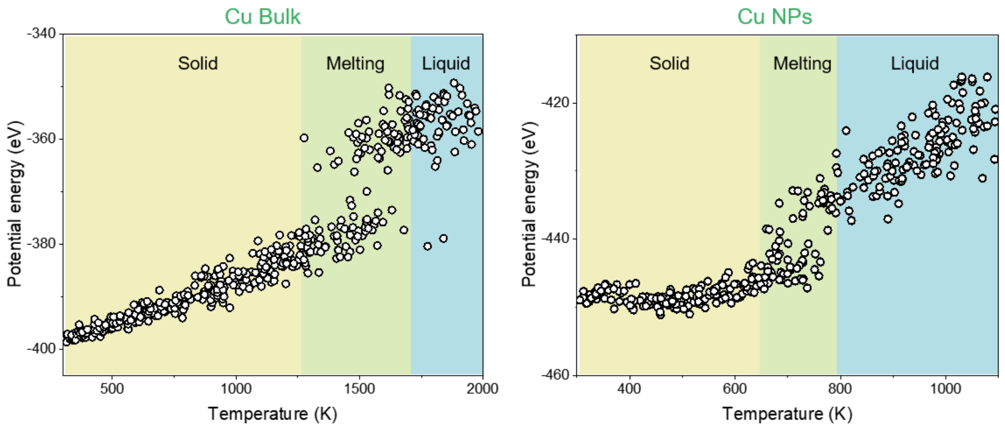

Fig. R4-3. Caloric curves (potential energy vs temperature) of bulk Cu and isolated Cu clusters obtained from NEP-based MD simulations. The shaded regions indicate the solid, melting (energy-transition) interval, and liquid regimes, highlighting the reduced melting point of nanoscale Cu compared with bulk Cu.
